# New Fluorescent Chemodosimetric Mechanism for Selective Recognition of Selenocysteine by Dansyl-Appended Ruthenium Nitrosyl Complexes

**DOI:** 10.1021/acs.inorgchem.4c05277

**Published:** 2025-02-20

**Authors:** Iván J. Bazany-Rodríguez, Pandiyan Thangarasu, M. Leticia Almada-Leyva, José Guadalupe Hernández, Diego Martínez-Otero, María K. Salomón-Flores, Alejandro Dorazco-González

**Affiliations:** † Facultad de Química, Universidad Nacional Autónoma de México, C.P., Coyoacán, Ciudad de México 04510, Mexico; ‡ Centro Tecnológico, Facultad de Estudios Superiores (FES-Aragón) UNAM, Nezahualcóyotl 57130, Estado de México, Mexico; § Centro Conjunto de Investigación en Química Sustentable, UAEM-UNAM, Toluca 50200, Estado de México, Mexico; ∥ Instituto de Química, Universidad Nacional Autónoma de México, Coyoacán, Ciudad de México 04510, Mexico

## Abstract

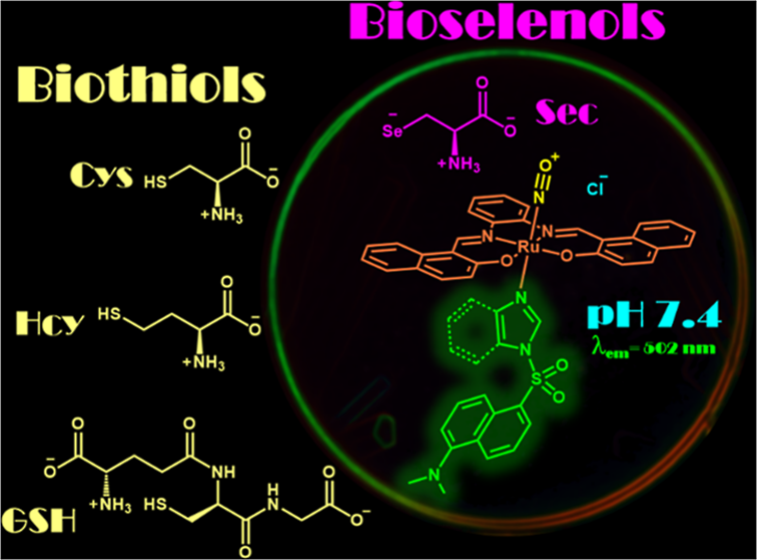

Selenocysteine (Sec) is a biologically essential amino acid that serves as a crucial component in selenoproteins that play a key role in various cellular functions. Thus, developing a reliable and rapid method for detecting Sec in physiological media is of paramount importance. This report introduces for the first time a novel fluorescent chemodosimetric mechanism for the selective recognition of Sec using dansyl-appended ruthenium nitrosyl complexes. These complexes consist of a tetradentate ligand featuring a π-extended system (**L** = *N*,*N*′-bis(2-hydroxy-1-naphthylidene)-1,2-phenylenediamine) and a monodentate ligand derived from the conjugated dansyl group, which acts as a strong fluorescent signaling unit (**ID** = dansyl-imidazole, **BD** = dansyl-benzimidazole). The reaction between Sec and the complexes {RuNO}^6^ = **[RuL(NO)(ID)]Cl** or **[RuL(NO)(BD)]Cl** in an aqueous phase enhances fluorescence; as a result, it releases NO^•^ that has been demonstrated through fluorimetric titrations, UV–vis titrations, ^77^Se NMR, EPR, IR, MS, and electronic density calculations. **[RuL(NO)(ID)]Cl** and **[RuL(NO)(BD)]Cl** quantitatively detect Sec within a micromolar concentration range, achieving the limit of detection as low as 0.31 and 0.12 μM, respectively, within just 5 min. Remarkably, these chemodosimeters can also be conveniently employed to detect Sec in living *Saccharomyces cerevisiae* cells.

## Abstract 2

Selenocysteine (Sec) is a biologically essential amino acid that serves as a crucial component in selenoproteins that play a key role in various cellular functions.

## Introduction

Efficient and selective optical chemosensing of bioselenols by chemodosimeters is an active topic in bioimaging, bioinorganic chemistry, and analytical chemistry due to their key functions in maintaining cellular physiological balance (such as antioxidant activity, anti-inflammatory activity, active thyroid hormone production, DNA synthesis, and anticancer properties).^1−8^ Among bioselenols, selenocysteine (Sec), considered the 21st amino acid and an analogue of cysteine (Cys), is the major functional form of selenium in biological systems.^9^ It is usually located in the active sites of selenoproteins, which affect many biological functions such as antioxidant activity, antiinflammation activity, active thyroid hormone production, DNA synthesis, transportation of cations, and cell growth.^10,11^ The inhibition or lack of Sec leads to a physiological and immune imbalance that, together with other factors, triggers various illnesses such as diabetes, cancer, nervous disorder, Keshan disorder, and Kashin-Beck disorder^12−17^

Optical recognition and detection of selenocysteine (Sec) have been dominated by synthetic organic fluorophores or chromophores that functionalize the chemodosimetric reaction site or modulate the sensing environment so that species such as biothiols do not interfere.^18−23^ For example, 2,4-dinitrobenzenesulfonate ester, 2,4-dinitrobenzene ether, 2,4-dinitrobenzenesulfonamide, benzoselenadiazole, disulfide bond, α,β-unsaturated carbonyl/ketone moiety, cyanine-labeled peptides, acrylate and acrylamide groups have been used for sensing of Sec in food or biological samples.^24−37^ However, some of these chemodosimeters only operate in organic-aqueous media or are incompatible with the biological environment (low hydro-stability, low photostability, low aqueous solubility, and operability in acidic medium), which seriously limits their intended applications.^38−55^ Furthermore, some of these chemodosimeters are not particularly selective, and interference from other compounds such as biothiols can be problematic. Besides, high detection limits that are unsuitable for detecting Sec in biological samples so that the shorter excitation and emission wavelengths of chemodosimeters cause photodamage to biological samples, as reported.^1,2,23,56,57^ Therefore, it is important to continue designing new optical Sec chemodosimeters to address these problems.

An alternative strategy for the Sec-selective sensing has been developed through a chemodosimetric mechanism based on the nitrosonium ligand (NO^+^) susceptibility to nucleophilic attacks.^58^ Ruthenium nitrosyls react with thiols (H_2_S, cysteine (Cys), glutathione (GSH), homocysteine (Hcy), *N*-acetylcysteine, and others) to give NO^•^ and/or HNO.^59^ With this line of research, a {RuNO}^6^ complex has been shown to be highly selective for the detection of H_2_S, releasing NO^•^ after reacting with H_2_S.^60^ Based on this molecular strategy, the NO^+^ ligand involved in ruthenium complexes can be susceptible to nucleophilic attack by the selenol group of Sec due to its better nucleophilicity over that of biothiols (Cys, Hcy, and GSH).^61,62^ Additionally, due to a lower p*K*_a_ of selenol in Sec (∼5.24)^63^ compared to aliphatic biothiols in Cys (∼8.44),^64^ GSH (∼8.60),^65^ Hcy (∼8.87),^66^ Sec is expected to exhibit greater reactivity than the biothiols under physiological conditions (pH ∼7.4), which means that the selenol (R–SeH) in Sec is almost fully presented as the selenolate (R–Se^–^), while the majority of thiols have existed as less reactive nonionized forms (R–SH), so the release of NO^•^ in ruthenium nitrosyl complexes is much slower. Indeed, such a difference in p*K*_a_ allows for the selective detection of Sec even in the presence of biothiols.

**Scheme 1:**
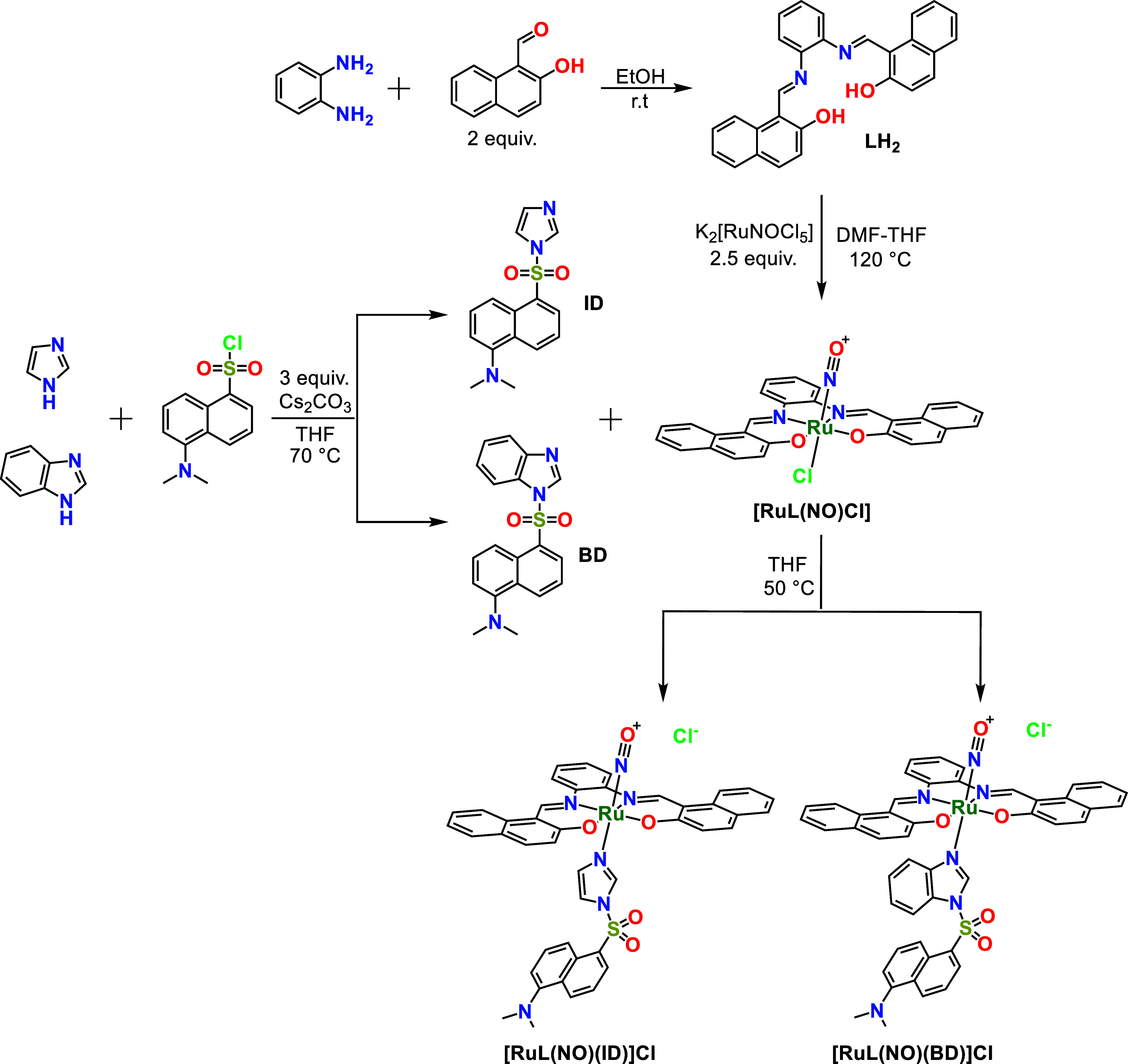
General Synthesis of the Ligands and Complexes

**Scheme 2:**
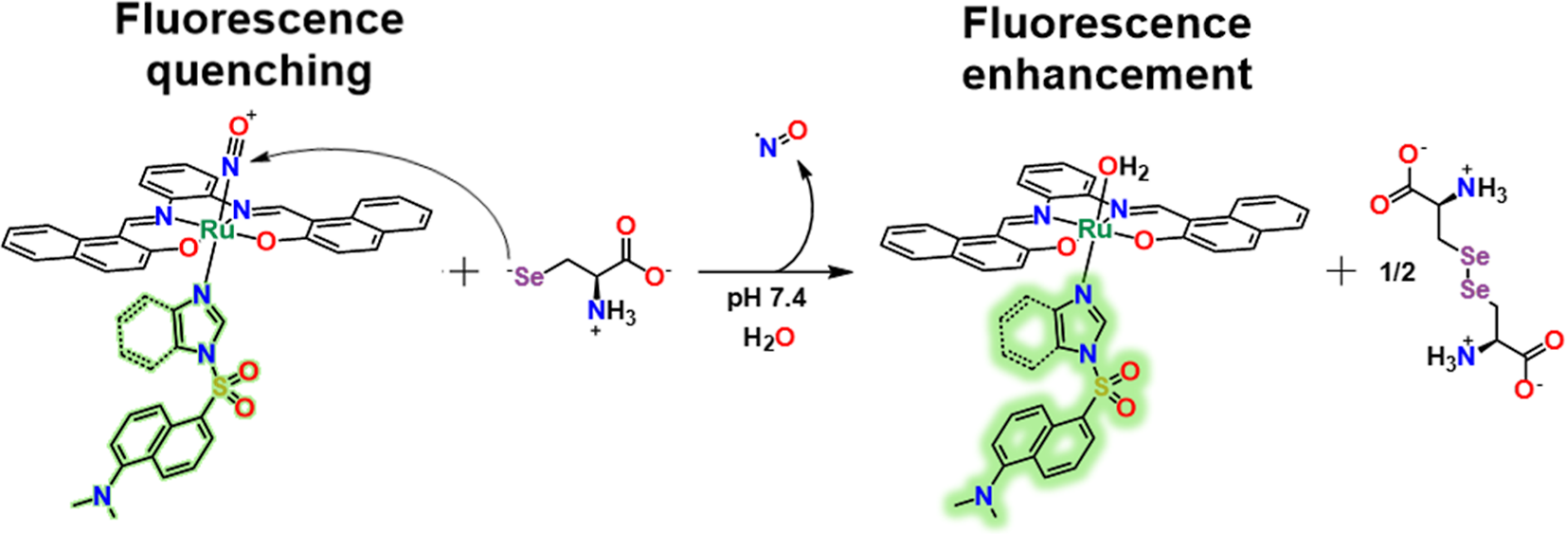
Proposed Sensing Mechanism of **[RuL(NO)(BD)]**^**+**^ and **[RuL(NO)(ID)]**^**+**^ toward Sec

Thus, we explored the use of two {RuNO}6 complexes, **[RuL(NO)(ID)]**^**+**^ and **[RuL(NO)(BD)]**^**+**^, as chemodosimeters for the turn-on fluorescent detection of Sec in aqueous solution under physiological conditions (pH ∼ 7.4). The new complexes, **[RuL(NO)(ID)]**^**+**^ and **[RuL(NO)(BD)]**^**+**^, contain a tetradentate ligand bearing the π-extended system, (**LH**_**2**_ = *N*,*N*′-bis(2-hydroxy-1-naphthylidene)-1,2-phenylenediamine) and a monodentate ligand derived from the conjugated dansyl group that acts as a strong fluorescent signaling unit (**ID** = dansyl-imidazole and **BD** = dansyl-benzimidazole) when the Sec reacts with {RuNO}^6^, releasing NO^•^ (Schemes 1 and 2). In addition, the chemodosimetric mechanism consists of the reaction between the selenolate in Sec and the electrophilic nitrosyl ligands of **[RuL(NO)(ID)]**^**+**^ or **[RuL(NO)(BD)]**^**+**^. This can be best described as a nitrosonium species (NO^+^), with the N atom being the site for the nucleophilic addition to Sec. So, both NO^•^ and Selenocystine (Sec_2_), as well as the respective fluorescent aqua-complexes of ruthenium(II), **[RuL(OH**_**2**_**)(ID)]** or **[RuL(OH**_**2**_**)(BD)]** are the proposed products in the redox reaction (Scheme 2). The results obtained for the fluorescent chemodosimeters based on {RuNO}^6^ complexes, including spectroscopic sensing of Sec in buffered solution at pH 7.4, fluorescence imaging of Sec in cells, and theoretical DFT studies, are summarized below.

## Experimental Section

### General Considerations

The Supporting Information section lists chemical reagents, solvents, and instruments.

### Synthesis of Dansyl-Imidazole (**ID**) and Dansyl-Benzimidazole (**BD**)

Dansyl ligands were prepared according to the literature method with some modifications.^67^ To a solution of dansyl chloride (100 mg, 0.37 mmol) in dry THF (25 mL) were added imidazole (25 mg, 0.37 mmol) or benzimidazole (44 mg, 0.37 mmol) and Cs_2_CO_3_ (381 mg, 1.17 mmol). The reaction mixtures were allowed to stir at 70 °C overnight and filtered in vacuo, and the solvent was removed by rotary evaporation. Crystallization of the crude products from EtOAc and *n*-hexane 1:5 (v/v) gave greenish-yellow needles of **ID** (∼106 mg, 95%) and **BD** (∼113 mg, 87%). **ID**; ^1^H NMR (300 MHz, DMSO-*d*_6_): δ 8.65 (dt, *J* = 8.6, 1.0 Hz, 1H), 8.55 (dd, *J* = 1.4, 0.9 Hz, 1H), 8.45 (dd, *J* = 7.5, 1.2 Hz, 1H), 8.23 (dt, *J* = 8.7, 0.8 Hz, 1H), 7.80–7.75 (m, 2H), 7.67 (t, 1H), 7.30 (dd, *J* = 7.7, 0.7 Hz, 1H), 7.08 (dd, *J* = 1.6, 0.9 Hz, 1H), 2.83 (s, 6H) ppm. **BD**; ^1^H NMR (300 MHz, DMSO-*d*_6_): δ 9.20 (s, 1H), 8.72 (d, *J* = 7.5 Hz, 1H), 8.61 (d, *J* = 8.5 Hz, 1H), 8.28 (d, *J* = 8.6 Hz, 1H), 7.78 (t, 1H), 7.73–7.62 (m, 3H), 7.37–7.30 (m, 2H), 7.26 (d, *J* = 7.6 Hz, 1H), 2.78 (s, 6H) ppm.

### Synthesis of *N*,*N*′-Bis(2-hydroxy-1-naphthylidene)-1,2-phenylenediimine (**LH**_**2**_)

Ligand **LH**_**2**_ was prepared according to the literature method with some modifications.^68,69^ 2-Hydroxy-1-naphthaldehyde (100 mg, 0.58 mmol) in dry EtOH (25 mL) was added to a solution of 1,2-phenylendiamine (31.4 mg, 0.29 mmol) in dry EtOH (25 mL) at room temperature. After being stirred for 4 h, the resulting solid was separated by filtration, washed with cold EtOH, dried in vacuo, and recrystallized from EtOH/EtOAc 1:1 (v/v) to get the corresponding naphophen-H_2_ ligand (**LH**_**2**_) as an orange crystalline powder (∼110 mg, 92%). ^1^H NMR (300 MHz, DMSO-*d*_6_): δ 15.13 (s, 2H), 9.70–9.69 (d, *J* = 3.6 Hz, 2H), 8.56–8.53 (d, *J* = 8.3 Hz, 2H), 7.98–7.95 (d, *J* = 9.3 Hz, 2H), 7.85–7.82 (m, 4H), 7.58–7.53 (t, 2H), 7.46–7.43 (m, 2H), 7.40–7.35 (t, 2H), 7.08–7.05 (d, *J* = 9.1 Hz, 2H) ppm.

### Synthesis of **[RuL(NO)Cl]**

Complex **[RuL(NO)Cl]** was prepared using the literature method with some modifications.^70,71^ In a Schlenk flask, K_2_[RuNOCl_5_] (100 mg, 0.26 mmol) was dispersed in dry DMF (2 mL). To this dispersion were added the tetradentate ligand **LH**_**2**_ (108 mg, 0.26 mmol) and a little excess of NEt_3_ (109 μL, 0.78 mmol) dissolved in dry THF (20 mL) dropwise; then, the reaction mixture was heated at 120 °C with vigorous stirring under dark conditions for 4 h, during which a color change from orange to brown occurred. The resulting solution was concentrated in vacuo to approximately 2 mL. Adding water (50 mL) to the reaction solution gave a reddish-brown precipitate (∼104 mg, 69%), which was filtered and washed with EtOAc. ^1^H NMR (300 MHz, DMSO-*d*_6_): δ 10.09 (s, 2H), 8.71–8.67 (m, 4H), 8.06–8.03 (d, *J* = 9.3 Hz, 2H), 7.87–7.84 (d, *J* = 7.8 Hz, 2H), 7.65–7.61 (t, 2H), 7.56–7.53 (m, 2H), 7.43–7.38 (t, 2H), 7.36–7.33 (d, *J* = 9.3, 2H) ppm. IR (ATR): 1816 (ν N≡O^+^), 1664 and 1613 (ν C=N), 1573 and 1533 (ν Aryl C–C), 1360 (ν Ar–O), 744 (ν Aryl C–H), 558 (ν Ru–N), 498 (ν Ru–O) cm^–1^.

### Synthesis of **[RuL(NO)(ID)]Cl** and **[RuL(NO)(BD)]Cl**

To a solution of **[RuL(NO)Cl]** (40 mg, 0.07 mmol) in dry THF (5 mL) was added dropwise a solution of the corresponding dansyl ligand (**ID**: 21 mg, 0.07 mmol; **BD**: 25 mg, 0.07 mmol) in dry THF (5 mL). The reaction mixtures were allowed to stir at 50 °C for 30 min. These solutions of the complexes were allowed to slowly evaporate at room temperature to give reddish-brown microcrystals of **[RuL(NO)(ID)]Cl** (∼59 mg, 95%) and **[RuL(NO)(BD)]Cl** (∼63 mg, 97%). **[RuL(NO)(ID)]Cl**; ^1^H NMR (300 MHz, DMSO-*d*_6_): δ 10.09 (s, 1H), 9.07 (s, 1H), 8.70–8.67 (d, *J* = 9.2 Hz, 4H), 8.56–8.53 (d, *J* = 8.6 Hz, 1H), 8.18–8.15 (d, *J* = 8.5 Hz, 1H), 8.06–8.03 (d, *J* = 9.3 Hz, 2H), 7.96–7.94 (d, *J* = 5.9 Hz, 1H), 7.86–7.84 (d, *J* = 7.1 Hz, 2H), 7.68 (s, 2H), 7.65–7.61 (t, 2H), 7.55–7.52 (m, 2H), 7.44–7.33 (m, 6H), 7.13–7.10 (d, *J* = 7.4 Hz, 1H), 2.80 (s, 6H), ppm. ^13^C NMR (75 MHz, DMSO-*d*_6_): δ 171.46, 161.04, 152.70, 150.24, 148.83, 144.47, 143.23, 138.52, 135.01, 134.40, 130.43, 129.13, 128.73, 128.53, 128.00, 127.00, 125.36, 125.08, 124.83, 124.32, 123.60, 123.53, 122.63, 121.48, 119.36, 117.60, 113.86, 110.34, 45.12, ppm. IR (ATR): 3327 and 3137 (ν N–CH_3_),1855 (ν N≡O^+^), 1612 (ν C=N), 1572 and 1533 (ν Heteroaryl C–N), 1358 (ν Ar–O), 1336 and 1184 (ν SO_2_–N), 1057 (ν O=S=O), 750 (ν Aryl C–H), 634 (ν C–S), 560 (ν Ru–N), 499 (ν Ru–O) cm^–1^. MS (MALDI-TOF^+^) *m*/*z*: 847.1230, **[RuL(NO)(ID)]**^+^, [C_43_H_33_N_6_O_5_RuS]^+^. Elemental analysis calculated for C_43_H_33_ClN_6_O_5_RuS·3H_2_O (%): C, 55.16; H, 4.20; Cl, 3.79; N, 8.98; O, 13.67; Ru, 10.79; S, 3.42. Found: C, 55.18; H, 4.26; N, 8.97; S, 3.45. **[RuL(NO)(BD)]Cl**; ^1^H NMR (300 MHz, DMSO-*d*_6_): δ 10.09 (s, 2H), 9.44 (s, 1H), 8.70–8.67 (d, *J* = 8.5 Hz, 4H), 8.60–8.58 (d, *J* = 8.5 Hz, 1H), 8.19–8.16 (d, *J* = 8.4 Hz, 1H), 8.05–8.02 (d, *J* = 9.3 Hz, 2H), 7.98–7.96 (d, *J* = 7.0 Hz, 1H), 7.86–7.82 (m, 4H), 7.65–7.60 (t, 2H), 7.57–7.52 (m, 4H), 7.46–7.33 (m, 6H), 7.18–7.15 (d, *J* = 7.4 Hz, 1H), 2.83 (s, 6H), ppm. ^13^C NMR (75 MHz, DMSO-*d*_6_): δ 171.44, 152.66, 149.52, 144.48, 143.20, 140.75, 138.49, 134.99, 131.14, 130.39, 129.10, 128.49, 127.96, 126.97, 125.71, 125.32, 124.86, 124.81, 124.38, 123.76, 123.49, 123.03, 121.45, 117.57, 114.53, 114.11, 110.32, 45.19, ppm. IR (ATR): 3068 and 3054 (ν N–CH_3_), 2922 (ν CH_3_), 1821 (ν N≡O^+^), 1599 (ν C=N), 1573 and 1533 (ν Heteroaryl C–N), 1360 (ν Ar–O), 1360 and 1187 (ν SO_2_–N), 1164 (ν O=S=O), 743 (ν Aryl C–H), 681 (ν C–S), 558 (ν Ru–N), 498 (ν Ru–O) cm^–1^. MS (MALDI-TOF^+^): *m*/*z* 897.1330, **[RuL(NO)(BD)]**^+^, [C_47_H_35_N_6_O_5_RuS]^+^. Elemental analysis calculated for C_47_H_35_ClN_6_O_5_RuS·H_2_O (%): C, 59.40; H, 3.92; Cl, 3.73; N, 8.84; O, 10.10; Ru, 10.63; S, 3.37. Found: C, 59.42; H, 3.96; N, 8.88; S, 3.38.

### Spectrophotometric and Fluorometric Studies

The Sec stock solution (0.5 mM) was prepared by the reaction of equimolar amounts of selenocystine (Sec)_2_ and dithiothreitol (DTT) in 20 mM phosphate buffer solution (PBS) at pH 7.4 and 37 °C for 30 min and was freshly used. The stock solutions of other bioanalytes such as NaCl, KCl, NaI, MgCl_2_, CaCl_2_, Na_2_SO_4_, NaHCO_3_, Na_2_HPO_4_, NaOAc, NaHS, Cys, Hcy, and GSH were prepared in buffered aqueous solution (HEPES 20 mM at pH 7.4). The selectivity experiments were performed by adding aliquots of stock solutions of the respective bioanalyte, final concentration of [Bioanalyte]_final_ = 40 μM, to buffered aqueous solutions containing HEPES (20 mM at pH 7.4) of **[RuL(NO)(ID)]Cl** (10 μM) and **[RuL(NO)(BD)]Cl** (10 μM), so the emission maximum intensities of **[RuL(NO)(ID)]Cl** and **[RuL(NO)(BD)]Cl** were recorded. In addition, the detection ability of chemodosimeters for pH interference was performed at different pHs (MES 20 mM at pH 6.5; HEPES 20 mM at pH 7.4; TRIS 20 mM at pH 8.5). The titration experiments were performed by adding aliquots of stock solutions of analytes to a buffered aqueous solution containing HEPES (20 mM at pH 7.4) of **[RuL(NO)(ID)]Cl** (10 μM) and **[RuL(NO)(BD)]Cl** (10 μM). After the analytes were added, the solution was allowed to react for 5 min with vigorous stirring at room temperature before recording the absorption spectrum and the emission spectrum (excitation wavelength at 340 nm) using a 10 mm quartz cuvette.

### ^77^Se NMR Studies

The NMR experiments were performed by using a 300 MHz spectrometer. The ^77^Se NMR spectra were recorded after an aliquot of the respective chemodosimeter was added to a solution of Sec (10.0 mM) in 0.5 mL of DMSO–D_2_O (1:5 v/v; 200 mM PBS at pD 7.4) directly into an NMR tube.

### IR Spectroscopy Studies

IR spectra were recorded after adding an aliquot of Sec (20 mM; 200 mM PBS at pH 7.4) to a solution of the respective chemodosimeter (5.0 mM) in 3.0 mL of MeOH–H_2_O (2:1 v/v). After vigorously stirring the reaction mixture for 15 min, the solvents were completely removed, and then the powders were analyzed by IR spectroscopy.

### EPR Studies

EPR experiments were performed to detect the NO^•^ from Na_2_[Fe^II^(PDTC)_2_–NO^•^] by using spin trap EPR spectroscopy.^72−74^ Na_2_[Fe^II^(PDTC)_2_] was prepared by adding FeSO_4_·7H_2_O (5.0 mM) and Na_2_PDTC (10.0 mM) (PDTC = l-proline dithiocarbamate) in situ with nitrogen-purged deionized water. **RuL(NO)(ID)]Cl** or **RuL(NO)(BD)]Cl** solution (100 μL of 5.0 mM in CH_3_CN), respectively, is mixed with Na_2_[Fe^II^(PDTC)_2_] (30 μL of 5.0 mM in H_2_O) having Sec (400 μL of 5.0 mM in PBS (200 mM) at pH 7.4). The solution mixture was injected into quartz capillaries for EPR experiments. Disodium l-proline dithiocarbamate (Na_2_PDTC) was synthesized using the general procedure developed for the synthesis of substituted dithiocarbamates.^75,76^ Briefly, CS_2_ (523 μL, 5.8 mmol) was dissolved in dry Et_2_O (20 mL), and the mixture cooled to 0 °C. l-proline (500 mg, 4.3 mmol) and NaOH (347.5 mg, 8.7 mmol) were dissolved in dry MeOH (10 mL) and added dropwise to the CS_2_ solution. The reaction mixture was stirred for 5 h at 0 °C. The solvent was removed by a rotary evaporator, and the resulting residue was triturated with Et_2_O. The white solid filter was washed with diethyl ether and dried in vacuo to yield 633 mg (62%) of Na_2_PDTC.

### Crystallographic Experiments

Crystal data for **BD** and **ID** were collected on a Bruker APEX II CCD Diffractometer at 100 K, using Mo K_α_ radiation (λ = 0.71073 Å) from an Incoatec ImuS source and Helios optic monochromator. Suitable crystals were coated with hydrocarbon oil, picked up with a nylon loop, and mounted in a cold nitrogen stream of the diffractometer. Frames were collected using ω scans and integrated with SAINT.^77^ Multiscan absorption correction (SADABS) was applied. The structures were solved by direct methods and refined using full-matrix least-squares on *F*^2^ with SHELXL-2018^78^ using the SHELXLE GUI.^79^ The hydrogen atoms of the C–H bonds were placed in idealized positions and refined with *U*_iso_ = *aU*_eq_ (where *a* is 1.5 for –CH_3_ and 1.2 for others). Crystallographic data for the two crystal structures have been deposited with the Cambridge Crystallographic Data Centre, CCDC 2302041 and 2302035.

### Cell Imaging

To investigate the imaging capability of the complexes with cells, chemodosimeters toward Sec in live *Saccharomyces cerevisiae* cells were monitored, for which yeast cells were first grown in Muller Hinton Broth (MHB); subsequently, yeast cell imaging experiments were designed into four groups. (I) The first group is a control experiment, i.e., yeast cells were first incubated with 10 μM of each chemodosimeter for 40 min, and then images were captured after the cells were washed 3 times with PBS buffer. (II) Yeast cells were preincubated with Sec (10.0 μM) for 40 min to remove excess Sec, followed by the addition of 10.0 μM of each chemodosimeter to the above solution and then confocal imaging was taken after coincubation for 40 min; the free sensor was removed with PBS buffer. Finally, (III and IV) yeast cells were first incubated with 20.0 or 40.0 μM Sec at 37 °C for 30 min to remove excess Sec, respectively, and then 10.0 μM of each chemodosimeter was added to each group of cells and coincubated for 40 min to remove free chemodosimeter for imaging; excess chemodosimeter was removed with PBS buffer. A confocal fluorescence microscope (λ_ex_ = 405 nm) observed each group of cells.

## Results and Discussion

### Molecular Design and Synthesis of Ruthenium–Nitrosyl Complexes with Dansyl-Appended

Given the extremely important role of Sec in various cellular functions and human diseases related to diabetes and cancer, several research groups have strived to create chemosensors and chemodosimeters to detect Sec. To date, various reaction mechanisms have been used for the design of some luminescent chemodosimeters for Sec (selenium–sulfur exchange reactions, acrylate addition reactions, and nucleophilic aromatic substitution reactions of 2,4-dinitrobenzenesulfonamide and 2,4-dinitrobenzenoxy). However, the development of luminescent chemosensors and chemodosimeters for Sec detection without interference from biothiols (Cys, Hcy, and GSH) remains challenging because of their similar chemical characteristics. Therefore, in the present work, as a new strategy for the luminescent detection of Sec, a new chemodosimetric reaction mechanism was developed based on the susceptibility of {RuNO}^6^ to nucleophilic attack. Thus, two {RuNO}^6^ complexes, **[RuL(NO)(ID)]**^**+**^ and **[RuL(NO)(BD)]**^**+**^, were designed and synthesized as chemodosimeters for the turn-on fluorescent detection of Sec in aqueous solution under physiological conditions (pH ∼ 7.4).

Scheme 1 provides the synthesis route of cationic octahedral complexes **[RuL(NO)(ID)]Cl** and **[RuL(NO)(BD)]Cl**. **LH**_**2**_ was prepared by a condensation reaction between 1,2-phenylenediamine and 2-hydroxy-1-naphthaldehyde in EtOH. This ligand employs two imine-N donors (in addition to two phenolate-O donors) to bind the ruthenium nitrosyl center. **[RuL(NO)Cl]** was synthesized by a chelation reaction between **LH**_**2**_ and K_2_[RuNOCl_5_] in THF-DMF. As shown in Scheme 2, the planar tetradentate ligand (**LH**_**2**_) chelates, as a dianion, the ruthenium nitrosyl unit and occupies the equatorial plane, leaving an axial position open for binding of a monodentate ligand. Furthermore, **L** presents a π-extended system, which effectively contributes to increasing the reactivity of {RuNO}^6^ toward reductant nucleophiles such as thiolates or selenolates.^71,80−82^

**Figure 1:**
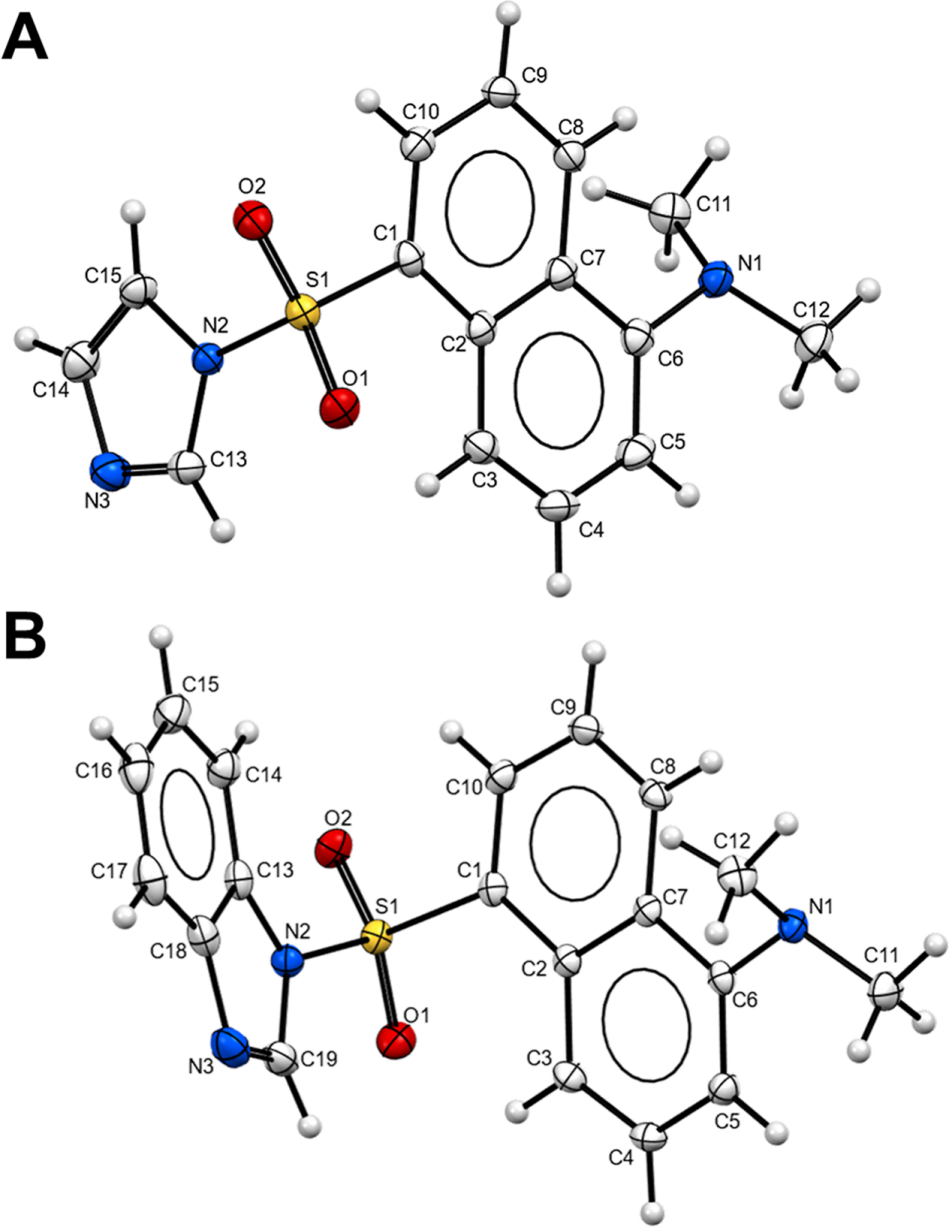
ORTEP diagrams of (A) **ID** and (B) **BD** showing 50% probability thermal ellipsoids.

The monodentate ligands, dansyl-imidazole (**ID**) and dansyl-benzimidazole (**BD**), were prepared by a nucleophilic substitution reaction of imidazole or benzimidazole with dansyl chloride and Cs_2_CO_3_ in THF. Crystallization of the crude products from EtOAc and *n*-hexane 1:5 (v/v) gave **ID** and **BD** greenish-yellow needles. X-ray diffraction quality crystals of **ID** and **BD** were grown over 5 days, and crystallographic data are summarized in Tables S1 and S2. The crystal structures of **ID** and **BD** are shown in Figure 1. The crystal structure of **ID** has been reported,^83^ but for **BD**, it is the first crystallographically characterized dansyl fluorophore containing a benzimidazole heterocycle. In the ligand **BD**, the dihedral angle between the naphthalene ring system and the benzimidazole ring is 81.35 (4)°, and for **ID**, the dihedral angle between the naphthalene ring system and the imidazole ring is 86.11 (2)°. In both crystal structures, weak intermolecular C–H···O and C–H···N hydrogen bonds, as well as weak C–H···π interactions, connect molecules, forming a two-dimensional network.

Finally, chemodosimeters based on cationic {RuNO}^6^ octahedral complexes, **[RuL(NO)(ID)]Cl** and **[RuL(NO)(BD)]Cl**, were easily prepared from solutions of the precursor **[RuL(NO)Cl]** and the respective conjugated dansyl ligand, **ID** or **BD**, by the replacement of the chloride (trans to NO^+^) ligand in THF at 50 °C. The dansyl ligands were directly coordinated to the Ru^II^ centers of the {RuNO}^6^ complexes. **ID** and **BD** are potent fluorophores; however, when they are coordinated to **[RuL(NO)Cl]** to form **[RuL(NO)(ID)]Cl** and **[RuL(NO)(BD)]Cl**, their fluorescence is significantly quenched; upon release of NO^•^ caused by the nucleophilic attack of Sec, the aqua-complex products, **[RuL(OH**_**2**_**)(ID)]** and **[RuL(OH**_**2**_**)(BD)]**, support the enhancement of the dansyl fluorophore, allowing, thus, a highly sensitive fluorometric chemodosimetric method to detect Sec. Both the tetradentate ligand (**L**) and the conjugated dansyl ligands (**ID** and **BD**) trans to NO^+^ contribute to increasing the reactivity of {RuNO}^6^ toward reductant nucleophiles. Mainly because the π-extended conjugated systems of equatorial tetradentate ligand **L**, and the modest π-withdrawing ability of the conjugated dansyl ligand trans to NO^+^ are contributing to the bathochromic shift of the dπ(Ru) → π*(Ru–NO^+^) MLCT band, promoting that the reduction potential of Ru^II^(NO^+^)/Ru^II^(NO^0^) can be more positive and, consequently, be susceptible to nucleophilic attack by reductants such as Sec.^84−92^ The effects of π-extended conjugation on the equatorial ligand **L** and the fluorophore **ID** or **BD** bounded to the metal are additive.^93,94^ It is well-accepted that the reactivity of {RuNO}^6^ to reductant nucleophiles depends on the energy of the dπ(Ru) → π*(Ru–NO^+^) electronic transition that is directly connected to the redox potential of the Ru^II^(NO^+^)/Ru^II^(NO^0^) couple.^95,96^ Thus, the Ru^II^(NO^+^)/Ru^II^(NO^0^) redox potential reflects the electron density of the NO^+^ ligand; therefore, it would be directly related to the susceptibility of the NO^+^ ligand to nucleophilic attack.

### Sec Recognition and Fluorescent Detection

Taking advantage of the fact that {RuNO}^6^ complexes react with reductant nucleophiles, **[RuL(NO)(ID)]Cl** and **[RuL(NO)(BD)]Cl** were studied as fluorescent chemodosimeters for Sec by different spectroscopic experiments. The NO^+^ ligand involved in {RuNO}^6^ complexes can be reduced to NO^•^ and released by the selenol group of Sec, due to its better nucleophilicity and lower p*K*_a_ than that of biothiols (Cys, Hcy, and GSH). In a reaction medium at pH ∼ 7.4, the high degree of dissociation of Sec results in the predominant generation of the corresponding selenolate (RSeH; p*K*_a_ ∼ 5.24), which can effectively react with **[RuL(NO)(ID)]Cl** and **[RuL(NO)(BD)]Cl**. However, under the same conditions, the less reactive neutral form of biothiols predominates (RSH; p*K*_a_ ≥ 8.4), so the release of NO^•^ in **[RuL(NO)(ID)]Cl** and **[RuL(NO)(BD)]Cl** is less rapid.

**Figure 2:**
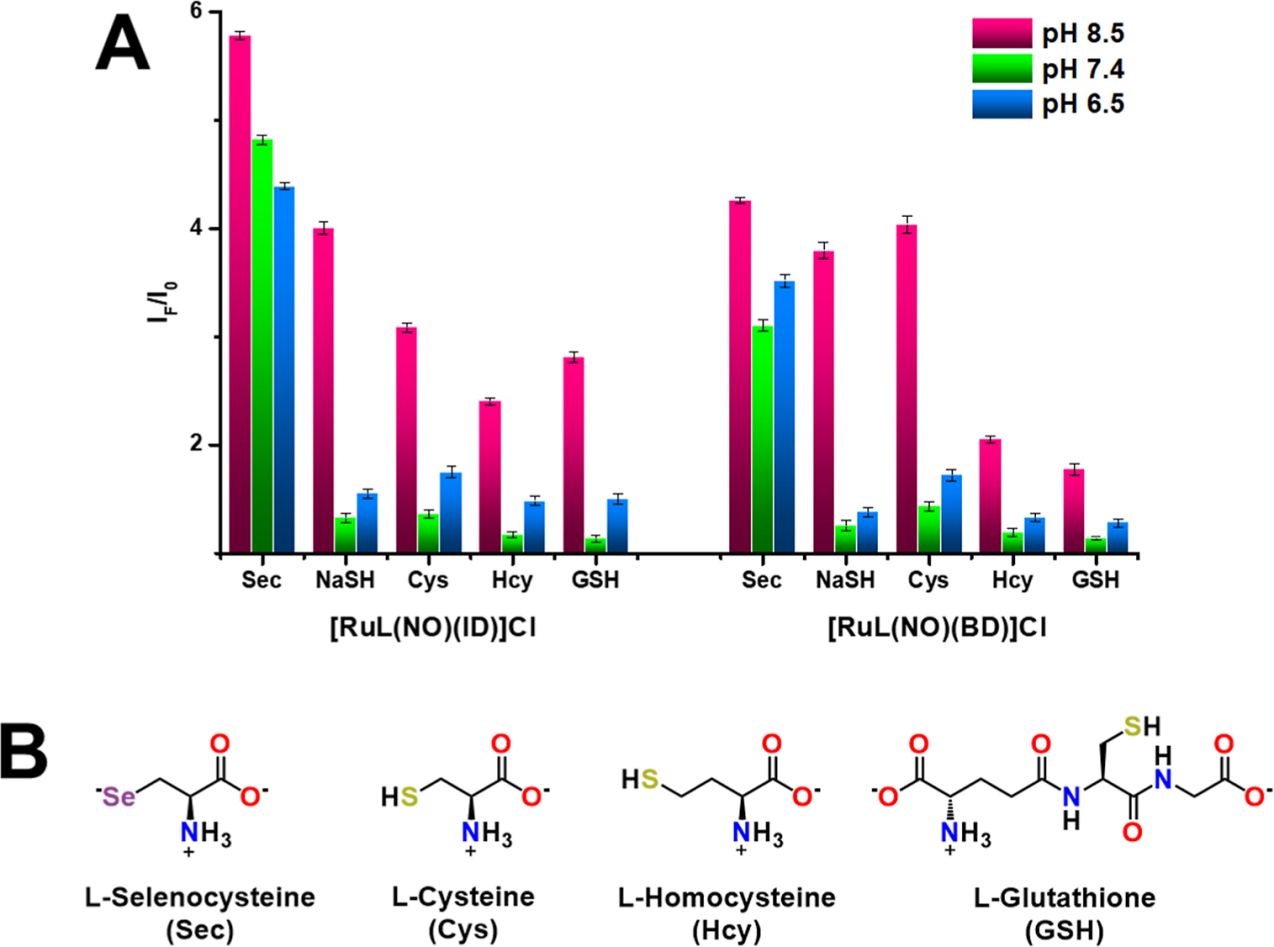
(A) Fluorescence enhancement at 502 nm of an aqueous solution (10 μM) of **[RuL(NO)(ID)]Cl** and **[RuL(NO)(BD)]Cl** upon additions of different bioanalytes (40 μM) at different pHs (MES 20 mM, pH 6.5; HEPES 20 mM, pH 7.4; TRIS 20 mM, pH 8.5), an average of triplicate experiments. (B) Bioanalytes used in this work.

As a first step, the relative fluorescence selectivity of the complex toward reductant nucleophilic bioanalytes was analyzed using cysteine (Cys), homocysteine (Hcy), glutathione (GSH), selenocysteine (Sec), and NaSH ([bioanalyte]_final_ = 40 μM) under different pH conditions (Figure 2). The bioanalytes were added to buffered aqueous solutions (MES 20 mM at pH 6.5; HEPES 20 mM at pH 7.4; TRIS 20 mM at pH 8.5) of chemodosimeters (10 μM) and the emission spectra were recorded (λ_ex_ = 340 nm). The fluorescence response of the chemodosimeters in the absence of any added bioanalyte (blank) was measured, and it was plotted against pH. Initially, the fluorescence intensity decreases slightly as the pH increases from 6.5 to 8.5. In this pH range, solutions of **[RuL(NO)(ID)]Cl** and **[RuL(NO)(BD)]Cl** exhibit weak fluorescence and broad emission bands with a maximum of around 502 nm. The emission quenching of Ru^II^-coordinated dansyl fluorophores (**ID** and **BD**) can be mainly attributed to energy transfer (ET) processes to metal-centered states.^97,98^ This possible ET arising from dansyl fluorophore to the Ru–NO^+^ almost completely quenches the fluorescent emission due to the strong electron-withdrawing effect of the coordinated NO^+^ ligand from the Ru^II^ center, which in turn draws electron density from the dansyl-imidazole (**ID**) or dansyl-benzimidazole (**BD**) directly bonded to it^99−101^ Therefore, the light energy absorbed by the fluorophores (**ID** and **BD**) is mainly transferred to the Ru–NO^+^ unit, and only a small portion is lost through fluorescence, resulting in weak fluorescent emission.^102,103^

**Figure 3:**
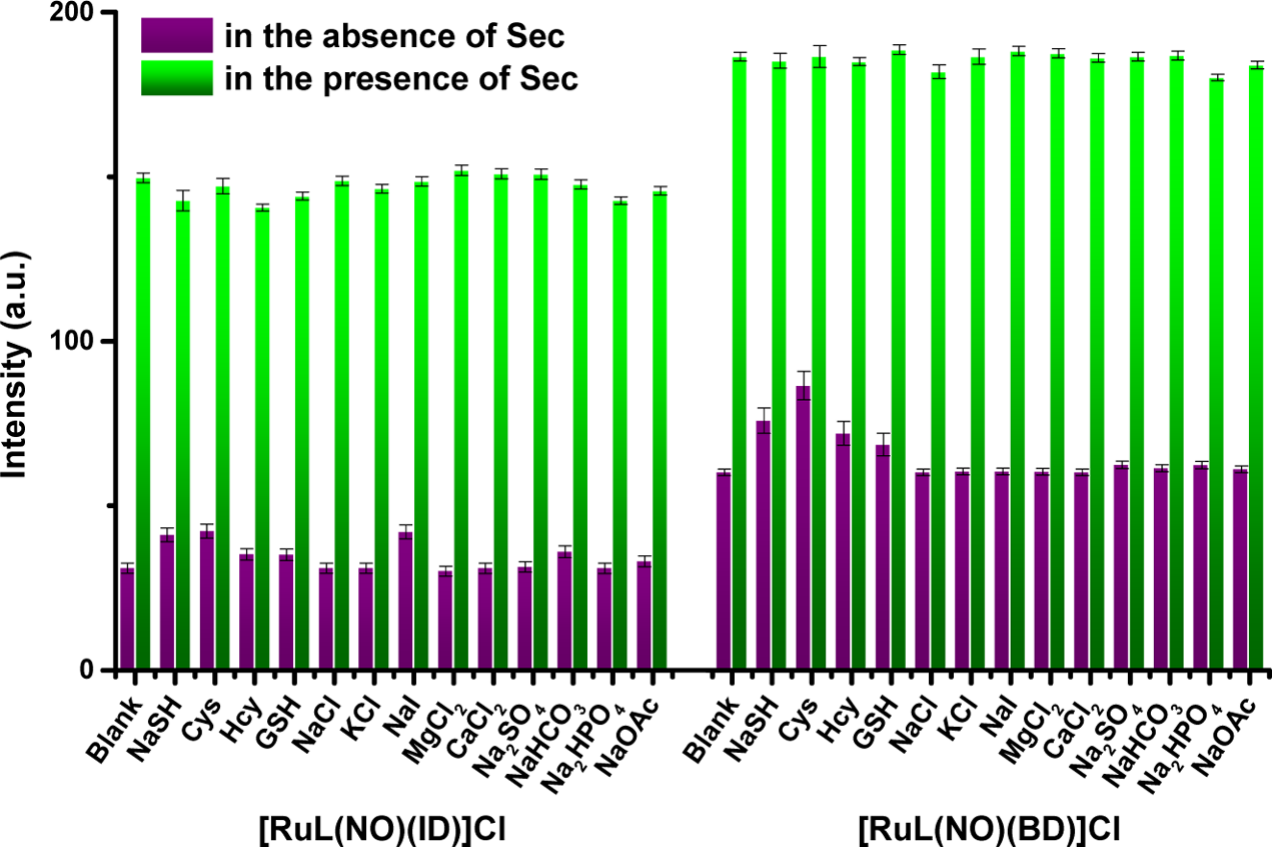
Fluorescence responses of **[RuL(NO)(ID)]Cl** (10 μM) and **[RuL(NO)(BD)]Cl** (10 μM) toward Sec (40 μM) in the presence (40 μM) of a background of several coexisting species at pH 7.4.

When different bioanalytes were added to buffered aqueous solutions (pH 6.5 and 7.4) of the chemodosimeters (10 μM), the fluorescent changes (*I*_F_/*I*_0_) of **[RuL(NO)(ID)]Cl** and **[RuL(NO)(BD)]Cl** modestly changed, which meant that bioanalytes (such as biothiols) did not react with the chemodosimeters. However, the fluorescence responses (*I*_F_/*I*_0_) of **[RuL(NO)(ID)]Cl** and **[RuL(NO)(BD)]Cl** were significantly enhanced when Sec (40.0 μM) was added, which meant that the chemodosimeters reacted with Sec to release NO^•^ at pH 6.5 and 7.4 (Figure 2A). This phenomenon also could be confirmed by the ^77^Se NMR, EPR, and IR analysis of **[RuL(NO)(ID)]Cl** and **[RuL(NO)(BD)]Cl** after treatment with Sec. Furthermore, when different relevant bioanalytes coexisted with Sec (NaCl, KCl, NaI, MgCl_2_, CaCl_2_, Na_2_SO_4_, NaHCO_3_, Na_2_HPO_4_, NaOAc, NaHS, Cys, Hcy, and GSH), there was no interference with the fluorescence intensity of the chemodosimeters at pH 7.4. Figure 3 shows that the background species does not affect the enhancement response at 502 nm induced by Sec (Figure S30). Since the p*K*_a_ of biothiols (∼8.4) is higher than that of Sec (∼5.24), they can react with the chemodosimeters only at elevated pH (∼8.5); as a result, biothiols hardly responded to **[RuL(NO)(ID)]Cl** and **[RuL(NO)(BD)]Cl** at pH ≤ 7.4 (Figure 2A). The above results demonstrated the ability of **[RuL(NO)(ID)]Cl** and **[RuL(NO)(BD)]Cl** to detect Sec with excellent sensitivity and selectivity at pH 7.4. The strong fluorescent change induced by Sec at pH 7.4 suggests that selenols have greater reactivity than their sulfur analogs toward {RuNO}^6^, which can be explained by the high degree of dissociation of Sec resulting in the predominant generation of the corresponding selenolate (p*K*_a_ ∼ 5.24), which can selectively react with **[RuL(NO)(ID)]Cl** and **[RuL(NO)(BD)]Cl** due to its better nucleophilicity. To verify this selective reactivity, we monitored the optical changes of **[RuL(NO)(ID)]Cl** and **[RuL(NO)(BD)]Cl** with some selenocysteine-related nucleophilic bioanalytes such as biothiols (Cys, Hcy, and GSH) and NaSH through fluorimetric and spectrophotometric titration experiments at pH 7.4 (Figures S16–S19).

**Figure 4:**
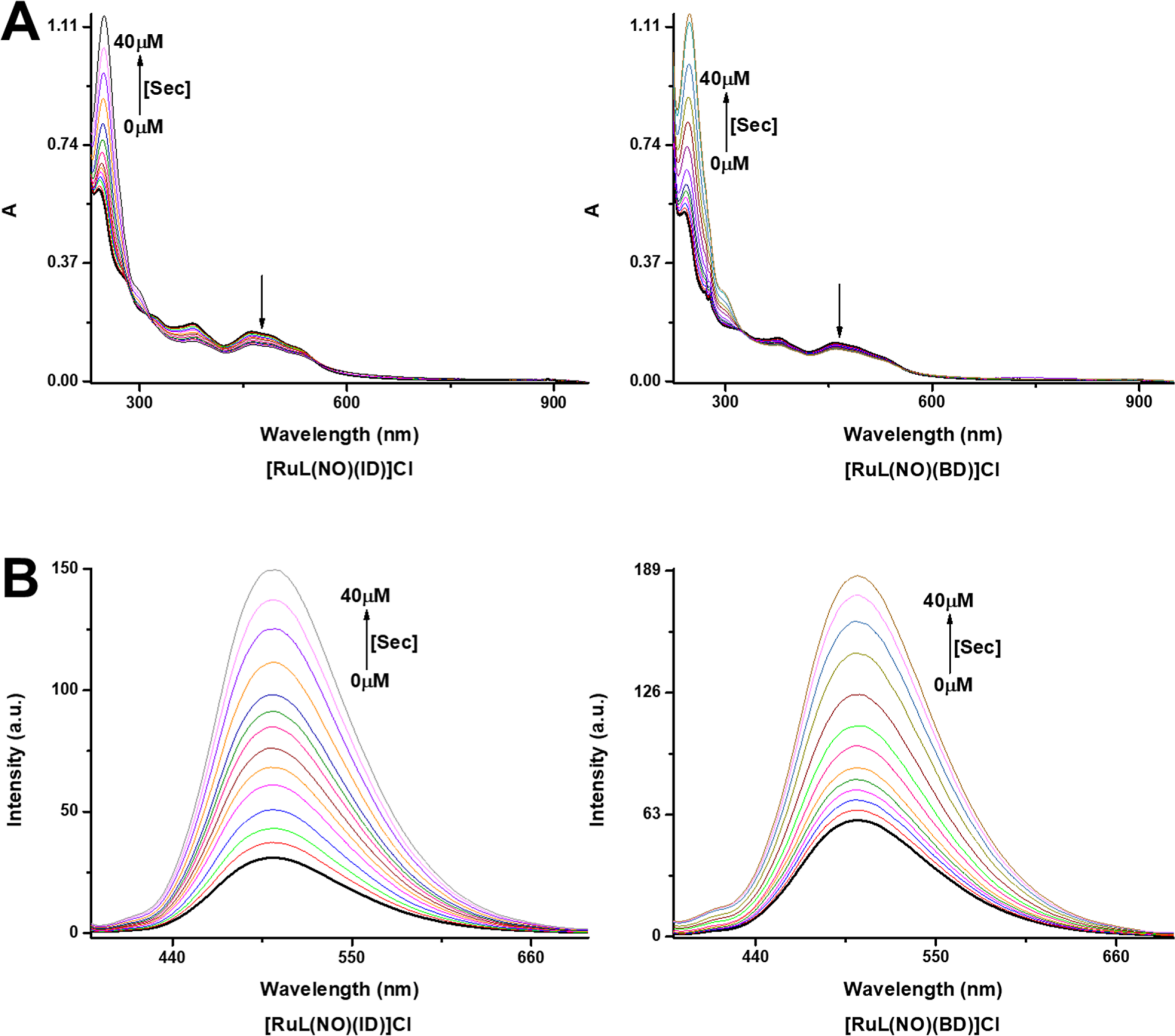
(A) UV/vis absorption spectra and (B) emission spectra (λ_ex_ = 340 nm) of **[RuL(NO)(ID)]Cl** and **[RuL(NO)(BD)]Cl** solutions (10 μM) upon the addition of increasing amounts of Sec at pH 7.4 (HEPES 20 mM, 25 °C).

Figure 4A shows the electronic absorption spectra obtained after the addition of aliquots of a Sec solution to solutions of **[RuL(NO)(ID)]Cl** or **[RuL(NO)(BD)]Cl** (10.0 μM) in a HEPES buffer (20 mM, pH 7.4). The complexes displayed strong absorption bands near 377 and 462 nm for **[RuL(NO)(ID)]Cl**, as well as 378 and 459 nm for **[RuL(NO)(BD)]Cl** that are largely attributed to the ligand-to-ligand charge transfer (LLCT) of π(naphophen and dansyl ligands) → π*(Ru–NO^+^) and metal-to-ligand charge transfer (MLCT) of dπ(Ru) → π*(Ru–NO^+^).^71,101,102,104,105^ However, when Sec was added progressively, these strong absorption bands above 315 nm gradually decreased as new bands centered at 250 and 300 nm were appreciated for both chemodosimeters. The isosbestic points at 310 and 556 nm for **[RuL(NO)(ID)]Cl**, as well as 323 and 551 nm for **[RuL(NO)(BD)]Cl,** are evidence of a chemical reaction. The final spectra of the aqua-complexes, **[RuL(OH**_**2**_**)(ID)]** and **[RuL(OH**_**2**_**)(BD)]**, correspond to the ruthenium-based products that release NO^•^.^58,59,106^ This phenomenon also could be confirmed by IR and MS (MALDI-TOF) analysis of **[RuL(NO)(ID)]Cl** and **[RuL(NO)(BD)]Cl** after treatment with 4.0 equiv of Sec.

The positive scan of MALDI-TOF in MeOH–H_2_O (2:1 v/v) showed practically one charged species at *m*/*z* = 1032.477 for **[RuL(OH**_**2**_**)(ID)]**, as well as 1046.141 for **[RuL(OH**_**2**_**)(BD)]**. These peaks were isotopically resolved and match very well the theoretical distribution for monocationic complexes {**[RuL(OH**_**2**_**)(ID)]** + K^+^ + 7H_2_O + CH_3_OH}^+^ (calcd. C_44_H_53_KN_5_O_13_RuS^+^, *m*/*z* 1032.477) and {**[RuL(OH**_**2**_**)(BD)]** + K^+^ + 5H_2_O + CH_3_OH}^+^ (calcd. C_48_H_51_KN_5_O_11_RuS^+^, *m*/*z* = 1046.141), corresponding to aqua-complexes (Figures S20–S23).

Furthermore, IR spectra of **[RuL(NO)(ID)]Cl** and **[RuL(NO)(BD)]Cl** were analyzed before and after adding Sec. After completing the reaction of **[RuL(NO)(ID)]Cl** or **[RuL(NO)(BD)]Cl** with 4.0 equiv of Sec in MeOH–H_2_O (2:1 v/v), the solvents were removed entirely, and then the collected powders were analyzed using IR spectroscopy. As found in Figure S24, the ν_NO_ bands were observed in both **[RuL(NO)(ID)]Cl** and **[RuL(NO)(BD)]Cl** and were absent in the reaction products, implying that NO^•^ was released from the original nitrosyl complexes.

On the basis of the experimental data and information reported in the literature, the changes in the electronic spectra for related systems show similarity to the reactions between {RuNO}^6^ and thiols.^58,59,106^ Thus, a nucleophilic attack on the nitrogen by a selenol was anticipated, as shown in the reaction illustrated in Scheme 2. Because of the π-extended conjugated systems of the equatorial tetradentate ligand **L** and the modest trans effect and trans influence of the **ID** and **BD** ligands, the dissociation of NO^+^ ligand from the chemodosimeters (in which the Ru^II^–NO^+^ back-bonding is weakened) may be faster because both ligands are contributing to the bathochromic shift of the dπ(Ru) → π*(Ru–NO^+^) MLCT band, promoting that the reduction potential of Ru^II^(NO^+^)/Ru^II^(NO^0^) can be more positive and the nitrosyl ligand in these compounds would be sufficiently active to undergo nucleophilic attack by Sec even at pH ≤ 7.4. On the other hand, when biothiols such as NaSH, GSH, Cys, and Hcy were progressively added to both chemodosimeter solutions under the same conditions, a minor change was observed in all absorption bands (Figures S16–S19).

**Figure 5:**
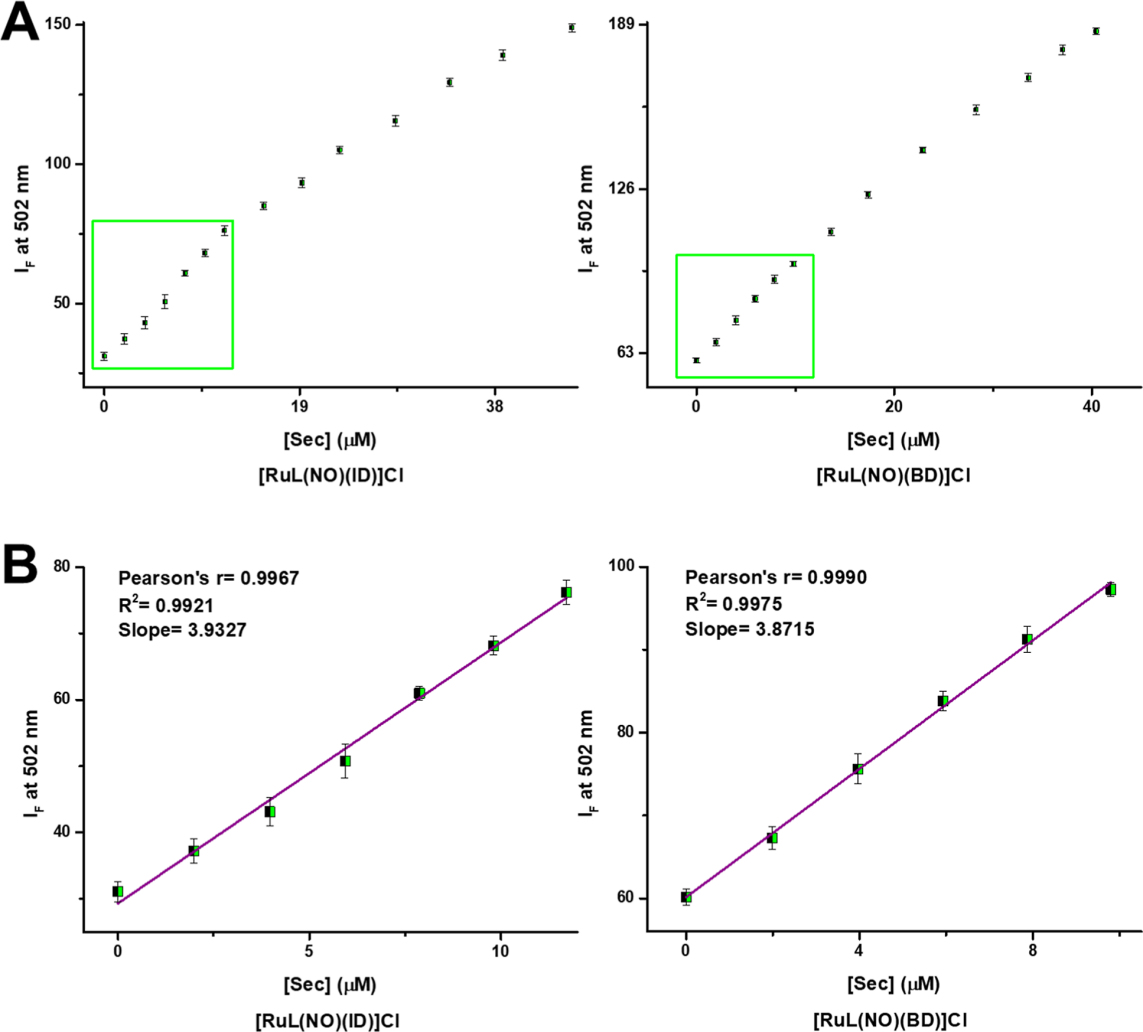
(A) Emission profiles upon addition of Sec at pH 7.4 for **[RuL(NO)(ID)]Cl** and **[RuL(NO)(BD)]Cl** solutions (10 μM). The curves were drawn with *I*_F_ versus [Sec] (0–40.0 μM). (B) Linear relationship between Sec and *I*_F_ at 502 nm (*I*_F_ = s[Sec] + 29.304 for **[RuL(NO)(ID)]Cl** and *I*_F_ = s[Sec] + 60.160 **[RuL(NO)(BD)]Cl**; where s = slope).

Figure 4B shows the fluorimetric titrations of **[RuL(NO)(ID)]Cl** and **[RuL(NO)(BD)]Cl** with Sec. The chemodosimeters showed weak green fluorescence and exhibited an emission band at 502 nm, ascribed to the fluorescence signal from dansyl ligands directly coordinated to the Ru^II^ centers. However, upon the addition of increasing concentrations of Sec, the emission intensities of **[RuL(NO)(ID)]Cl** and **[RuL(NO)(BD)]Cl** at 502 nm gradually increased. The *I*_F_ at 502 nm enhanced sharply from 31.06 to 149.68 (∼5 folds) for **[RuL(NO)(ID)]Cl**, as well as from 60.17 to 186.51 (∼3 folds) for **[RuL(NO)(BD)]Cl** after being reacted with Sec. Notably, there are linear dependencies in the *I*_F_ for **[RuL(NO)(ID)]Cl** (range of [Sec] = 0–12 μM, *R*^2^ = 0.9921) and **[RuL(NO)(BD)]Cl** (range of [Sec] = 0–10 μM, *R*^2^ = 0.9975) with Sec at pH 7.4 (Figure 5). The detection limits (LOD) for Sec were calculated to be 0.31 μM for **[RuL(NO)(ID)]Cl** and 0.12 μM for **[RuL(NO)(BD)]Cl** (LOD = 3σ/*s*, where σ = standard deviation of blank luminescence intensity and *s* = slope of the calibration plot), which are lower than the concentration level of Sec (0.5 μM) in healthy people.^25,107^

The fluorimetric titrations of the chemodosimeters with the addition of Hcy and GSH gave exceptionally low responses (Figures S18 and S19). The addition of Cys and HS^–^ resulted in a modest increase in emission intensity, but it was still significantly lower than that observed for Sec (Figures S16 and S17).

**Figure 6:**
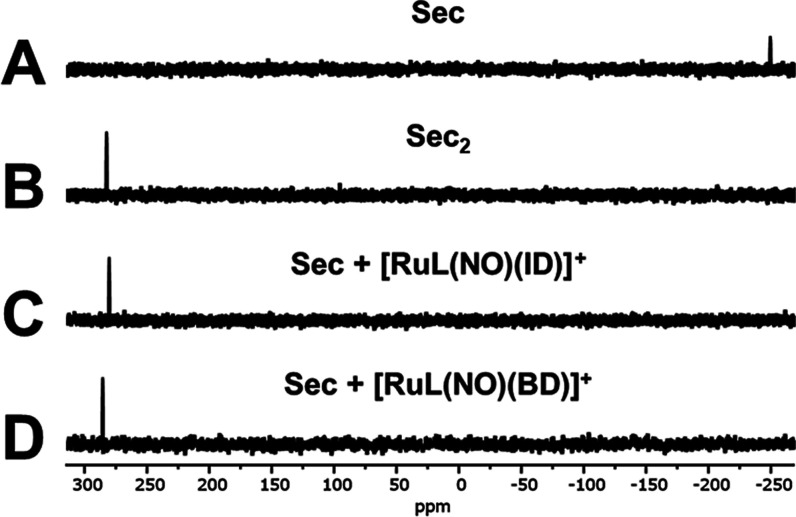
^77^Se NMR (57 MHz) spectra of (A) Sec, (B) Sec_2_, and (C) Sec in the presence of 4.0 equiv of **[RuL(NO)(ID)]**^**+**^ or (D) **[RuL(NO)(BD)]**^**+**^ in DMSO–D_2_O (1:5 v/v) at pD 7.4 (200 mM PBS).

Further evidence of the high reactivity of Sec for **[RuL(NO)(ID)]Cl** and **[RuL(NO)(BD)]Cl** was obtained by ^77^Se NMR measurements (Figure 6). The free Sec (10.0 mM) has a signal at δ – 252.3 ppm, and the addition of 4.0 equiv of **[RuL(NO)(ID)]Cl** or **[RuL(NO)(BD)]Cl** exhibits a new signal at δ 281.5 and 283.2 ppm, respectively. The signals at δ 281.5 and 283.2 ppm indicate the formation of Sec_2_, suggesting the oxidation of Sec upon reaction with **[RuL(NO)(ID)]Cl** or **[RuL(NO)(BD)]Cl** (Scheme 2). Reference chemical shift for Sec_2_ formation in the Sec reaction with the chemodosimeters agrees with the free Sec_2_ (10.0 mM) spectrum (δ 287.9; Figure 6B).^108−110^

**Figure 7:**
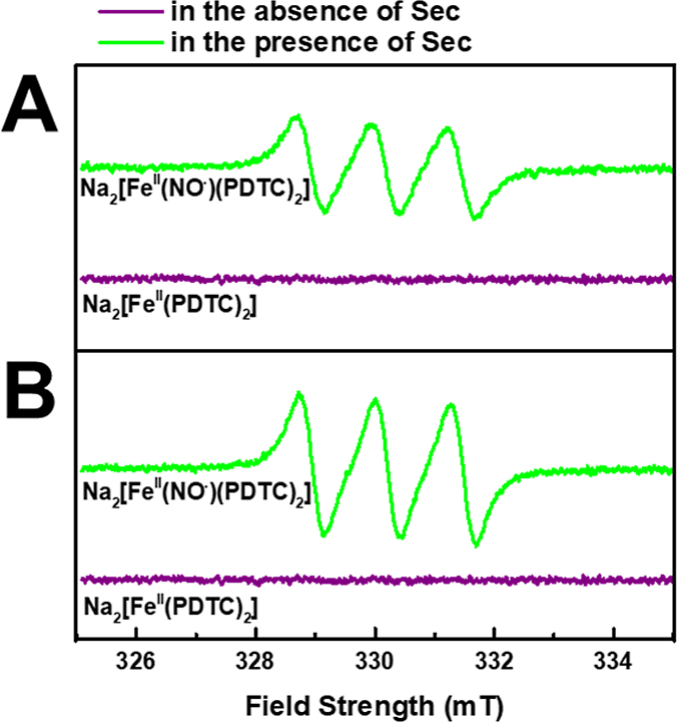
Room-temperature EPR spectra obtained from the mixture solutions of (A) **[RuL(NO)(ID)][Cl]** + Na_2_[Fe^II^(PDTC)_2_] and (B) **[RuL(NO)(BD)][Cl]** + Na_2_[Fe^II^(PDTC)_2_] before and after treatment with 4.0 equiv of Sec.

From the above data, which indicated that the dissociation of the diamagnetic Ru(II)–NO^+^ bonds of **[RuL(NO)(ID)]Cl** and **[RuL(NO)(BD)]Cl** produced the corresponding diamagnetic aqua-complexes, it is expected that NO^•^ was released by the nucleophilic attack of Sec. Disodium l-proline-dithiocarbamato-iron(II), Na_2_[Fe^II^(PDTC)_2_], has been known to trap NO^•^ to become an EPR-detectable spin adduct both in vivo and in vitro.^72−74^Figure 7 shows the EPR signals of such adducts produced in the mixture solutions of **[RuL(NO)(ID)]Cl** + Na_2_[Fe^II^(PDTC)_2_] and **[RuL(NO)(BD)]Cl** + Na_2_[Fe^II^(PDTC)_2_] before and after treatment with 4.0 equiv of Sec. The results show that before the treatment with Sec, no EPR signals were observed; however, after the reaction with Sec, weak triplet EPR signals were observed. The observed ^14^N nuclear hyperfine coupling constant (*A*_N_) of 12.90 G and the isotropic *g*-value (*g*_iso_) of 2.042 are in agreement with the previously reported *g*-values of the Na_2_[Fe^II^(PDTC)_2_–NO^•^] adducts.^111,112^

### Electronic Density Calculations

**Figure 8:**
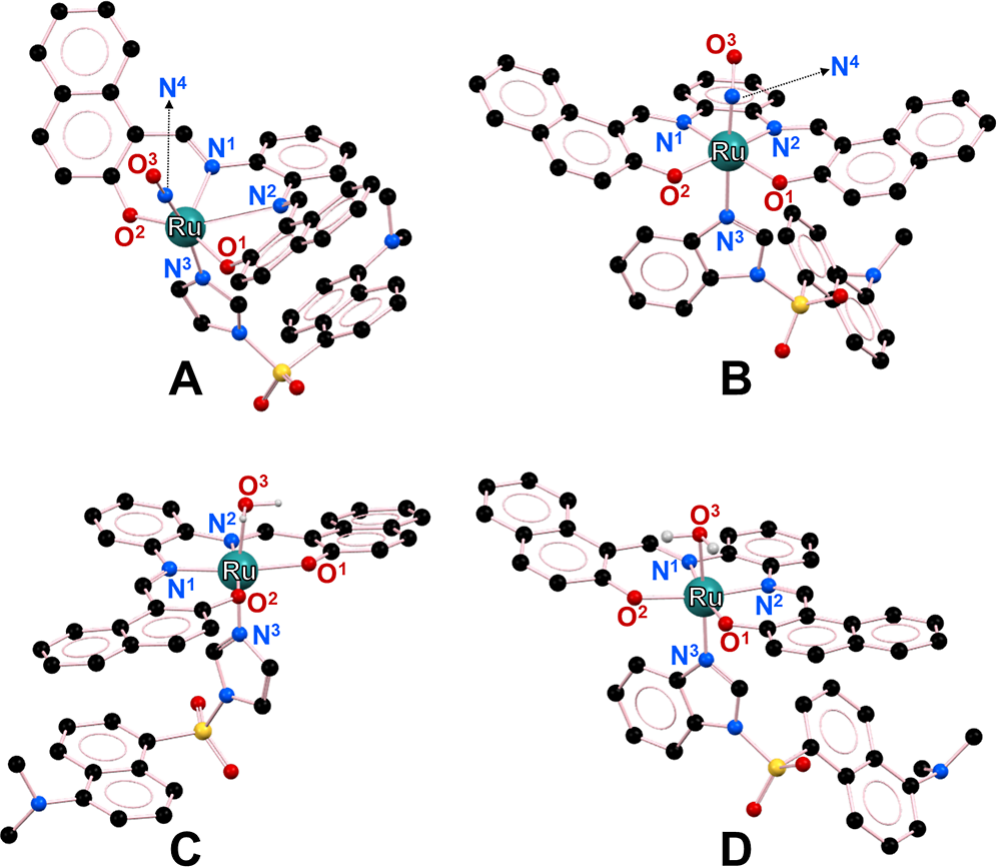
Minimal energy geometries for (A) **[RuL(NO)(ID)]**^**+**^, (B) **[RuL(NO)(BD)]**^**+**^, (C) **[RuL(OH**_**2**_**)(ID)]**, and (D) **[RuL(OH**_**2**_**)(BD)]** at the B3LYP/DGDZVP basis set.

To better understand the chemodosimetric mechanism of the {RuNO}^6^ complexes toward Sec, theoretical calculations were performed by DFT, using the Gaussian 09^113,114^ and the B3LYP functional^115−118^ with the established basis 6-31G**^119,120^ for the atoms (C, H, N, S, O, and Se) and LANL2DZ for Ru^II^.^121^ The optimization calculations first began with functionals and small bases (HF/3-21g) to stabilize the energies of complexes with too large a molecular structure. The data obtained were used as input for optimizing the chemodosimeters and their respective aqua-complexes using B3LYP in DGDZVP as the basis set. This study provides important features to understand the reactivity of Sec toward the nitrosonium ligand (NO^+^) of ruthenium nitrosyls that promote the release of NO^•^ and the respective aquation of the chemodosimeters (**[RuL(NO)(ID)]**^**+**^ and **[RuL(NO)(BD)]**^**+**^). Figure 8 shows the optimized geometries for both chemodosimeters. The structures of complexes **[RuL(NO)(ID)]**^**+**^ and **[RuL(NO)(BD)]**^**+**^ show that the Ru^II^ ion is located in a distorted octahedral geometry forming an equatorial plane with the tetradentate ligand (**LH**_**2**_) in the deprotonated form (**L** = naphophen^2–^) for both complexes. The axial positions are occupied by the NO^+^ ligand and the respective dansyl ligands (**ID** and **BD**). Additionally, the optimization for **[RuL(OH**_**2**_**)(ID)]** and **[RuL(OH**_**2**_**)(BD)]** shows geometrical parameters similar to their respective nitrosyl complexes, although with less octahedral distortion in the equatorial plane (Figure 8). The geometrical data (bond distances and/or bond angles) are presented in the Supporting Information (Table S3), which is consistent with the published reports.^122,123^

After analyzing geometrical data for the chemodosimeters, the equatorial plane formed by N^1^, N^2^, O^1^, and O^2^ indicates distances of 2.415 (Ru–N^1^), 2.878 (Ru–N^2^), 2.096 (Ru–O^1^), and 2.092 (Ru–O^2^) Å for **[RuL(NO)(ID)]**^**+**^ and 2.031 (Ru–N^1^), 2.030 (Ru–N^2^), and 2.093 (Ru–O,^1^ Ru–O^2^) Å for **[RuL(NO)(BD)]**^**+**^. The axial distances are 1.754 (Ru–NO^+^) and 2.158 (Ru–N^3^) Å for **[RuL(NO)(ID)]**^**+**^ and 1.879 (Ru–NO^+^) and 2.240 (Ru–N^3^) Å for **[RuL(NO)(BD)]**^**+**^. However, when the chemodosimeters were aquated, yielding **[RuL(OH**_**2**_**)(ID)]** and **[RuL(OH**_**2**_**)(ID)]** (where H_2_O replaced NO^+^ trans to **ID** or **BD** in the axial position), some bond distances around Ru^II^ (equatorial plane) decreased significantly. The equatorial distances are 2.021 (Ru–N^1^), 2.020 (Ru–N^2^), 2.101 (Ru–O^1^), and 2.100 (Ru–O^2^) Å for **[RuL(OH**_**2**_**)(ID)]** and 2.021 (Ru–N^1^), 2.030 (Ru–N^2^), 2.100 (Ru–O^1^), and 2.109 (Ru–O^2^) Å for **[RuL(OH**_**2**_**)(BD)]**. Furthermore, the results show that the aqua-complexes present a radical increase in the axial bond distance (Ru–OH_2_) and a concomitant decrease in the axial bond distance between Ru^II^ and the dansyl ligand (Ru–N^3^) relative to their nitrosyl complexes. The axial distances are 2.250 (Ru–OH_2_) and 2.055 (Ru–N^3^) Å for **[RuL(OH**_**2**_**)(ID)]** and 2.242 (Ru–OH_2_) and 2.090 (Ru–N^3^) Å for **[RuL(OH**_**2**_**)(BD)]** (Schemes S2 and S3). Perhaps the decrease in the overall hardness of the complexes and the trans influence of the dansyl ligand (**ID** or **BD**) on the aqua ligand are expected to play crucial roles in fluorescence enhancement. It suggests that the addition of Sec to the chemodosimeters weakens the Ru–NO^+^ bond due to a nucleophilic attack promoted by Sec to the nitrosonium groups (NO^+^) of **[RuL(NO)(ID)]**^**+**^ and **[RuL(NO)(BD)]**^**+**^, which undergo aquation reactions, accompanied by the release of NO^•^. This dissociation of the NO^+^ ligand decreases with the hardness of Ru^II^, and as a consequence, the softness of the respective aqua species (**[RuL(OH**_**2**_**)(ID)]** and **[RuL(OH**_**2**_**)(ID)]**) is obviously increased; thus, ultimately, it impacts the fluorescence intensity of dansyl ligands, agreeing with the experimental findings. In addition, due to the loss of the strong electron-withdrawing effect of the coordinated NO^+^ ligand from the Ru^II^ center, upon release of NO^•^ caused by the nucleophilic attack of Sec, the aqua-complex products, **[RuL(OH**_**2**_**)(ID)]** and **[RuL(OH**_**2**_**)(BD)]**, cause enhancement of dansyl fluorophore. Therefore, the light energy absorbed by the fluorophores (**ID** and **BD**) is mostly not transferred to the Ru^II^–OH_2_ unit, and a small portion is lost via a nonradiative mechanism, resulting in strong fluorescent emission (Figures S25–S28).

**Figure 9:**
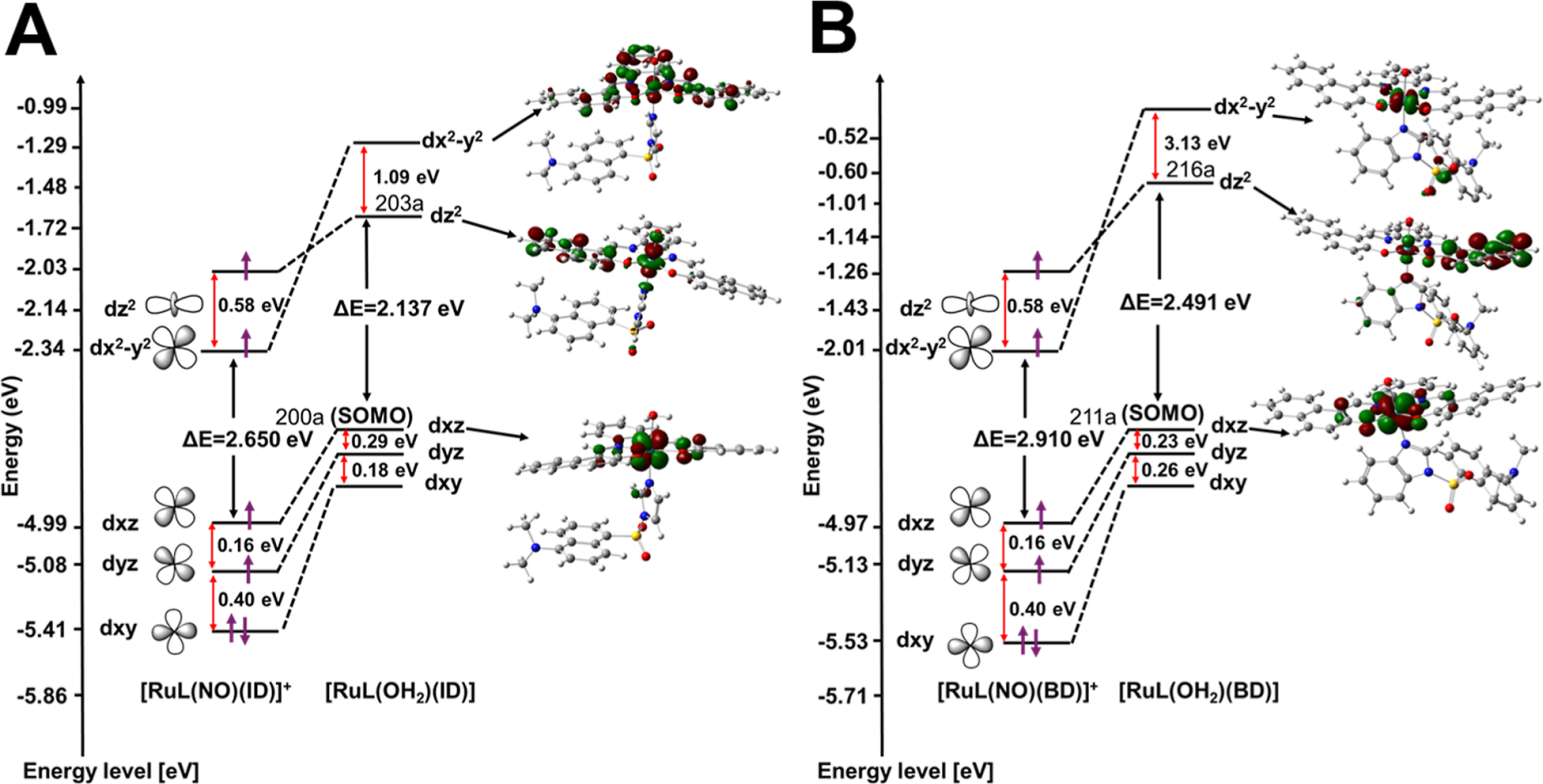
Comparative molecular orbital analysis of (A) **[RuL(NO)(ID)]**^**+**^ with **[RuL(OH**_**2**_**)(ID)]** and (B) **[RuL(NO)(BD)]**^**+**^ with **[RuL(OH**_**2**_**)(BD)]**.

Besides, to prove the binding of NO^+^ to **[RuL(NO)(ID)]**^**+**^ and **[RuL(NO)(BD)]**^**+**^ complexes and their substitution by water (**[Ru(OH**_**2**_**)(BD)]** and **[RuL(OH**_**2**_**)(ID)]**), the MO analysis was carried out using the B3LYP functional with the DGDZVP base. The resulting MOs are HOMO to HOMO – *X* (*X* = 0–9 and 12–23) for **[RuL(NO)(BD)]**^**+**^, HOMO to HOMO – *X* (*X* = 0–7, 9–11, and 14–15) for **[RuL(NO)(ID)]**^**+**^, HOMO to HOMO – *X* (*X* = 0–7) for **[RuL(OH**_**2**_**)(BD)]**, and HOMO to HOMO – *X* (*X* = 0–5, 7, and 8) for **[RuL(OH**_**2**_**)(ID)]** showing that there is a strong overlap between dansyl ligands (**ID** and **BD**) and the Ru^II^, upon release of NO^•^ and coordination of water. In the case of nitrosyl complexes, a significant interaction of the *d*(Ru^II^) orbital with π/p of the ligands was observed, which is consistent with the geometrical parameters (a decrease in the bond length for Ru^II^–NO^+^ and an increase in the bond length for Ru^II^–OH_2_), aiding NO^+^ ligand scission in both complexes (Figures 9, S25–S29). This agrees with the d*x*^2^ – *y*^2^ orbital energy (−2.14 and −2.01 eV) resulted for **[RuL(NO)(ID)]**^**+**^ and **[RuL(NO)(BD)]**^**+**^, respectively, which are considerably higher than that resulted for **[RuL(OH**_**2**_**)(ID)]** (−1.24 eV) or **[Ru(OH**_**2**_**)(BD)]** (−1.32 eV). The HOMO–LUMO band gap energies for **[RuL(NO)(ID)]**^**+**^ (2.650 eV) and **[RuL(NO)(BD)]**^**+**^ (2.910) have been decreased to 2.137 eV for **[RuL(OH**_**2**_**)(ID)]** and 2.491 for **[Ru(OH**_**2**_**)(BD)]** (Figure 9). The excitation of the metal d electrons to π* and σ* in Ru^II^–NO^+^ is also associated with NO^•^ release and the addition of the water molecule (Ru^II^–OH_2_). The labialization can be via two pathways: (a) the direct excitation of an electron (from a bonding dπ–π* orbital) to an antibonding π*–dπ orbital; and/or (b) indirectly, the excitation of metal-to-ligand charge–transfer transitions (MLCT); then undergoing a relaxation in the excited state to release the NO^•^.

### Cell Imaging

**Figure 10:**
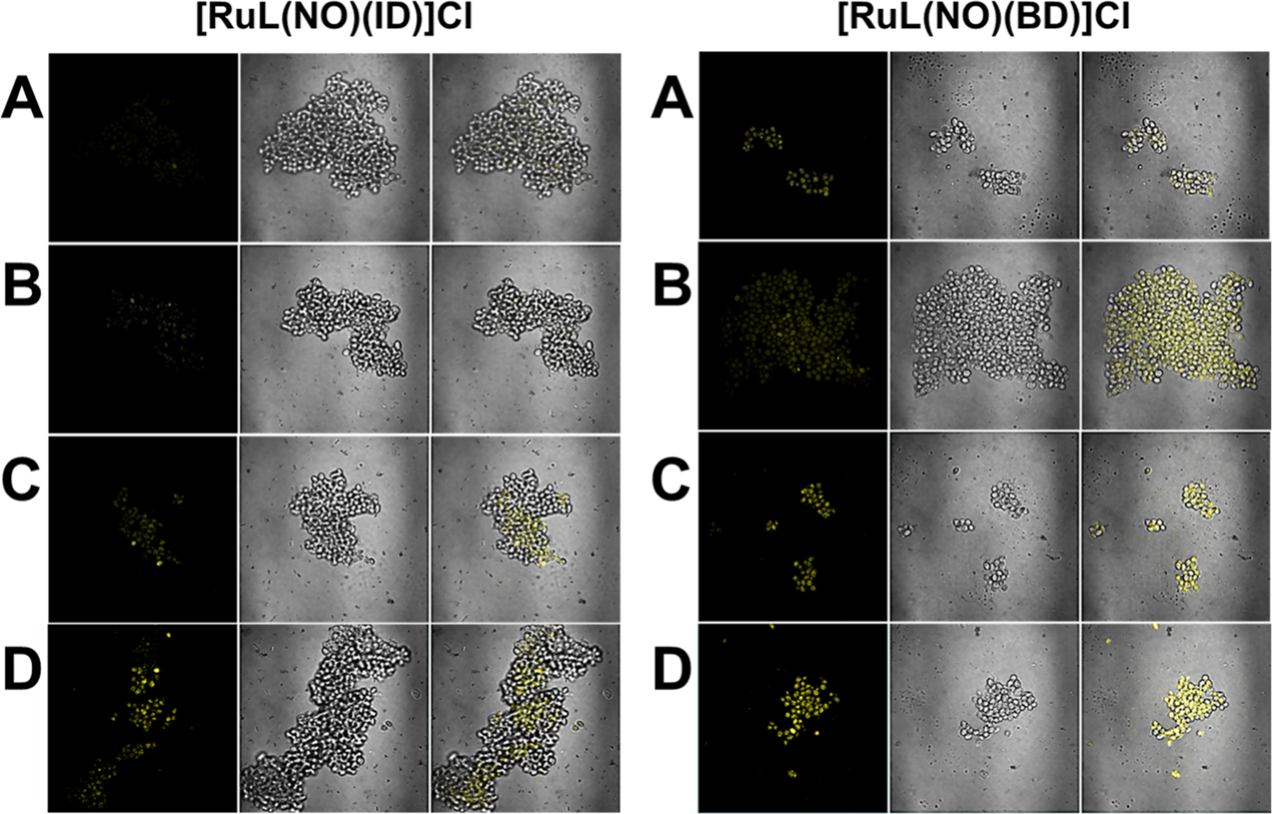
Confocal fluorescence microscopy images of the SCC after incubation with **[RuL(NO)(ID)]Cl** and **[RuL(NO)(BD)]Cl** (10 μM; λ_ex_ = 405 nm): (A) Control SCC, (B) SCC with [Sec] = 10 μM, (C) SCC with [Sec] = 20 μM, (D) SCC with [Sec] = 40 μM.

The cell imaging capability of chemodosimeters toward Sec in live *S. cerevisiae* cells (SCC) was studied; briefly, yeast cells were first grown in MHB and were incubated with the corresponding chemodosimeter (10 μM). The experiments have been divided into four groups. The first one was chosen as a control, and the other three groups were pretreated with Sec 10.0, 20.0, and 40.0 μM, respectively. Each group of cells was observed by a confocal fluorescence microscope “Olympus FV1000 instrument, equipped with a diode laser (405 nm)”. The cell size and growth rate in culture remained the same, implying that there was no toxicity from the chemodosimeters at 10 μM to SCC. The chemodosimeters initially displayed an insignificant yellowish green fluorescence. However, with the incremental addition of Sec from 0 to 40 μM, the yellowish green fluorescence exhibited a substantial enhancement, as illustrated in Figure 10. The images illustrated in Figure 10 prove that **[RuL(NO)(ID)]Cl** and **[RuL(NO)(BD)]Cl** can be used to detect Sec in living cells such as yeast cells (SCC).

## Conclusions

In summary, a novel fluorescent chemodosimetric mechanism has been developed for the selective detection of Sec by {RuNO}^6^ complexes in aqueous media at pH 7.4, which are constituted by a tetradentate ligand carrying a π-extended system **L** and a monodentate ligand derived from the conjugated dansyl group that acts as a strong fluorescent signaling unit (**ID** and **BD**) when Sec reacts with {RuNO}^6^, enhancing fluorescence and releasing NO^•^. The chemodosimetric mechanism resides in the reaction between the selenolate present in Sec (R–SeH, p*K*_a_ = ∼5.24) and the electrophilic nitrosyl ligands **[RuL(NO)(ID)]**^**+**^ and **[RuL(NO)(BD)]**^**+**^. Both NO^•^ and Selenocystine (Sec_2_), as well as the respective fluorescent aqua-complexes of Ru^II^, **[RuL(OH**_**2**_**)(ID)]** and **[RuL(OH**_**2**_**)(BD)],** are the proposed products of a redox reaction, according to fluorimetric titrations, UV–vis titrations, ^77^Se NMR, and electronic density calculations. All the experiments performed above indicate that **[RuL(NO)(ID)]**^**+**^ and **[RuL(NO)(BD)]**^**+**^ own fast turn-on fluorescence responses (5 min), quantitative determinations in a range of [Sec] = 0–12 μM for **[RuL(NO)(ID)]Cl** and 0–10 μM for **[RuL(NO)(BD)]Cl**, with high sensitivities (LOD = 0.31 μM for **[RuL(NO)(ID)]Cl** and 0.12 μM for **[RuL(NO)(BD)]Cl**), selectivity toward Sec over biothiols, and representative bioanalytes such as oxyanions, halides, and cationic electrolytes. The LODs are lower than the concentration level of Sec (0.5 μM) in healthy people. In addition, confocal microscopic fluorescence images of yeast cells with **[RuL(NO)(ID)]**^**+**^ and **[RuL(NO)(BD)]**^**+**^ revealed that there were green distinctive emissions in the presence of Sec. Finally, the chemodosimeters **[RuL(NO)(ID)]**^**+**^ and **[RuL(NO)(BD)]**^**+**^ could be successfully applied in tracking Sec in living cells, and consequently, **[RuL(NO)(ID)]Cl** and **[RuL(NO)(BD)]Cl** can be used as a suitable platform for the design of new fluorescent chemodosimeters, chemosensors, and probes.

## Supporting Information Available

General synthesis of all ligands and complexes; molecular structures, bond distances, and orbital energies; ^1^H NMR (300 MHz), IR (ATR), and ^13^C NMR (75 MHz) spectra; positive scan MS MALDI-TOF spectrum; partial positive scan MS MALDI TOF spectrum; UV/Vis absorption spectra and emission spectra; IR spectra; molecular orbital at the gaseous state; frontier molecular orbital energy level diagram at the gaseous state; visualization of the electron density isosurface determined by B3LYP/DGDZVP, HOMO and LUMO contour plots; fluorescence enhancement; crystal data and structure refinement; and bond lengths and bond angles (PDF)

## References

[ref1] Valand R. S.; Sivaiah A. Recent Progress in the Development of Small-Molecule Fluorescent Probes for Detection and Imaging of Selenocysteine and Application in Thyroid Disease Diagnosis. J. Mater. Chem. B 2023, 11 (12), 2614–2630. 10.1039/D3TB00035D. 36877143

[ref2] Liu Y.; Feng X.; Yu Y.; Zhao Q.; Tang C.; Zhang J. A Review of Bioselenol-Specific Fluorescent Probes: Synthesis, Properties, and Imaging Applications. Anal. Chim. Acta 2020, 1110, 141–150. 10.1016/j.aca.2020.03.027. 32278389

[ref3] Zhang J.; Zhan Y.; Li-Hu Y.; Qi Y.; Wang R.; Meng L. Recent Progress in Fluorescent Chemosensors for Selenium Compounds. Chinese J. Org. Chem. 2020, 40 (7), 1847 10.6023/cjoc202002025.

[ref4] Huang Y.; Song B.; Chen K.; Tang Z.; Ma H.; Kong D.; Liu Q.; Yuan J. Mitochondria-Targetable Ratiometric Time-Gated Luminescence Probe Activated by Selenocysteine for the Visual Monitoring of Liver Injuries. Anal. Chem. 2023, 95 (8), 4024–4032. 10.1021/acs.analchem.2c04409. 36799513

[ref5] Luo K.; Jia M.; Xie C.; Yang Q.; Tan L.; Liu X.; Zhou L. A Unique NIR Dye Constructed Mitochondrial Anchoring Fluorescent Probe for Highly Selective Selenocysteine Detection and Imaging in Living Cells and Mice. Sens Actuators B Chem. 2023, 375, 132944 10.1016/j.snb.2022.132944.

[ref6] Yang Q.; Xie C.; Luo K.; Tan L.; Peng L.; Zhou L. Rational Construction of a New Water Soluble Turn-on Colorimetric and NIR Fluorescent Sensor for High Selective Sec Detection in Se-Enriched Foods and Biosystems. Food Chem. 2022, 394, 133474 10.1016/j.foodchem.2022.133474. 35716503

[ref7] Wang Z.; Su W.; Zheng H.; Yang S.; Yang T.; Han T.; Dessie W.; He X.; Jiang Y.; Hao Y. Two Phenanthroimidazole Turn-on Probes for the Rapid Detection of Selenocysteine and Its Application in Living Cells Imaging. Spectrochim. Acta, Part A 2022, 267, 120585 10.1016/j.saa.2021.120585. 34782266

[ref8] Liu Y.; Feng X.; Meng Q.; Zhu J.; Jia X.; Zhao Q.; Tang C.; Yu Y.; Zhang J. A Naphthimide Fluorescent Probe for the Detection of Selenols in Selenium-Enriched Tan Sheep. Food Chem. 2022, 373, 131647 10.1016/j.foodchem.2021.131647. 34838402

[ref9] Mousa R.; Notis Dardashti R.; Metanis N. Selenium and Selenocysteine in Protein Chemistry. Angew. Chem., Int. Ed. 2017, 56 (50), 15818–15827. 10.1002/anie.201706876. 28857389

[ref10] Zhang B.; Zhang J.; Peng S.; Liu R.; Li X.; Hou Y.; Han X.; Fang J. Thioredoxin Reductase Inhibitors: A Patent Review. Expert Opin. Ther. Pat. 2017, 27 (5), 547–556. 10.1080/13543776.2017.1272576. 27977313

[ref11] Zhang J.; Li X.; Han X.; Liu R.; Fang J. Targeting the Thioredoxin System for Cancer Therapy. Trends Pharmacol. Sci. 2017, 38 (9), 794–808. 10.1016/j.tips.2017.06.001. 28648527

[ref12] Köhrle J.; Gärtner R. Selenium and Thyroid. Best Pract. Res., Clin. Endocrinol. Metab. 2009, 23 (6), 815–827. 10.1016/j.beem.2009.08.002. 19942156

[ref13] Bhattacharya P. T.; Misra S. R.; Hussain M. Nutritional Aspects of Essential Trace Elements in Oral Health and Disease: An Extensive Review. Scientifica 2016, 2016, 1–12. 10.1155/2016/5464373. PMC4940574 27433374

[ref14] Hatfield D. L.; Tsuji P. A.; Carlson B. A.; Gladyshev V. N. Selenium and Selenocysteine: Roles in Cancer, Health, and Development. Trends Biochem. Sci. 2014, 39 (3), 112–120. 10.1016/j.tibs.2013.12.007. 24485058 PMC3943681

[ref15] Garbo S.; Di Giacomo S.; Łażewska D.; Honkisz-Orzechowska E.; Di Sotto A.; Fioravanti R.; Zwergel C.; Battistelli C. Selenium-Containing Agents Acting on Cancer—A New Hope?. Pharmaceutics 2023, 15 (1), 104 10.3390/pharmaceutics15010104. PMC9860877 36678733

[ref16] Vinceti M.; Filippini T.; Wise L. A. Environmental Selenium and Human Health: An Update. Curr. Environ. Health Rep. 2018, 5 (4), 464–485. 10.1007/s40572-018-0213-0. 30280317

[ref17] Schomburg L. The Other View: The Trace Element Selenium as a Micronutrient in Thyroid Disease, Diabetes, and Beyond. Hormones 2020, 19 (1), 15–24. 10.1007/s42000-019-00150-4. 31823341

[ref18] Kaur K.; Saini R.; Kumar A.; Luxami V.; Kaur N.; Singh P.; Kumar S. Chemodosimeters: An Approach for Detection and Estimation of Biologically and Medically Relevant Metal Ions, Anions and Thiols. Coord. Chem. Rev. 2012, 256 (17–18), 1992–2028. 10.1016/j.ccr.2012.04.013.

[ref19] Ding S.; Liu M.; Hong Y. Biothiol-Specific Fluorescent Probes with Aggregation-Induced Emission Characteristics. Sci. China Chem. 2018, 61 (8), 882–891. 10.1007/s11426-018-9300-5.

[ref20] Chen X.; Zhou Y.; Peng X.; Yoon J. Fluorescent and Colorimetric Probes for Detection of Thiols. Chem. Soc. Rev. 2010, 39 (6), 2120 10.1039/b925092a. 20502801

[ref21] Areti S.; Verma S. K.; Bellare J.; Rao C. P. Selenocysteine vs Cysteine: Tuning the Derivatization on Benzenesulfonyl Moiety of a Triazole Linked Dansyl Connected Glycoconjugate for Selective Recognition of Selenocysteine and the Applicability of the Conjugate in Buffer, in Serum, on Silica Gel, and in HepG2 Cells. Anal. Chem. 2016, 88 (14), 7259–7267. 10.1021/acs.analchem.6b01518. 27310767

[ref22] Zhang B.; Liu Y.; Li X.; Xu J.; Fang J. Small Molecules to Target the Selenoprotein Thioredoxin Reductase. Chem.–Asian J. 2018, 13 (23), 3593–3600. 10.1002/asia.201801136. 30204305

[ref23] Wang J.; Chen M.; Zhang Z.; Ma L.; Chen T. Selenium: From Fluorescent Probes to Biomedical Application. Coord. Chem. Rev. 2023, 493, 215278 10.1016/j.ccr.2023.215278.

[ref24] Wang Z.; Yu F.; Xing Y.; Wang R.; Liu H.; Cheng Z.; Jin J.; Zhao L.; Yu F. Molecular Fluorescent Probes for the Detection and Imaging of Sulfane Sulfur and Reactive Selenium Species. In Fluorescent Chemosensors; The Royal Society of Chemistry, 2023; pp 286–325.

[ref25] Wu D.; Chen L.; Kwon N.; Yoon J. Fluorescent Probes Containing Selenium as a Guest or Host. Chem. 2016, 1 (5), 674–698. 10.1016/j.chempr.2016.10.005.

[ref26] Zhang L.; Shi Y.; Sheng Z.; Zhang Y.; Kai X.; Li M.; Yin X. Bioluminescence Imaging of Selenocysteine in Vivo with a Highly Sensitive Probe. ACS Sens. 2019, 4 (12), 3147–3155. 10.1021/acssensors.9b01268. 31701738

[ref27] Han X.; Song X.; Yu F.; Chen L. A Ratiometric Near-Infrared Fluorescent Probe for Quantification and Evaluation of Selenocysteine-Protective Effects in Acute Inflammation. Adv. Funct. Mater. 2017, 27 (28), 1700769 10.1002/adfm.201700769.

[ref28] Sun Q.; Yang S.-H.; Wu L.; Dong Q.-J.; Yang W.-C.; Yang G.-F. Detection of Intracellular Selenol-Containing Molecules Using a Fluorescent Probe with Near-Zero Background Signal. Anal. Chem. 2016, 88 (11), 6084–6091. 10.1021/acs.analchem.6b01545. 27161304

[ref29] Luo X.; Wang R.; Lv C.; Chen G.; You J.; Yu F. Detection of Selenocysteine with a Ratiometric Near-Infrared Fluorescent Probe in Cells and in Mice Thyroid Diseases Model. Anal. Chem. 2020, 92 (1), 1589–1597. 10.1021/acs.analchem.9b04860. 31815453

[ref30] Kong F.; Hu B.; Gao Y.; Xu K.; Pan X.; Huang F.; Zheng Q.; Chen H.; Tang B. Fluorescence Imaging of Selenol in HepG2 Cell Apoptosis Induced by Na _2_ SeO _3_. Chem. Commun. 2015, 51 (15), 3102–3105. 10.1039/C4CC06359G. 25597534

[ref31] Nan Y.; Zhao W.; Xu X.; Au C.-T.; Qiu R. Synthesis, characterization and applications of selenocysteine-responsive nanoprobe based on dinitrobenzene sulfonyl-modified poly(carbonate) micelles. RSC Adv. 2015, 5 (85), 69299–69306. 10.1039/C5RA12314C.

[ref32] Zhang D.; Hu M.; Yuan X.; Wu Y.; Hu X.; Xu S.; Liu H.-W.; Zhang X.; Liu Y.; Tan W. Engineering Self-Calibrating Nanoprobes with Two-Photon-Activated Fluorescence Resonance Energy Transfer for Ratiometric Imaging of Biological Selenocysteine. ACS Appl. Mater. Interfaces 2019, 11 (19), 17722–17729. 10.1021/acsami.9b04555. 30998313

[ref33] Zhang P.; Ding Y.; Liu W.; Niu G.; Zhang H.; Ge J.; Wu J.; Li Y.; Wang P. Red Emissive Fluorescent Probe for the Rapid Detection of Selenocysteine. Sens Actuators B Chem. 2018, 264, 234–239. 10.1016/j.snb.2018.02.185.

[ref34] Zhao M.; Shi D.; Hu W.; Ma T.; He L.; Lu D.; Hu Y.; Zhou L. A Two-Photon “Turn-on” Fluorescent Probe for Both Exogenous and Endogenous Selenocysteine Detection and Imaging in Living Cells and Zebrafish. Spectrochim. Acta, Part A 2021, 260, 119983 10.1016/j.saa.2021.119983. 34052765

[ref35] Hu B.; Cheng R.; Liu X.; Pan X.; Kong F.; Gao W.; Xu K.; Tang B. A Nanosensor for in Vivo Selenol Imaging Based on the Formation of Au Se Bonds. Biomaterials 2016, 92, 81–89. 10.1016/j.biomaterials.2016.03.030. 27043769

[ref36] Liu X.; Hu B.; Cheng R.; Kong F.; Pan X.; Xu K.; Tang B. Simultaneous Fluorescence Imaging of Selenol and Hydrogen Peroxide under Normoxia and Hypoxia in HepG2 Cells and in Vivo. Chem. Commun. 2016, 52 (40), 6693–6696. 10.1039/C6CC02111E. 27115078

[ref37] Chen J.; Gao F.; Xu Z.; Liu Y.; Hu M.; Yuan C.; Zhang Y.; Liu W.; Wang X. A terbium(iii) complex-based time-resolved luminescent probe for selenocysteine as an inhibitor of selenoproteins. Chem. Commun. 2024, 60 (11), 1440–1443. 10.1039/D3CC05680E. 38206371

[ref38] Wang Z.; Hao C.; Luo X.; Wu Q.; Zhang C.; Dessie W.; Jiang Y. A FRET-ICT Dual-Modulated Ratiometric Fluorescence Sensor for Monitoring and Bio-Imaging of Cellular Selenocysteine. Molecules 2020, 25 (21), 4999 10.3390/molecules25214999. 33126726 PMC7663636

[ref39] Cheng D.; Pan Y.; Yin B.-C.; Yuan L.; Zhang X.-B. A New Fluorescent Probe with Ultralow Background Fluorescence for Imaging of Endogenous Cellular Selenol under Oxidative Stress. Chin. Chem. Lett. 2017, 28 (10), 1987–1990. 10.1016/j.cclet.2017.08.021.

[ref40] Maeda H.; Katayama K.; Matsuno H.; Uno T. 3′-(2,4-Dinitrobenzenesulfonyl)-2′,7′-dimethylfluorescein as a Fluorescent Probe for Selenols. Angew. Chem. 2006, 118 (11), 1842–1845. 10.1002/ange.200504299. 16470905

[ref41] Wang Q.; Zhang S.; Zhong Y.; Yang X.-F.; Li Z.; Li H. Preparation of Yellow-Green-Emissive Carbon Dots and Their Application in Constructing a Fluorescent Turn-On Nanoprobe for Imaging of Selenol in Living Cells. Anal. Chem. 2017, 89 (3), 1734–1741. 10.1021/acs.analchem.6b03983. 28208245

[ref42] Tian Y.; Xin F.; Gao C.; Jing J.; Zhang X. Ratiometric Fluorescence Imaging of Endogenous Selenocysteine in Cancer Cell Matrix. J. Mater. Chem. B 2017, 5 (33), 6890–6896. 10.1039/C7TB01558E. 32264338

[ref43] Zhao X.; Yuan G.; Ding H.; Zhou L.; Lin Q. A TP-FRET-Based Fluorescent Sensor for Ratiometric Visualization of Selenocysteine Derivatives in Living Cells, Tissues and Zebrafish. J. Hazard. Mater. 2020, 381, 120918 10.1016/j.jhazmat.2019.120918. 31421550

[ref44] Li M.; Feng W.; Zhai Q.; Feng G. Selenocysteine Detection and Bioimaging in Living Cells by a Colorimetric and Near-Infrared Fluorescent Turn-on Probe with a Large Stokes Shift. Biosens. Bioelectron. 2017, 87, 894–900. 10.1016/j.bios.2016.09.056. 27664408

[ref45] Dai C.-G.; Wang J.-L.; Song Q.-H. Red Fluorescent Probes Based on a Bodipy Analogue for Selective and Sensitive Detection of Selenols in Solutions and in Living Systems. J. Mater. Chem. B 2016, 4 (41), 6726–6733. 10.1039/C6TB02081J. 32263527

[ref46] Wang Z.; Yang S.; Liu X.; Yang T.; Han T.; He X.; Jiang Y.; Hao Y. A Near-Infrared Turn-on Fluorescent Probe for the Rapid Detection of Selenocysteine and Its Application of Imaging in Living Cells and Mice. Microchem. J. 2021, 170, 106681 10.1016/j.microc.2021.106681.

[ref47] Zhang H.; Li M.; Feng W.; Feng G. Rapid and Selective Detection of Selenocysteine with a Known Readily Available Colorimetric and Fluorescent Turn-on Probe. Dyes Pigm. 2018, 149, 475–480. 10.1016/j.dyepig.2017.10.031.

[ref48] Wang Z.; Zheng H.; Zhang C.; Tang D.; Wu Q.; Dessie W.; Jiang Y. A Red Emissive Fluorescent Turn-on Sensor for the Rapid Detection of Selenocysteine and Its Application in Living Cells Imaging. Sensors 2020, 20 (17), 4768 10.3390/s20174768. 32846934 PMC7506812

[ref49] Zhang S.; Wang Q.; Liu X.; Zhang J.; Yang X.-F.; Li Z.; Li H. Sensitive and Selective Fluorescent Probe for Selenol in Living Cells Designed via a p *K* a Shift Strategy. Anal. Chem. 2018, 90 (6), 4119–4125. 10.1021/acs.analchem.8b00066. 29466857

[ref50] Chen H.; Dong B.; Tang Y.; Lin W. Construction of a Near-Infrared Fluorescent Turn-On Probe for Selenol and Its Bioimaging Application in Living Animals. Chem. - Eur. J. 2015, 21 (33), 11696–11700. 10.1002/chem.201502226. 26177833

[ref51] Feng W.; Li M.; Sun Y.; Feng G. Near-Infrared Fluorescent Turn-on Probe with a Remarkable Large Stokes Shift for Imaging Selenocysteine in Living Cells and Animals. Anal. Chem. 2017, 89 (11), 6106–6112. 10.1021/acs.analchem.7b00824. 28504517

[ref52] Zhang B.; Ge C.; Yao J.; Liu Y.; Xie H.; Fang J. Selective Selenol Fluorescent Probes: Design, Synthesis, Structural Determinants, and Biological Applications. J. Am. Chem. Soc. 2015, 137 (2), 757–769. 10.1021/ja5099676. 25562612

[ref53] Ma J.; Xu Y.; Zhao W.; Wang B.; Zhang C.; Zhang Z. Rapid Detection of Thioredoxin Reductase with a Fluorescent Probe *via* a Tag-Sec Method. Mater. Chem. Front. 2021, 5 (23), 8108–8117. 10.1039/D1QM01254A.

[ref54] Zhang L.; Kai X.; Zhang Y.; Zheng Y.; Xue Y.; Yin X.; Zhao J. A Reaction-Based near-Infrared Fluorescent Probe That Can Visualize Endogenous Selenocysteine *in Vivo* in Tumor-Bearing Mice. Analyst 2018, 143 (20), 4860–4869. 10.1039/C8AN00765A. 30128454

[ref55] Han X.; Wang R.; Song X.; Yu F.; Chen L. Evaluation Selenocysteine Protective Effect in Carbon Disulfide Induced Hepatitis with a Mitochondrial Targeting Ratiometric Near-Infrared Fluorescent Probe. Anal. Chem. 2018, 90 (13), 8108–8115. 10.1021/acs.analchem.8b01306. 29862823

[ref56] Tian Y.; Zhang X. Fluorescence Imaging of Bioactive Selenocompounds. Sci. Sin.: Chim. 2024, 54 (10), 1817–1825. 10.1360/SSC-2024-0110.

[ref57] Icha J.; Weber M.; Waters J. C.; Norden C. Phototoxicity in Live Fluorescence Microscopy, and How to Avoid It. BioEssays 2017, 39 (8), 1700003 10.1002/bies.201700003. 28749075

[ref58] Roncaroli F.; Olabe J. A. The Reactions of Nitrosyl Complexes with Cysteine. Inorg. Chem. 2005, 44 (13), 4719–4727. 10.1021/ic048156d. 15962980

[ref59] Souza M. L.; Roveda A. C.; Pereira J. C. M.; Franco D. W. New Perspectives on the Reactions of Metal Nitrosyls with Thiolates as Nucleophiles. Coord. Chem. Rev. 2016, 306, 615–627. 10.1016/j.ccr.2015.03.008.

[ref60] Sharma N.; Kumar V.; Jose D. A. A Ruthenium Nitrosyl Complex-Based Highly Selective Colorimetric Sensor for Biological H _2_ S and H _2_ S–NO Cross-Talk Regulated Release of NO. Dalton Trans. 2023, 52 (3), 675–682. 10.1039/D2DT03108F. 36537888

[ref61] Shimada K.; Goto K.; Kawashima T.; Takagi N.; Choe Y.-K.; Nagase S. Isolation of a *Se-* Nitrososelenol: A New Class of Reactive Nitrogen Species Relevant to Protein *Se-* Nitrosation. J. Am. Chem. Soc. 2004, 126 (41), 13238–13239. 10.1021/ja0457009. 15479074

[ref62] Masuda R.; Kuwano S.; Goto K. Modeling Selenoprotein *Se* -Nitrosation: Synthesis of a *Se* -Nitrososelenocysteine with Persistent Stability. J. Am. Chem. Soc. 2023, 145 (26), 14184–14189. 10.1021/jacs.3c03394. 37267591 PMC10326881

[ref63] Huber R. E.; Criddle R. S. Comparison of the Chemical Properties of Selenocysteine and Selenocystine with Their Sulfur Analogs. Arch. Biochem. Biophys. 1967, 122 (1), 164–173. 10.1016/0003-9861(67)90136-1. 6076213

[ref64] Luo D.; Smith S. W.; Anderson B. D. Kinetics and Mechanism of the Reaction of Cysteine and Hydrogen Peroxide in Aqueous Solution. J. Pharm. Sci. 2005, 94 (2), 304–316. 10.1002/jps.20253. 15570599

[ref65] Turner E.; Hager L. J.; Shapiro B. M. Ovothiol Replaces Glutathione Peroxidase as a Hydrogen Peroxide Scavenger in Sea Urchin Eggs. Science 1988, 242 (4880), 939–941. 10.1126/science.3187533. 3187533

[ref66] Rasmussen K.; Møller J. Total Homocysteine Measurement in Clinical Practice. Ann. Clin. Biochem. Int. J. Lab. Med. 2000, 37 (5), 627–648. 10.1258/0004563001899915. 11026516

[ref67] Hilderbrand S. A.; Lim M. H.; Lippard S. J. Dirhodium Tetracarboxylate Scaffolds as Reversible Fluorescence-Based Nitric Oxide Sensors. J. Am. Chem. Soc. 2004, 126 (15), 4972–4978. 10.1021/ja038471j. 15080703

[ref68] Taha Z. A.; Ajlouni A. M.; Al Momani W.; Al-Ghzawi A. A. Syntheses, characterization, biological activities and photophysical properties of lanthanides complexes with a tetradentate Schiff base ligand. Spectrochim. Acta, Part A 2011, 81 (1), 570–577. 10.1016/j.saa.2011.06.052. 21764359

[ref69] Hardy E. E.; Wyss K. M.; Keller R. J.; Gorden J. D.; Gorden A. E. V. Tunable ligand emission of napthylsalophen triple-decker dinuclear lanthanide(iii) sandwich complexes. Dalton Trans. 2018, 47 (4), 1337–1346. 10.1039/C7DT03733C. 29303180

[ref70] Wu F.; Wang C.-J.; Lin H.; Jia A.-Q.; Zhang Q.-F. Syntheses, structures and catalytic properties of ruthenium(II) nitrosyl complexes with bidentate and tetradentate Schiff base ligands. Inorg. Chim. Acta 2018, 471, 718–723. 10.1016/j.ica.2017.12.004.

[ref71] Kim M.; Park S.; Song D.; Moon D.; You Y.; Lim M.; Lee H.-I. Visible-Light NO Photolysis of Ruthenium Nitrosyl Complexes with N _2_ O _2_ Ligands Bearing π-Extended Rings and Their Photorelease Dynamics. Dalton Trans. 2022, 51 (30), 11404–11415. 10.1039/D2DT01019D. 35822310

[ref72] Paschenko S. V.; Khramtsov V. V.; Skatchkov M. P.; Plyusnin V. F.; Bassenge E. EPR and Laser Flash Photolysis Studies of the Reaction of Nitric Oxide with Water Soluble NO Trap Fe(II)-Proline-Dithiocarbamate Complex. Biochem. Biophys. Res. Commun. 1996, 225 (2), 577–584. 10.1006/bbrc.1996.1214. 8753803

[ref73] Yoshimura T.; Kotake Y. Spin Trapping of Nitric Oxide with the Iron-Dithiocarbamate Complex: Chemistry and Biology. Antioxid. Redox Signaling 2004, 6 (3), 639–647. 10.1089/152308604773934404. 15130291

[ref74] Katayama Y.; Soh N.; Maeda M. A New Strategy for the Design of Molecular Probes for Investigating Endogenous Nitric Oxide Using an EPR or Fluorescent Technique. ChemPhysChem 2001, 2 (11), 655–661. 10.1002/1439-7641(20011119)2:11<655::AID-CPHC655>3.0.CO;2-S. 23686899

[ref75] Carta F.; Aggarwal M.; Maresca A.; Scozzafava A.; McKenna R.; Masini E.; Supuran C. T. Dithiocarbamates Strongly Inhibit Carbonic Anhydrases and Show Antiglaucoma Action in Vivo. J. Med. Chem. 2012, 55 (4), 1721–1730. 10.1021/jm300031j. 22276570 PMC3313672

[ref76] Cameron B. R.; Darkes M. C.; Baird I. R.; Skerlj R. T.; Santucci Z. L.; Fricker S. P. Ruthenium(III) Triazacyclononane Dithiocarbamate, Pyridinecarboxylate, or Aminocarboxylate Complexes as Scavengers of Nitric Oxide. Inorg. Chem. 2003, 42 (13), 4102–4108. 10.1021/ic020283r. 12817968

[ref77] Bruker AXS Inc. Bruker SAINT and SADABS; Bruker AXS Inc.: Madison, Wisconsin: USA, 2007.

[ref78] Sheldrick G. M. A Short History of SHELX. Acta Crystallogr., Sect. A:Found. Crystallogr 2008, 64 (1), 112–122. 10.1107/S0108767307043930. 18156677

[ref79] Hübschle C. B.; Sheldrick G. M.; Dittrich B. ShelXle: A Qt Graphical User Interface for SHELXL. J. Appl. Crystallogr. 2011, 44 (6), 1281–1284. 10.1107/S0021889811043202. 22477785 PMC3246833

[ref80] Fry N. L.; Heilman B. J.; Mascharak P. K. Dye-Tethered Ruthenium Nitrosyls Containing Planar Dicarboxamide Tetradentate N4 Ligands: Effects of In-Plane Ligand Twist on NO Photolability. Inorg. Chem. 2011, 50 (1), 317–324. 10.1021/ic1019873. 21114262

[ref81] Rose M. J.; Olmstead M. M.; Mascharak P. K. Photoactive Ruthenium Nitrosyls Derived from Quinoline- and Pyridine-Based Ligands: Accelerated Photorelease of NO Due to Quinoline Ligation. Polyhedron 2007, 26 (16), 4713–4718. 10.1016/j.poly.2007.03.010.

[ref82] Mudrak V.; Lacroix P. G.; Tassé M.; Mallet-Ladeira S.; Roshal A.; Malfant I. Ruthenium Nitrosyl Complexes with NO Release Capability: The Use of Fluorene as an Antenna. Dalton Trans. 2024, 53 (23), 9777–9791. 10.1039/D4DT01154F. 38780443

[ref83] Teng L.; Zhang Y.; Zhang S.; Qu Y.; Xia X. 5-(1 H -Imidazole-1-Ylsulfonyl)- N, N -Dimethylnaphthalen-1-Amine. Acta Crystallogr Sect E Struct Rep Online 2009, 65 (1), o55 10.1107/S160053680804066X. PMC2967969 21581696

[ref84] Stepanenko I.; Zalibera M.; Schaniel D.; Telser J.; Arion V. B. Ruthenium-Nitrosyl Complexes as NO-Releasing Molecules, Potential Anticancer Drugs, and Photoswitches Based on Linkage Isomerism. Dalton Trans. 2022, 51 (14), 5367–5393. 10.1039/D2DT00290F. 35293410

[ref85] Fry N. L.; Mascharak P. K. Photoactive Ruthenium Nitrosyls as NO Donors: How To Sensitize Them toward Visible Light. Acc. Chem. Res. 2011, 44 (4), 289–298. 10.1021/ar100155t. 21361269

[ref86] Kumar S.; Singh S.; Ghosh K. Ruthenium Nitrosyl Complexes: Photoinduced Delivery of NO to Different Biological Targets; Springer International Publishing, 2023; pp 425–445.

[ref87] Lopes L. G. F.; Castellano E. E.; Ferreira A. G.; Davanzo C. U.; Clarke M. J.; Franco D. W. Reactivity of Trans-[Ru(NH3)4P(OEt)3NO]X3: (X = PF6-, CF3COO-): Modulation of the Release of NO by the Trans-Effect. Inorg. Chim. Acta 2005, 358 (10), 2883–2890. 10.1016/j.ica.2004.07.014.

[ref88] Silva F. O. N.; Cândido M. C. L.; Holanda A. K. M.; Diógenes I. C. N.; Sousa E. H. S.; Lopes L. G. F. Mechanism and Biological Implications of the NO Release of Cis-[Ru(Bpy)2L(NO)]N+ Complexes: A Key Role of Physiological Thiols. J. Inorg. Biochem. 2011, 105 (5), 624–629. 10.1016/j.jinorgbio.2011.02.004. 21443852

[ref89] Toledo J. C.; dos Santos Lima Neto B.; Franco D. W. Mutual Effects in the Chemical Properties of the Ruthenium Metal Center and Ancillary Ligands upon Coordination. Coord. Chem. Rev. 2005, 249 (3–4), 419–431. 10.1016/j.ccr.2004.09.016.

[ref90] Richaud A.; Barba-Behrens N.; Méndez F. Chemical Reactivity of the Imidazole: A Semblance of Pyridine and Pyrrole?. Org. Lett. 2011, 13 (5), 972–975. 10.1021/ol103011h. 21268606

[ref91] Tfouni E.; Truzzi D. R.; Tavares A.; Gomes A. J.; Figueiredo L. E.; Franco D. W. Biological Activity of Ruthenium Nitrosyl Complexes. Nitric Oxide 2012, 26 (1), 38–53. 10.1016/j.niox.2011.11.005. 22178685

[ref92] Navale G. R.; Singh S.; Ghosh K. NO Donors as the Wonder Molecules with Therapeutic Potential: Recent Trends and Future Perspectives. Coord. Chem. Rev. 2023, 481, 215052 10.1016/j.ccr.2023.215052.

[ref93] Lewandowska H. Coordination Chemistry of Nitrosyls and Its Biochemical Implications. Nitrosyl Complexes in Inorganic Chemistry, Biochemistry and Medicine I; Springer Berlin Heidelberg, 2013; pp 45–114.

[ref94] Becker T.; Kupfer S.; Wolfram M.; Görls H.; Schubert U. S.; Anslyn E. V.; Dietzek B.; Gräfe S.; Schiller A. Sensitization of NO-Releasing Ruthenium Complexes to Visible Light. Chem. - Eur. J. 2015, 21 (44), 15554–15563. 10.1002/chem.201502091. 26394612

[ref95] Toledo J. C.; Silva H. A. S.; Scarpellini M.; Mori V.; Camargo A. J.; Bertotti M.; Franco D. W. Ruthenium Tetraammines as a Model of Nitric Oxide Donor Compounds. Eur. J. Inorg. Chem. 2004, 2004 (9), 1879–1885. 10.1002/ejic.200300683.

[ref96] Tfouni E.; Krieger M.; McGarvey B. R.; Franco D. W. Structure, Chemical and Photochemical Reactivity and Biological Activity of Some Ruthenium Amine Nitrosyl Complexes. Coord. Chem. Rev. 2003, 236 (1–2), 57–69. 10.1016/S0010-8545(02)00177-7.

[ref97] Amendola V.; Fabbrizzi L.; Foti F.; Licchelli M.; Mangano C.; Pallavicini P.; Poggi A.; Sacchi D.; Taglietti A. Light-Emitting Molecular Devices Based on Transition Metals. Coord. Chem. Rev. 2006, 250 (3–4), 273–299. 10.1016/j.ccr.2005.04.022.

[ref98] de Souza Góis R. G.; Boffo E. F.; Toledo Júnior J. C.; Andriani K. F.; Caramori G. F.; de Jesus Gomes A.; Doro F. G. A Ruthenium Nitrosyl Cyclam Complex with Appended Anthracenyl Fluorophore. Polyhedron 2019, 173, 114117 10.1016/j.poly.2019.114117.

[ref99] Lim M. H.; Lippard S. J. Fluorescence-Based Nitric Oxide Detection by Ruthenium Porphyrin Fluorophore Complexes. Inorg. Chem. 2004, 43 (20), 6366–6370. 10.1021/ic035418n. 15446885

[ref100] G U. R.; Axthelm J.; Hoffmann P.; Taye N.; Gläser S.; Görls H.; Hopkins S. L.; Plass W.; Neugebauer U.; Bonnet S.; Schiller A. Co-Registered Molecular Logic Gate with a CO-Releasing Molecule Triggered by Light and Peroxide. J. Am. Chem. Soc. 2017, 139 (14), 4991–4994. 10.1021/jacs.7b00867. 28345936

[ref101] Jimenez J.; Chakraborty I.; Dominguez A.; Martinez-Gonzalez J.; Sameera W. M. C.; Mascharak P. K. A Luminescent Manganese PhotoCORM for CO Delivery to Cellular Targets under the Control of Visible Light. Inorg. Chem. 2018, 57 (4), 1766–1773. 10.1021/acs.inorgchem.7b02480. 29393641

[ref102] Rose M. J.; Mascharak P. K. A Photosensitive {Ru–NO}6 Nitrosyl Bearing Dansyl Chromophore: Novel NO Donor with a Fluorometric on/off Switch. Chem. Commun. 2008, 33, 3933 10.1039/b805332d. 18726039

[ref103] deBoer-Maggard T. R.; Fry N. L.; Mascharak P. K. Evidence of Dexter Energy Transfer in NO Photolability of Dye-Sensitized Ruthenium Nitrosyls. Inorg. Chim. Acta 2013, 406, 190–195. 10.1016/j.ica.2013.04.034.

[ref104] Works C. F.; Jocher C. J.; Bart G. D.; Bu X.; Ford P. C. Photochemical Nitric Oxide Precursors: Synthesis, Photochemistry, and Ligand Substitution Kinetics of Ruthenium Salen Nitrosyl and Ruthenium Salophen Nitrosyl Complexes ^1^. Inorg. Chem. 2002, 41 (14), 3728–3739. 10.1021/ic020248k. 12099878

[ref105] Bordini J.; Hughes D. L.; Da Motta Neto J. D.; Jorge da Cunha C. Nitric Oxide Photorelease from Ruthenium Salen Complexes in Aqueous and Organic Solutions. Inorg. Chem. 2002, 41 (21), 5410–5416. 10.1021/ic011273d. 12377035

[ref106] Pereira J. C. M.; Souza M. L.; Franco D. W. Nitric Oxide and Nitroxyl Products from the Reaction of < scp > L</Scp> -Cysteine with *Trans* -[RuNO(NH _3_) _4_ P(OEt) _3_ ](PF _6_) _3_. Eur. J. Inorg. Chem. 2015, 2015 (6), 1005–1011. 10.1002/ejic.201402992.

[ref107] Zhang C.; Qiu Z.; Zhang L.; Pang Q.; Yang Z.; Qin J.-K.; Liang H.; Zhao S. Design and Synthesis of a Ratiometric Photoacoustic Imaging Probe Activated by Selenol for Visual Monitoring of Pathological Progression of Autoimmune Hepatitis. Chem. Sci. 2021, 12 (13), 4883–4888. 10.1039/D0SC06573K. 34163738 PMC8179563

[ref108] Suzuki N.; Ogra Y. 77Se NMR Spectroscopy for Speciation Analysis of Selenium Compounds. In Metallomics; Springer Japan: Tokyo, 2017; pp 147–155.

[ref109] Tan K.-S.; Arnold A. P.; Rabenstein D. L. Selenium-77 Nuclear Magnetic Resonance Studies of Selenols, Diselenides, and Selenenyl Sulfides. Can. J. Chem. 1988, 66 (1), 54–60. 10.1139/v88-008.

[ref110] Pálla T.; Herbath L.; Mazák K.; Mirzahosseini A.; Noszál B. Selenate—An Internal Chemical Shift Standard for Aqueous ^77^ Se NMR Spectroscopy. Magn. Reson. Chem. 2022, 60 (1), 148–156. 10.1002/mrc.5196. 34273131

[ref111] Cho J.-H.; Kim M.; You Y.; Lee H.-I. A New Photoactivable NO-releasing {Ru–NO} ^6^ Ruthenium Nitrosyl Complex with a Tetradentate Ligand Containing Aniline and Pyridine Moieties. Chem.–Asian J. 2022, 17 (2), e202101244 10.1002/asia.202101244. 34921511

[ref112] Roose M.; Tassé M.; Lacroix P. G.; Malfant I. Nitric Oxide (NO) Photo-Release in a Series of Ruthenium–Nitrosyl Complexes: New Experimental Insights in the Search for a Comprehensive Mechanism. New J. Chem. 2019, 43 (2), 755–767. 10.1039/C8NJ03907K.

[ref113] Yang W.; Mortier W. J. The Use of Global and Local Molecular Parameters for the Analysis of the Gas-Phase Basicity of Amines. J. Am. Chem. Soc. 1986, 108 (19), 5708–5711. 10.1021/ja00279a008. 22175316

[ref114] Maples D. L.; Maples R. D.; Hoffert W. A.; Parsell T. H.; Asselt A. V.; Silversides J. D.; Archibald S. J.; Hubin T. J. Synthesis and Characterization of the Chromium(III) Complexes of Ethylene Cross-Bridged Cyclam and Cyclen Ligands. Inorg. Chim. Acta 2009, 362 (6), 2084–2088. 10.1016/j.ica.2008.09.034. PMC2746693 20161052

[ref115] Becke A. D. Density-Functional Exchange-Energy Approximation with Correct Asymptotic Behavior. Phys. Rev. A 1988, 38 (6), 3098–3100. 10.1103/PhysRevA.38.3098. 9900728

[ref116] Becke A. D. Current-Density Dependent Exchange-Correlation Functionals. Can. J. Chem. 1996, 74 (6), 995–997. 10.1139/v96-110.

[ref117] Lee C.; Yang W.; Parr R. G. Development of the Colle-Salvetti Correlation-Energy Formula into a Functional of the Electron Density. Phys. Rev. B 1988, 37 (2), 785–789. 10.1103/PhysRevB.37.785. 9944570

[ref118] Montiel E.; Jayanthi N.; Veloz M. A.; Pandiyan T.; Cruz J. Theoretical Studies on Iron Surface Coating: Adsorption of Furan Derivatives Over Fe *n* Clusters (*n* = 1–4). J. Nanosci. Nanotechnol. 2011, 11 (6), 5483–5490. 10.1166/jnn.2011.3440. 21770208

[ref119] Stephens P. J.; Devlin F. J.; Chabalowski C. F.; Frisch M. J. Ab Initio Calculation of Vibrational Absorption and Circular Dichroism Spectra Using Density Functional Force Fields. J. Phys. Chem. 1994, 98 (45), 11623–11627. 10.1021/j100096a001.

[ref120] Gao H. Theoretical Studies of Molecular Structures and Properties of Platinum (II) Antitumor Drugs. Spectrochim. Acta, Part A 2011, 79 (3), 687–693. 10.1016/j.saa.2011.04.006. 21543252

[ref121] Hay P. J.; Wadt W. R. *Ab Initio* Effective Core Potentials for Molecular Calculations. Potentials for K to Au Including the Outermost Core Orbitals. J. Chem. Phys. 1985, 82 (1), 299–310. 10.1063/1.448975.

[ref122] Man W.-L.; Kwong H.-K.; Lam W. W. Y.; Xiang J.; Wong T.-W.; Lam W.-H.; Wong W.-T.; Peng S.-M.; Lau T.-C. General Synthesis of (Salen)Ruthenium(III) Complexes via N···N Coupling of (Salen)Ruthenium(VI) Nitrides. Inorg. Chem. 2008, 47 (13), 5936–5944. 10.1021/ic800263n. 18510309

[ref123] Granados J. A. O.; Hernández J. G.; Huerta-Aguilar C. A.; Thangarasu P. Exploration of Ruthenium Complex of (E)-2-((Pyridine-2-Yl)Methyleneamino) Benzoic Acid as Chemosensor for Simultaneous Recognition of Acetate and HSO4– Ions in Cell Bio-Imaging: Experimental and Theoretical Studies. Sens Actuators B Chem. 2018, 270, 570–581. 10.1016/j.snb.2018.04.113.

