# Supplementary material for: New Fluorescent Chemodosimetric Mechanism for Selective Recognition of Selenocysteine by Dansyl-Appended Ruthenium Nitrosyl Complexes
Source: Inorg Chem. 2025 Feb 20;64(8):3989–4004. doi: 10.1021/acs.inorgchem.4c05277 (PMC11881044; doi:10.1021/acs.inorgchem.4c05277)
Supplement: Supplementary file 1 — ic4c05277_si_001.pdf [file ic4c05277_si_001.pdf]

A New Fluorescent Chemodosimetric Mechanism for Selective Recognition of Selenocysteine by Dansyl-Appended Ruthenium Nitrosyl Complexes

Iván J. Bazany-Rodríguez [a*], Pandiyan Thangarasu [a*], M. Leticia Almada-Leyva [a], José Guadalupe Hernández [b], Diego Martínez-Otero [c], María K. Salomón-Flores [d], Alejandro Dorazco-González [d].

[a]*Facultad de Química, Universidad Nacional Autónoma de México, C.P. 04510, Coyoacán, CDMX, Mexico.*
[b]*Centro Tecnológico, Facultad de Estudios Superiores (FES-Aragón) UNAM, C. P. 57130, Nezahualcóyotl, Estado de México, México.*
[c]*Centro Conjunto de Investigación en Química Sustentable, UAEM-UNAM, C. P. 50200, Toluca, Estado de México, México.*
[d]*Instituto de Química, Universidad Nacional Autónoma de México, C.P. 04510, Coyoacán, CDMX, Mexico.*

**Corresponding Authors*
E-mails: pandiyan@unam.mx (Pandiyan Thangarasu), ivanbazany@comunidad.unam.mx (Iván J. Bazany-Rodríguez).

Supporting Information

Scheme S1 General synthesis of all ligands and complexes.

Scheme S2 Molecular structures, bond distances, and orbital energies (dz^2 and dx^2-y^2) for **[RuL(NO)(ID]$^+$** and **[RuL(H_2O)(ID)]**.

Scheme S3 Molecular structures, bond distances, and orbital energies (dz^2 and dx^2-y^2) for **[RuL(NO)(BD]$^+$** and **[RuL(H_2O)(BD)]**.

Figure S1 ^1H NMR (300 MHz) spectrum of **ID** in DMSO-d_6.

Figure S2 ^1H NMR (300 MHz) spectrum of **BD** in DMSO-d_6.

Figure S3 ^1H NMR (300 MHz) spectrum of **LH_2** in DMSO-d_6.

Figure S4 ^1H NMR (300 MHz) spectrum of **[RuL(NO)Cl]** in DMSO-d_6.

Figure S5 IR (ATR) spectrum of **[RuL(NO)Cl]**.

Figure S6 ^1H NMR (300 MHz) spectrum of **[RuL(NO)(ID)]Cl** in DMSO-d_6.

Figure S7 ^{13}C NMR (75 MHz) spectrum of **[RuL(NO)(ID)]Cl** in DMSO-d_6.

Figure S8 Positive scan MS MALDI-TOF spectrum of **[RuL(NO)(ID)][Cl]** in MeCN.

Figure S9 Partial positive scan MS MALDI TOF spectrum of **[RuL(NO)(ID)][Cl]** in MeCN.

Figure S10 IR (ATR) spectrum of **[RuL(NO)(ID)]Cl**.

Figure S11 ^1H NMR (300 MHz) spectrum of **[RuL(NO)(BD)]Cl** in DMSO-d_6.

Figure S12 ^{13}C NMR (75 MHz) spectrum of **[RuL(NO)(BD)]Cl** in DMSO-d_6.

Figure S13 Positive scan MS MALDI-TOF spectrum of **[RuL(NO)(BD)][Cl]** in MeCN.

Figure S14 Partial positive scan MS MALDI TOF spectrum of **[RuL(NO)(BD)][Cl]** in MeCN.

Figure S15 IR (ATR) spectrum of **[RuL(NO)(BD)]Cl**.

Figure S16 (A) UV/Vis absorption spectra and (B) emission spectra (λ_{ex} = 340 nm) of **[RuL(NO)(ID)]Cl** and **[RuL(NO)(BD)]Cl** solutions (10 µM) upon the addition of increasing amounts of NaSH at pH 7.4 (HEPES 20 mM, 25 °C).

Figure S17 (A) UV/Vis absorption spectra and (B) emission spectra (λ_{ex} = 340 nm) of **[RuL(NO)(ID)]Cl** and **[RuL(NO)(BD)]Cl** solutions (10 μM) upon the addition of increasing amounts of Cys at pH 7.4 (HEPES 20 mM, 25 °C).

Figure S18 (A) UV/Vis absorption spectra and (B) emission spectra (λ_{ex} = 340 nm) of **[RuL(NO)(ID)]Cl** and **[RuL(NO)(BD)]Cl** solutions (10 μM) upon the addition of increasing amounts of Hcy at pH 7.4 (HEPES 20 mM, 25 °C).

Figure S19 (A) UV/Vis absorption spectra and (B) emission spectra (λ_{ex} = 340 nm) of **[RuL(NO)(ID)]Cl** and **[RuL(NO)(BD)]Cl** solutions (10 μM) upon the addition of increasing amounts of GSH at pH 7.4 (HEPES 20 mM, 25 °C).

Figure S20 Positive scan MS MALDI-TOF spectrum of **[RuL(NO)(ID)][Cl]** after treatment with 4.0 equiv. of Sec in MeOH-H_2O (2:1 v/v).

Figure S21 Partial positive scan MS MALDI-TOF spectrum of **[RuL(NO)(ID)][Cl]** after treatment with 4.0 equiv. of Sec in MeOH-H_2O (2:1 v/v).

Figure S22 Positive scan MS MALDI-TOF spectrum of **[RuL(NO)(BD)][Cl]** after treatment with 4.0 equiv. of Sec in MeOH-H_2O (2:1 v/v).

Figure S23 Partial positive scan MS MALDI-TOF spectrum of **[RuL(NO)(BD)][Cl]** after treatment with 4.0 equiv. of Sec in MeOH-H_2O (2:1 v/v).

Figure S24 IR spectra of (A) **[RuL(NO)(ID)][Cl]** and (B) **[RuL(NO)(BD)][Cl]** before and after treatment with 4.0 equiv. of Sec

Figure S25 Molecular orbital: a) **[RuL(NO)(ID)]$^+$**, b) **[RuL(OH$_2$)(ID)]** at gaseous state.

Figure S26 Molecular orbital: a) **[RuL(NO)(BD)]$^+$**, b) **[RuL(OH$_2$)(BD)]** at gaseous state.

Figure S27 Frontier molecular orbital energy level diagram: a) **[RuL(NO)(ID)]$^+$** and b) its adduct **[RuL(OH$_2$)(ID)]** at gaseous state.

Figure S28 Frontier molecular orbital energy level diagram: a) **[RuL(NO)(BD)]$^+$** and b) its adduct **[RuL(OH$_2$)(BD)]** at gaseous state.

Figure S29 Visualization of the electron density isosurface determined by B3LYP/DGDZVP, HOMO and LUMO contour plots (isosurface value = 0.05 au) of the complexes: a) **[RuL(NO)(BD)]$^+$**, b) **[RuL(OH$_2$)(BD)]**, c) **[RuL(NO)(ID)]$^+$** and **[RuL(OH$_2$)(NO)]**.

Figure S30 The fluorescence enhancement of aqueous solution (10 μM) of **[RuL(NO)(ID)]Cl** and **[RuL(NO)(BD)]Cl** upon additions of different bioanalytes (40 μM) at pH 7.4 (HEPES 20 mM).

Table S1 Crystal data and structure refinement for **ID**.

Table S2 Crystal data and structure refinement for **BD**.

Table S3 Bond lengths (Å) and bond angles (°) resulted for nitrosyl complexes and aquo-complexes, using functional B3LYP/6-31G**, LANL2DZ basis set at level of theory.

General considerations

All reagents for synthesis and analysis were of analytical grade and used without further purification: 1,2-phenylenediamine (Aldrich, 99.5%), potassium pentachloronitrosylruthenate(II) (Aldrich, 98%), cesium carbonate (Aldrich, 99.9%), 2-hydroxy-1-naphthaldehyde (Aldrich, 98%), tetrahydrofuran (THF) (J.T.Baker, 99.9%), ethanol HPLC (EtOH) (Fermont, 89.9%), methanol HPLC (MeOH) (Aldrich, ≥99.9%), diethyl ether (Et_2O) (Analytyka, 98.5%), *n*-hexane (Fermont, 99.9%), HEPES buffer solution (Gibco, 1M), MES (Aldrich, 99%), TRIS (J.T.Baker, 99.9%), dansyl chloride (Aldrich, 99%), sodium hydroxide (Aldrich, 98%), ethyl acetate (Aldrich, 99.9%), benzimidazole (Acros, 98%), imidazole (Acros, 99%), triethylamine (NEt_3) (J.T.Baker, 99.9%), *N,N*-dimethylformamide (DMF) (J.T.Baker, 99.9%), carbon disulfide (Aldrich, 99.9%), L-proline (Aldrich, 99%), seleno-L-cystine (Aldrich, 95%), DL-ditiotreitol (Aldrich, 97%), biothiols (analytical standard): L-cysteine (Aldrich), L-homocysteine (Aldrich), glutathione reduced (Aldrich) and sodium hydrosulfide hydrate (Aldrich). Sodium acetate (Aldrich, 99.0%), sodium phosphate dibasic (Aldrich, 99.0%), sodium chloride (Aldrich, 99.0%), potassium chloride (Aldrich, 99.0%), sodium iodide (Aldrich, 99.5%), magnesium chloride (Aldrich, 98.0%), sodium bicarbonate (Aldrich, 99.7%), calcium chloride (Aldrich, 97.0%), sodium sulfate (Aldrich, 99.0%), iron(II) sulfate heptahydrate (Aldrich, 99.0%). Solvents were purified and dried using standard procedures. Deuterated solvents were purchased from Aldrich. Buffer solutions (HEPES 20 mM, pH 7.4) were prepared with double distilled water. The stock solutions of the complexes were prepared in MeCN (spectrophotometric grade).

MALDI-TOF mass spectra were obtained with a Bruker Microflex instrument. ^{1}H, ^{13}C, and ^{77}Se NMR spectra were recorded on a Bruker Advance DPX 300 spectrometer at 300 MHz. Combustion analysis was performed with a Thermo Scientific Flash 2000 Organic Elemental Analyzer. UV-Vis absorption spectra were recorded with a spectrophotometer from PerkinElmer (Lambda 25), and for the fluorescence studies, a F96 Pro spectrophotometer was used. IR spectra were determined with a FT-IR Shimadzu spectrophotometer, IR Prestige-21, from 4000–400 cm^{-1}. EPR spectra were obtained with a Jeol JES-TE300 spectrometer operating in X-Band fashions at 100 kHz modulation frequency and a cylindrical cavity in the mode TE011.

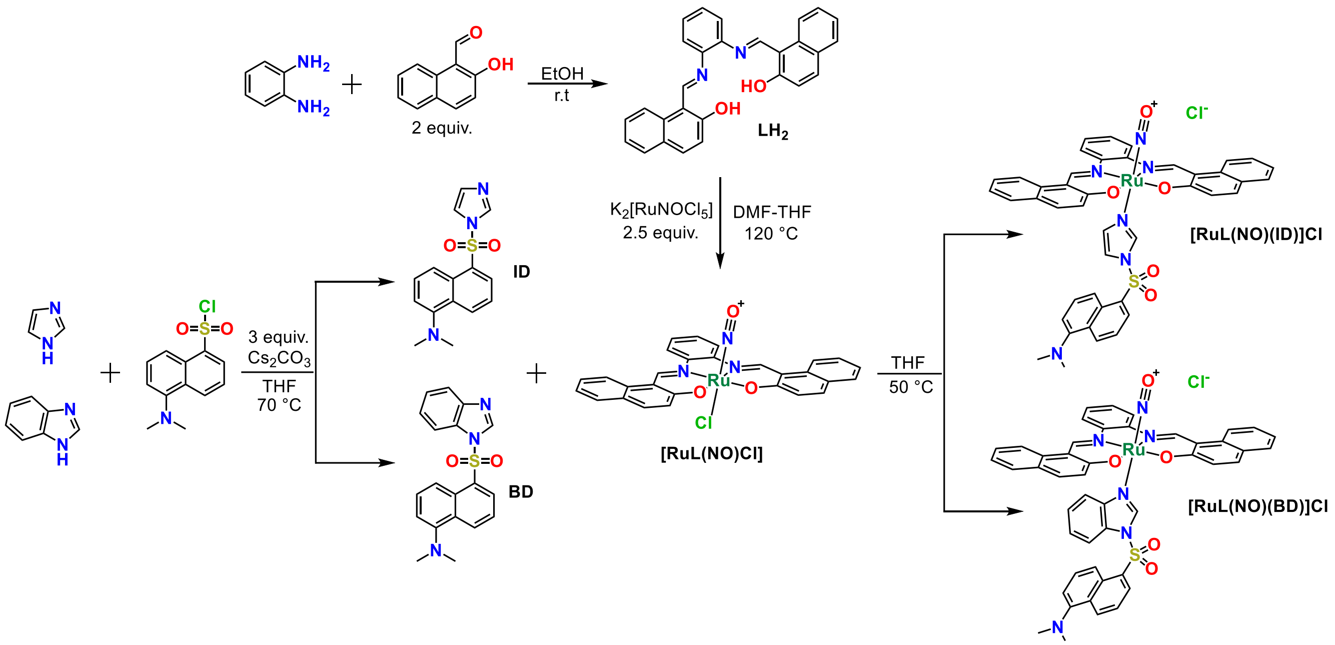

Scheme S1 General synthesis of all ligands and complexes.

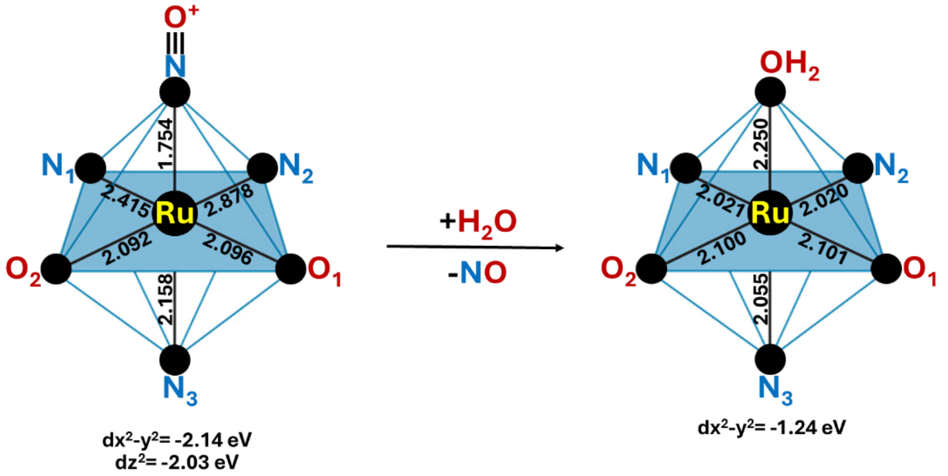

Scheme S2 Molecular structures, bond distances, and orbital energies (dz^2 and $dx^2{-}y^2$) for **[RuL(NO)(ID)]$^+$** and **[RuL(H_2O)(ID)]**.

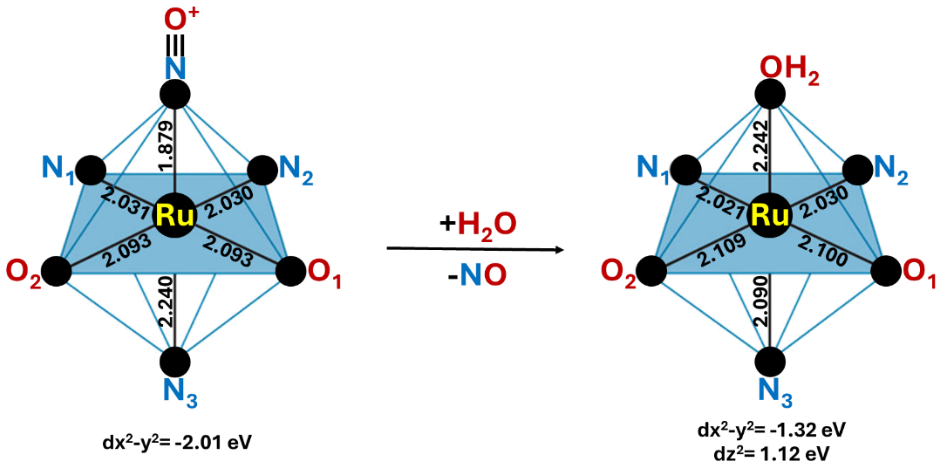

Scheme S3 Molecular structures, bond distances, and orbital energies (dz^2 and $dx^2{-}y^2$) for **[RuL(NO)(BD)]$^+$** and **[RuL(H_2O)(BD)]**.

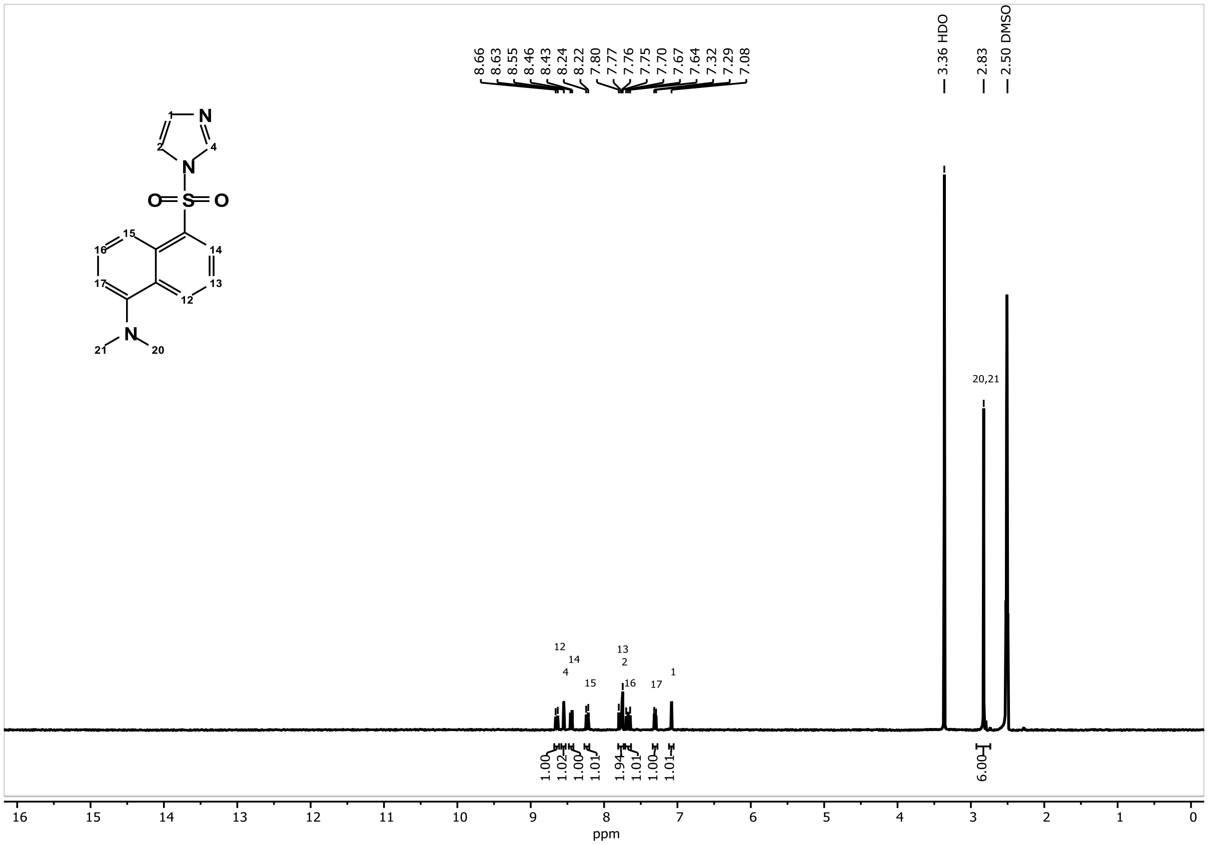

Figure S1 ^{1}H NMR (300 MHz) spectrum of **ID** in DMSO-d_6.

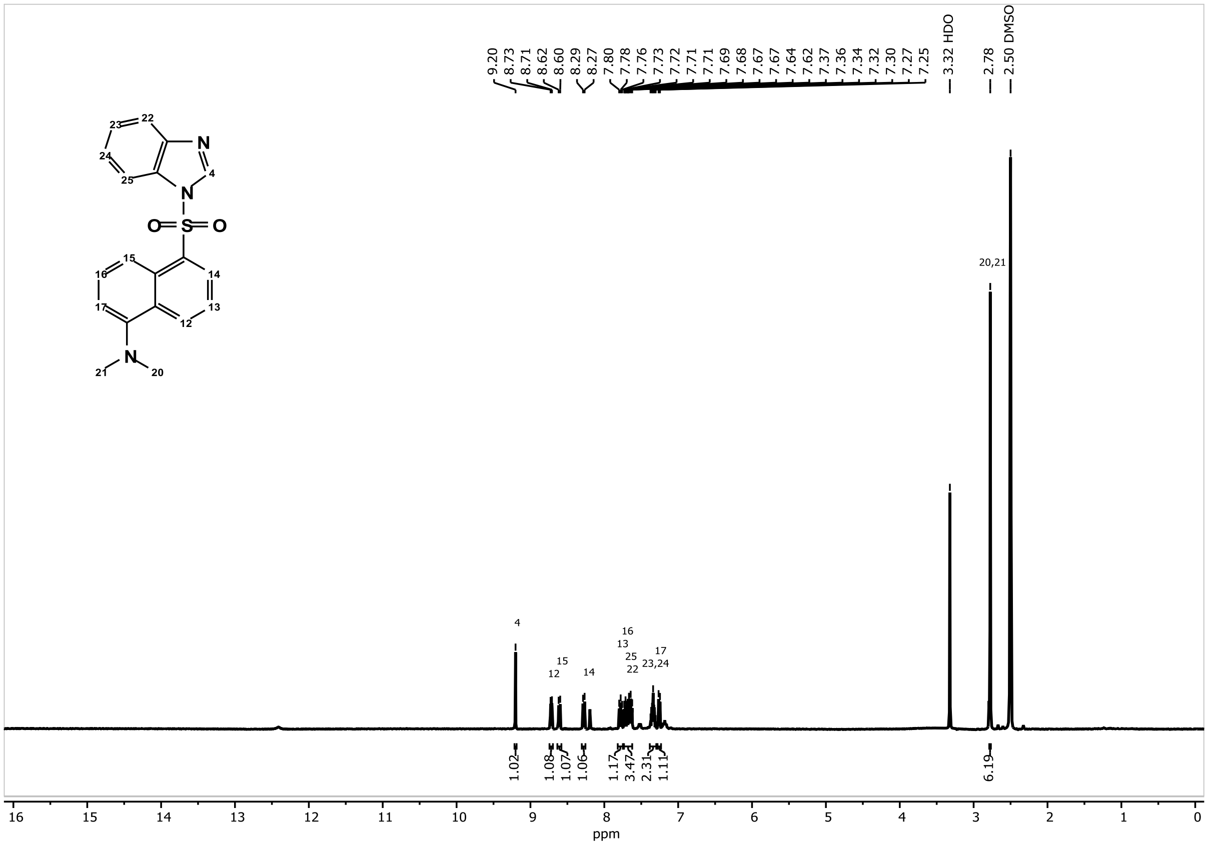

Figure S2 ^{1}H NMR (300 MHz) spectrum of **BD** in DMSO-d_6.

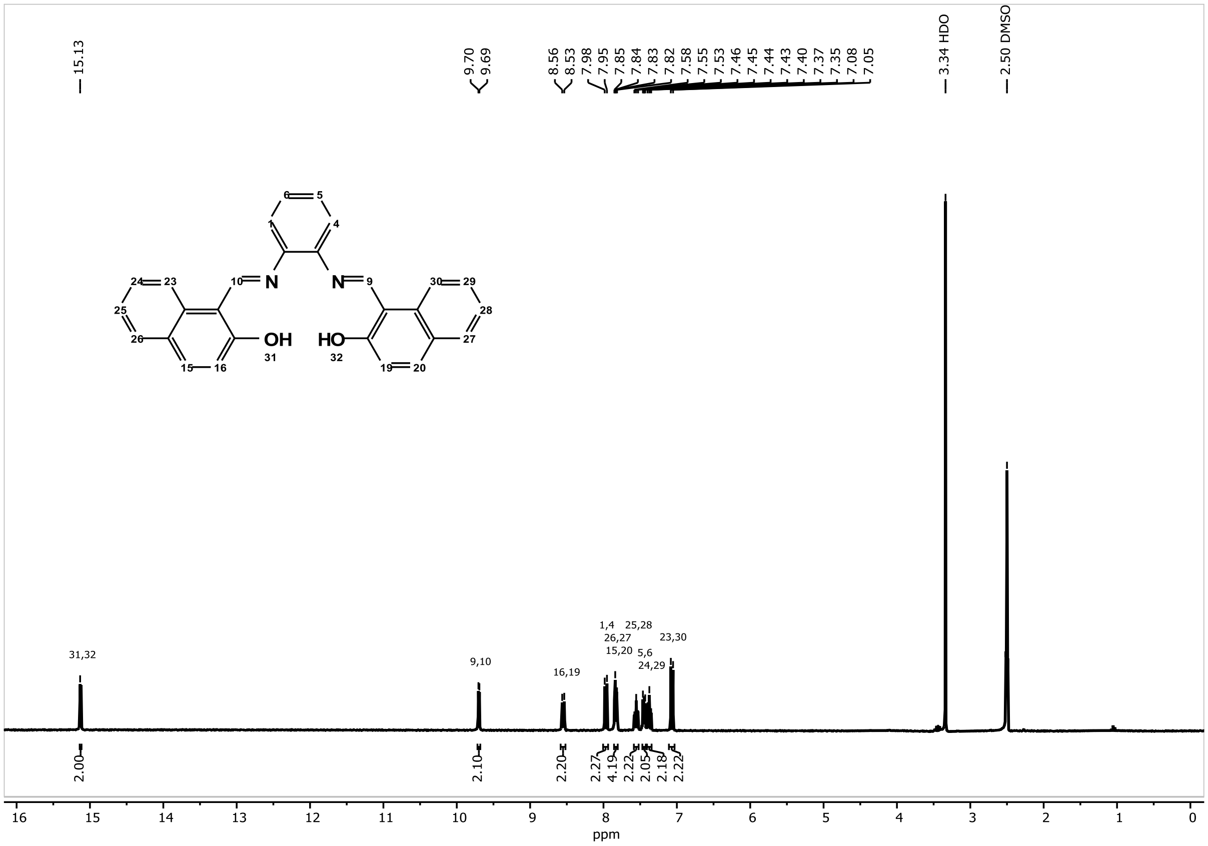

Figure S3 ^{1}H NMR (300 MHz) spectrum of **LH_2** in DMSO-d_6.

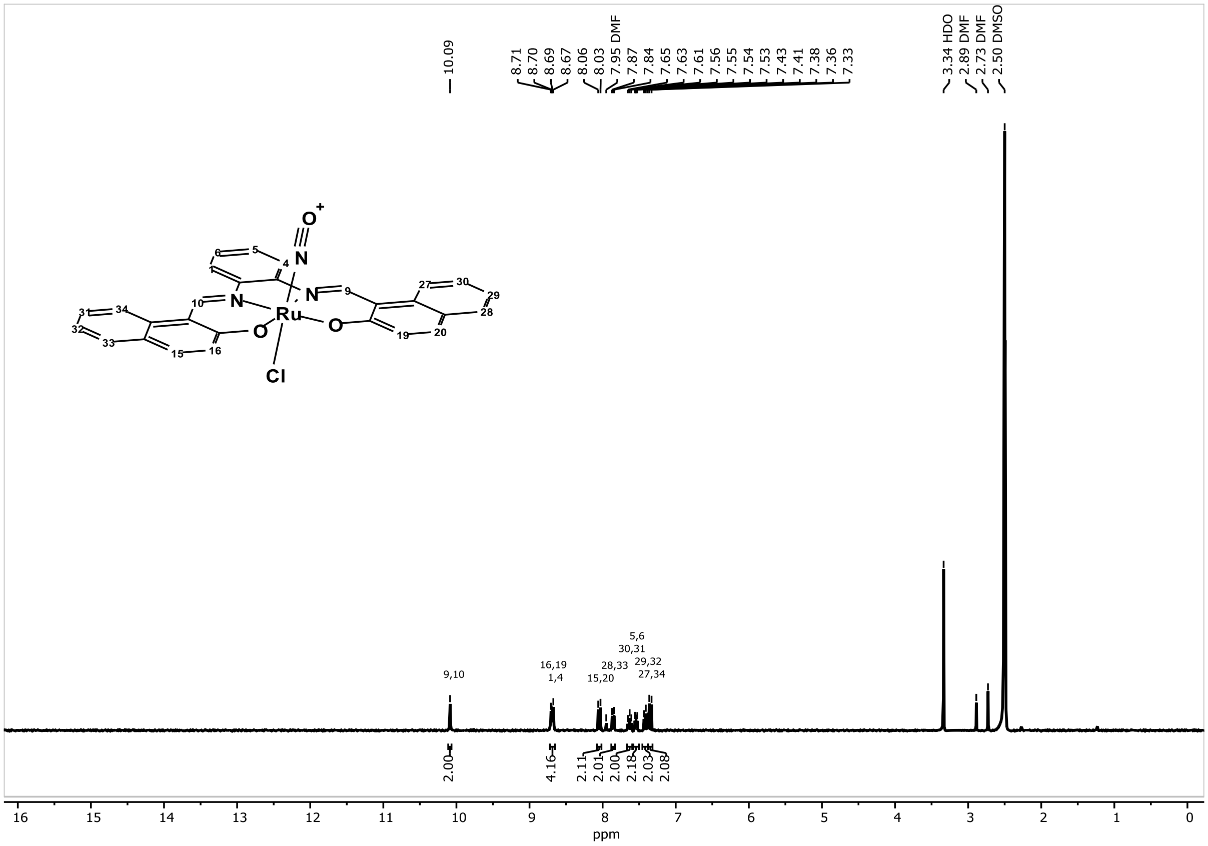

Figure S4 ^{1}H NMR (300 MHz) spectrum of **[RuL(NO)Cl]** in DMSO-d_6.

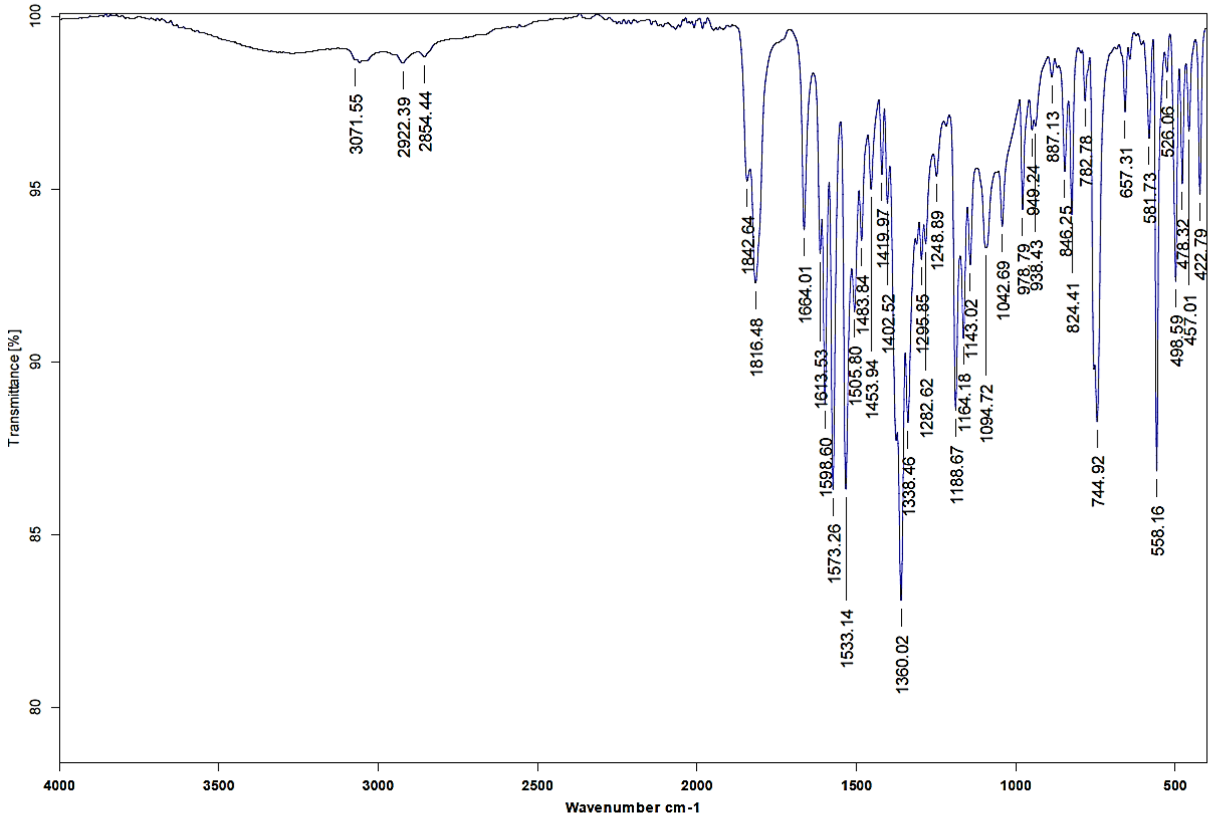

Figure S5 IR (ATR) spectrum of **[RuL(NO)Cl]**.

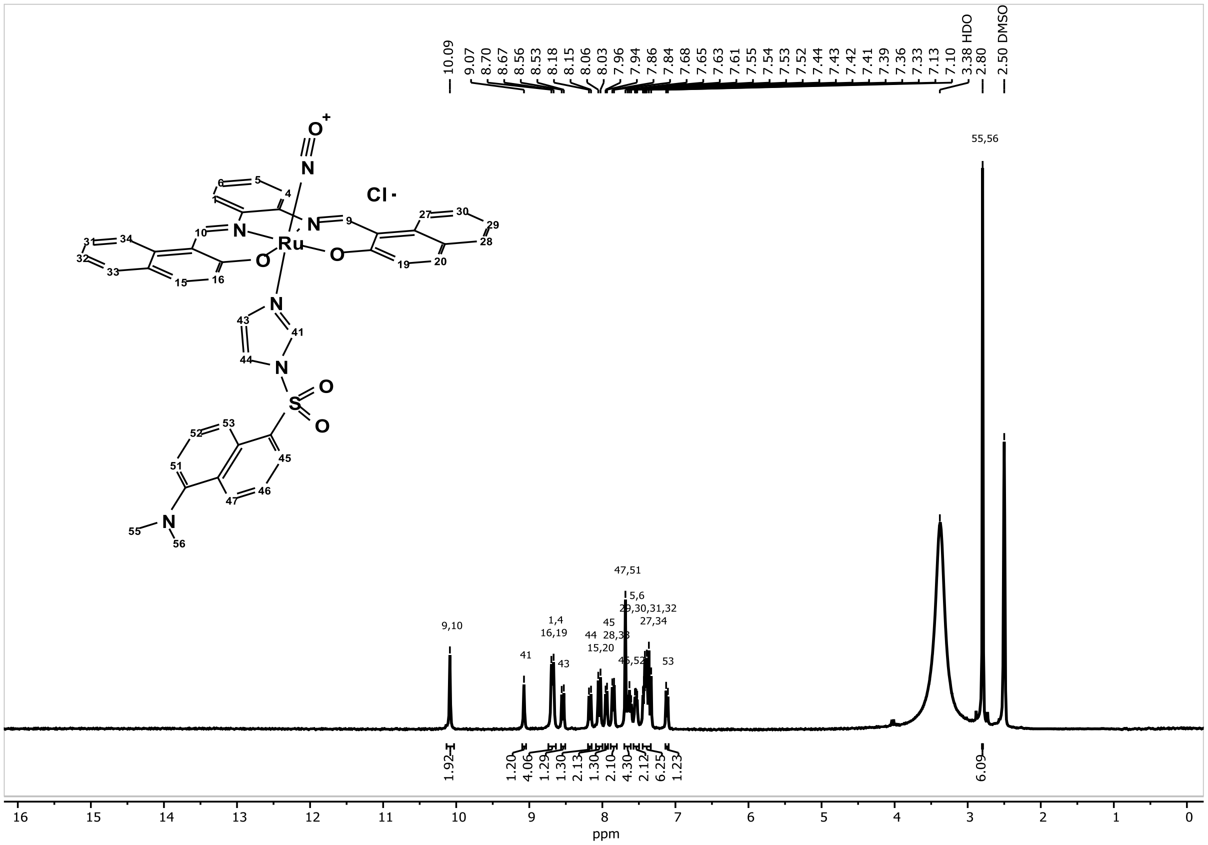

Figure S6 ^{1}H NMR (300 MHz) spectrum of **[RuL(NO)(ID)]Cl** in DMSO-d_6.

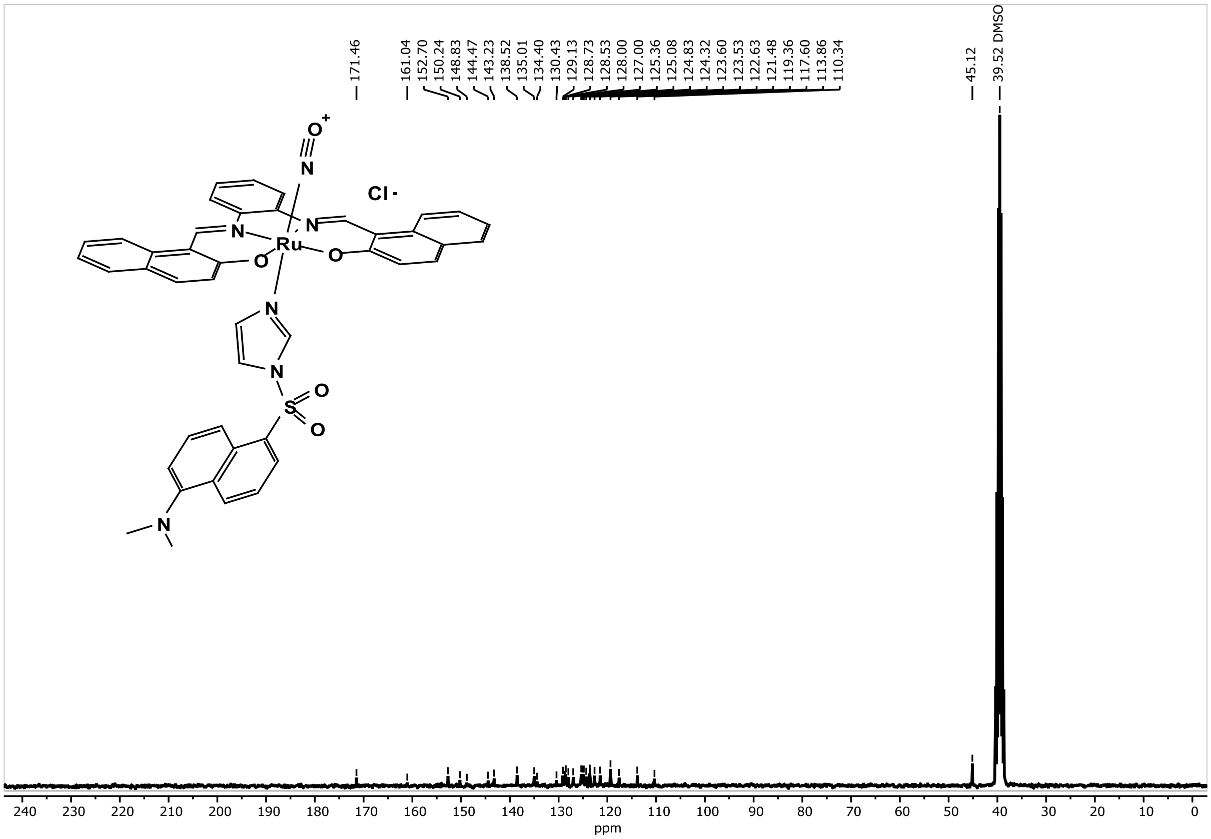

Figure S7 ^{13}C NMR (75 MHz) spectrum of **[RuL(NO)(ID)]Cl** in DMSO-d$_6$.

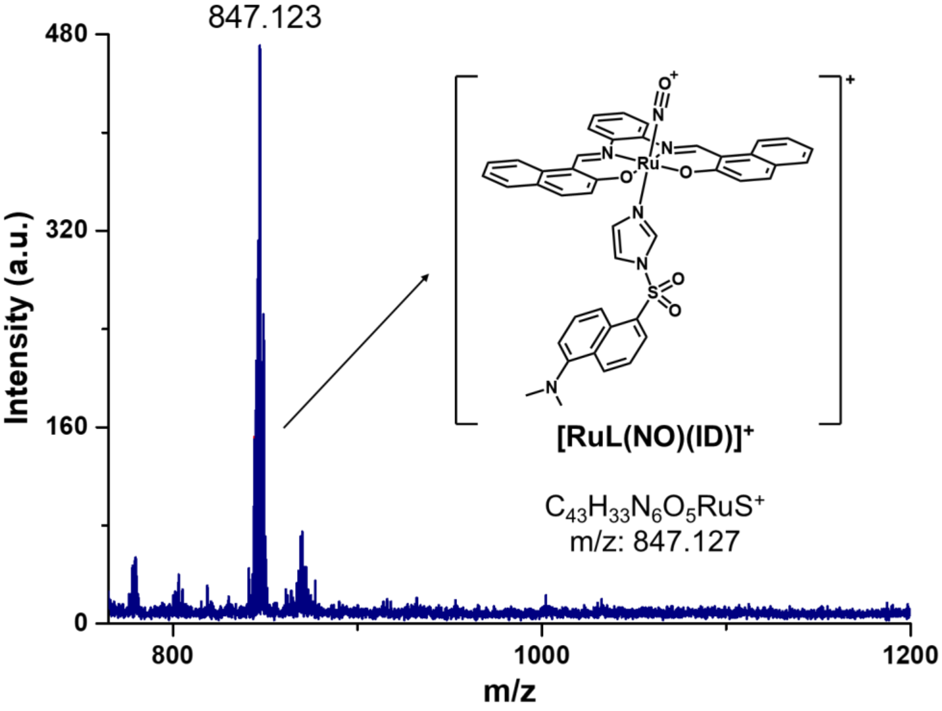

Figure S8 Positive scan MS MALDI-TOF spectrum of **[RuL(NO)(ID)][Cl]** in MeCN.

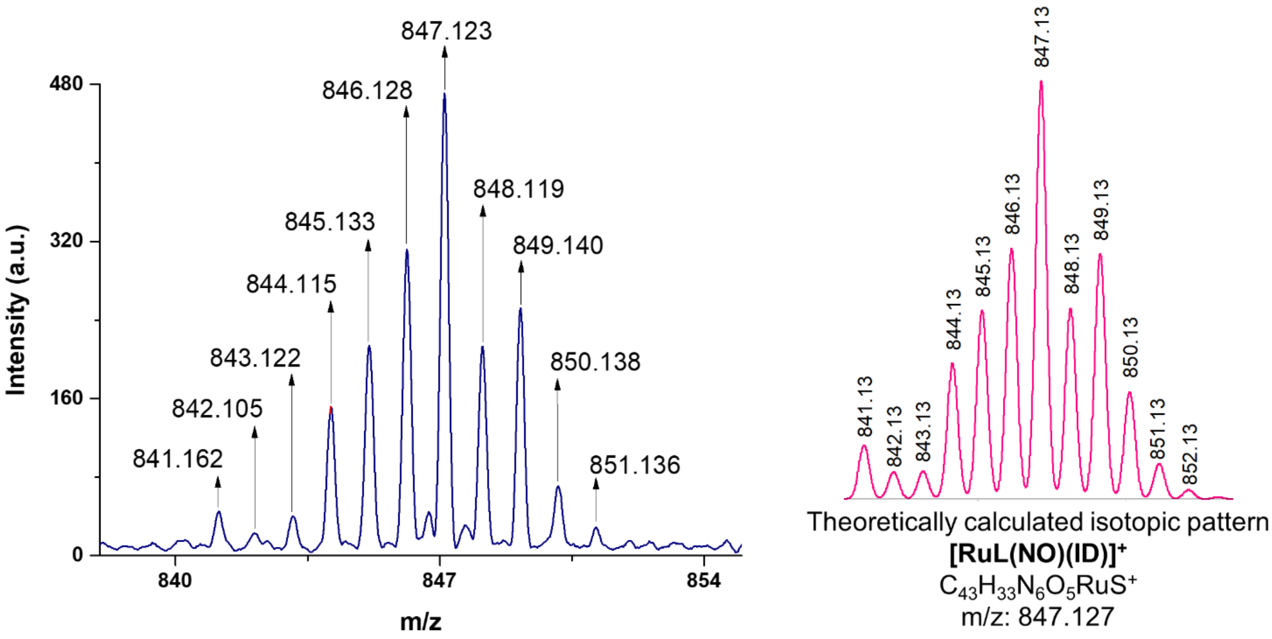

Figure S9 Partial positive scan MS MALDI-TOF spectrum of **[RuL(NO)(ID)][Cl]** in MeCN.

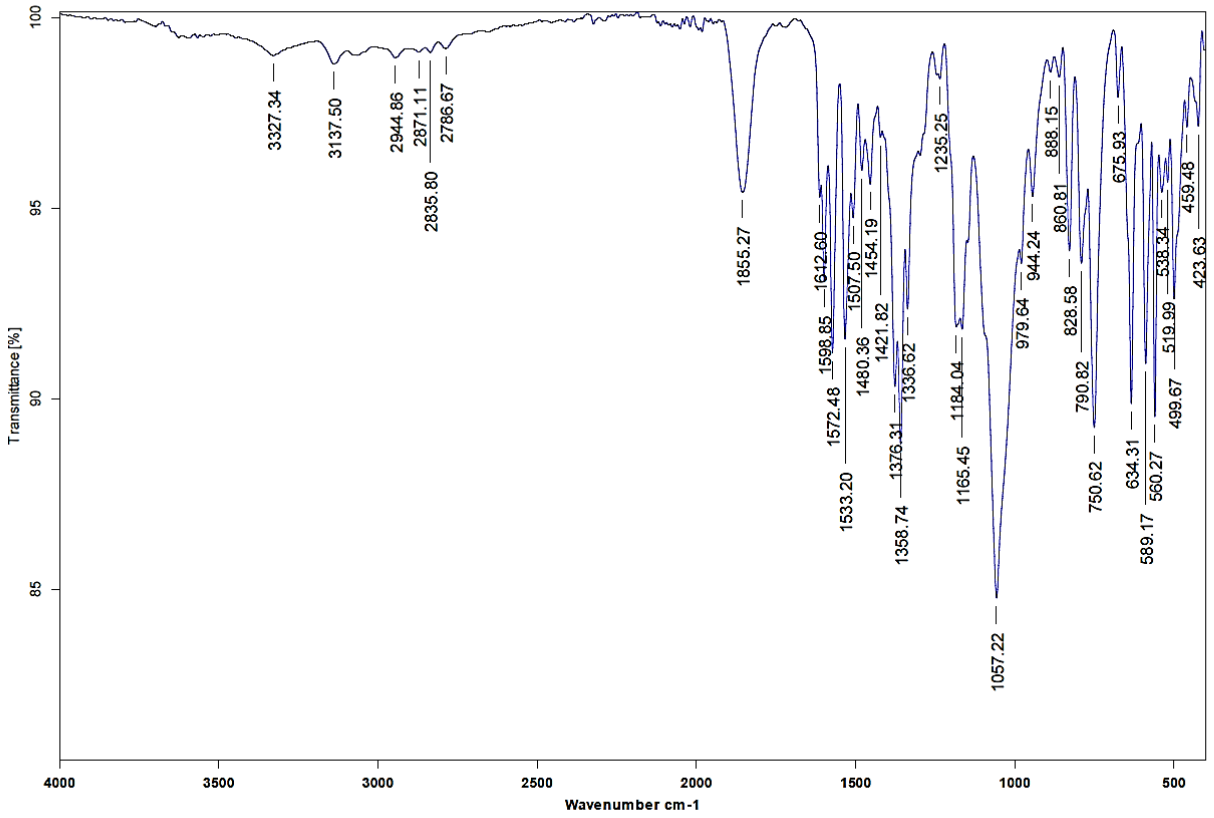

Figure S10 IR (ATR) spectrum of **[RuL(NO)(ID)]Cl**.

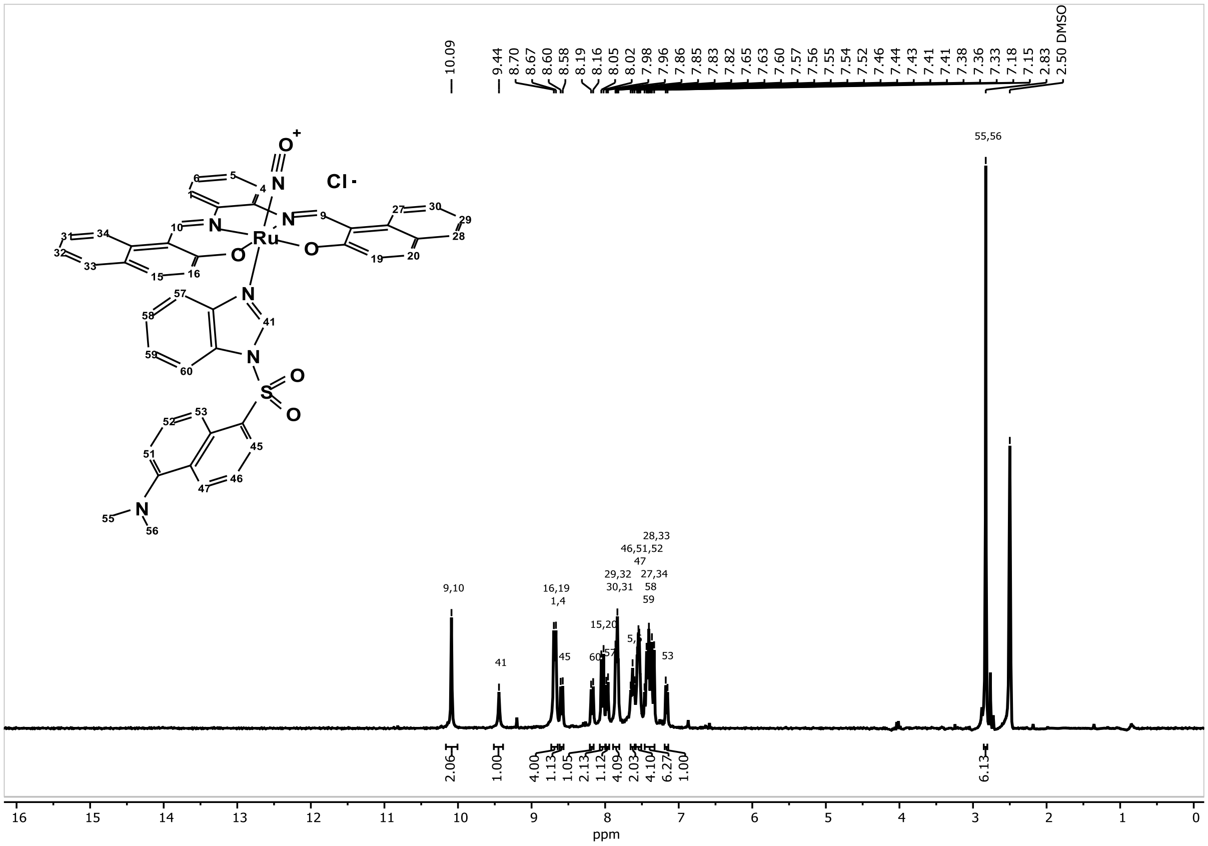

Figure S11 ^{1}H NMR (300 MHz) spectrum of **[RuL(NO)(BD)]Cl** in DMSO-d_6.

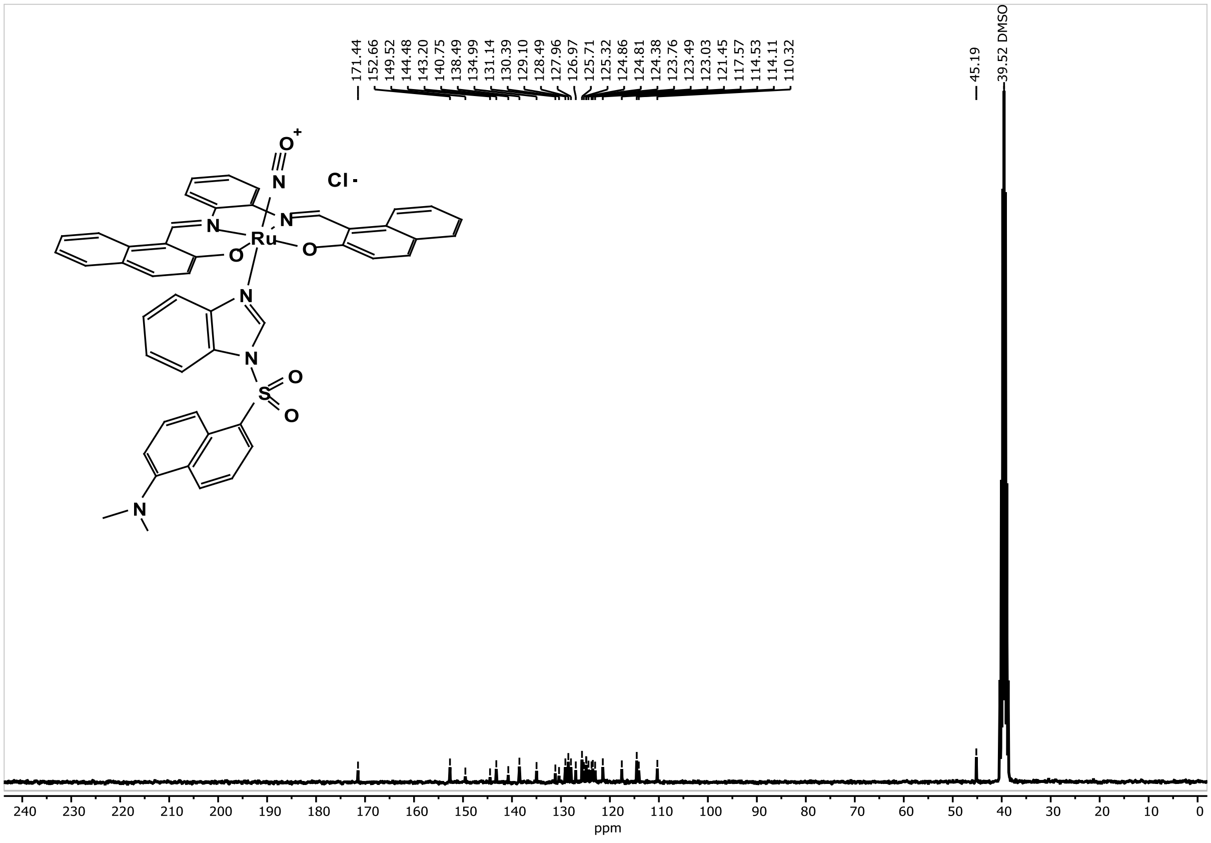

Figure S12 ^{13}C NMR (75 MHz) spectrum of **[RuL(NO)(BD)]Cl** in DMSO-d_6.

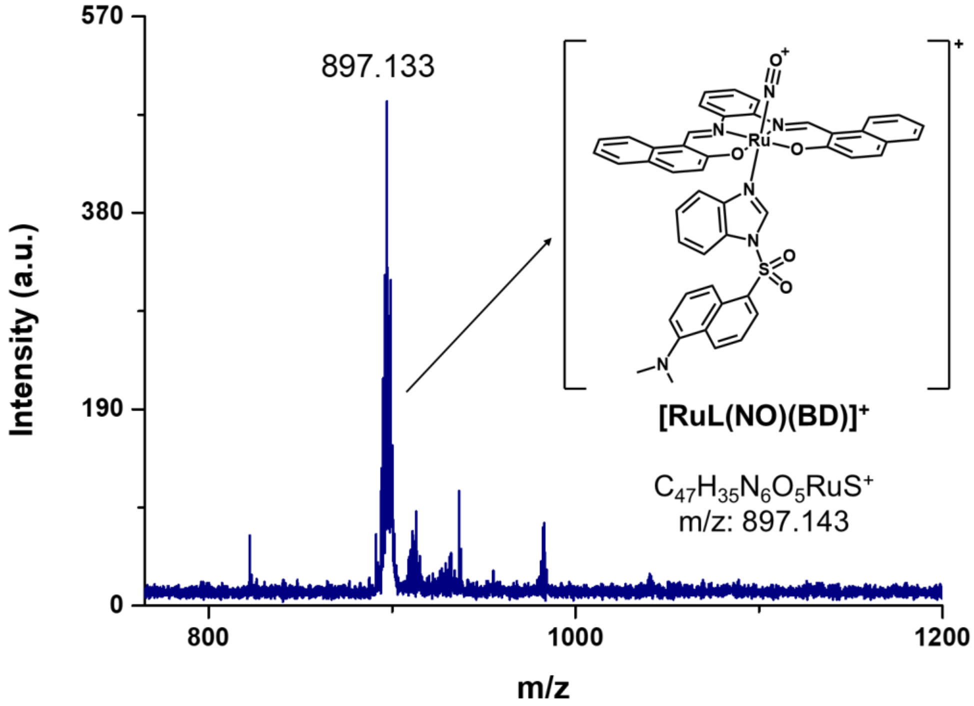

Figure S13 Positive scan MS MALDI-TOF spectrum of **[RuL(NO)(BD)][Cl]** in MeCN.

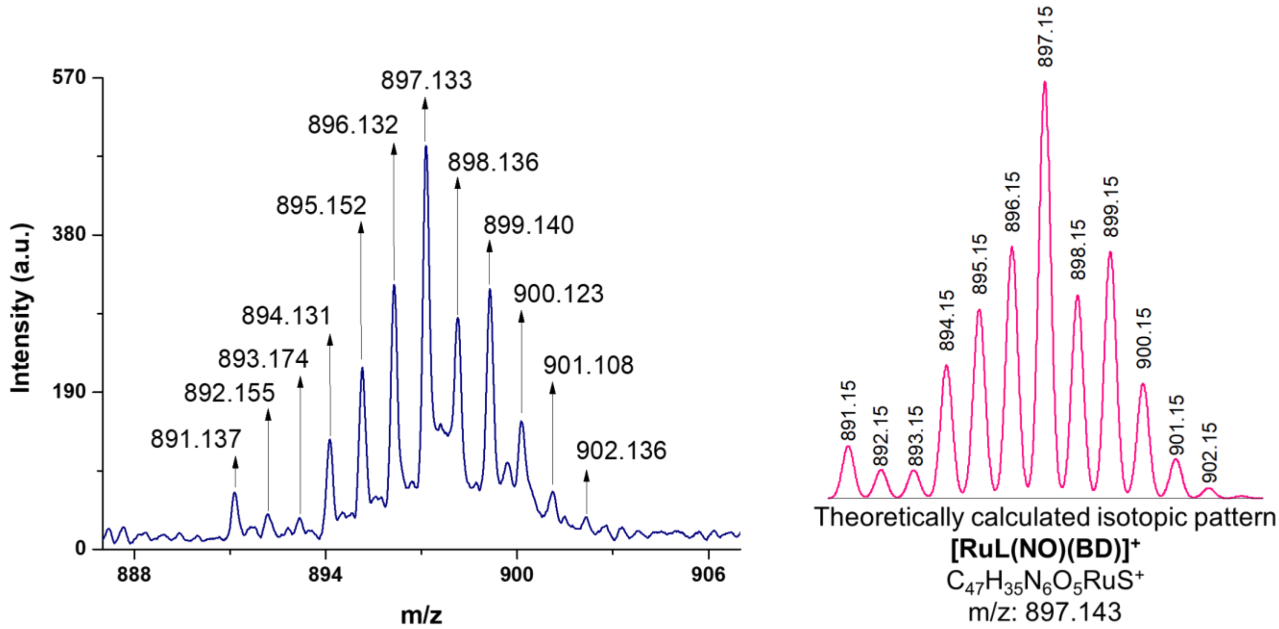

Figure S14 Partial positive scan MS MALDI-TOF spectrum of **[RuL(NO)(BD)][Cl]** in MeCN.

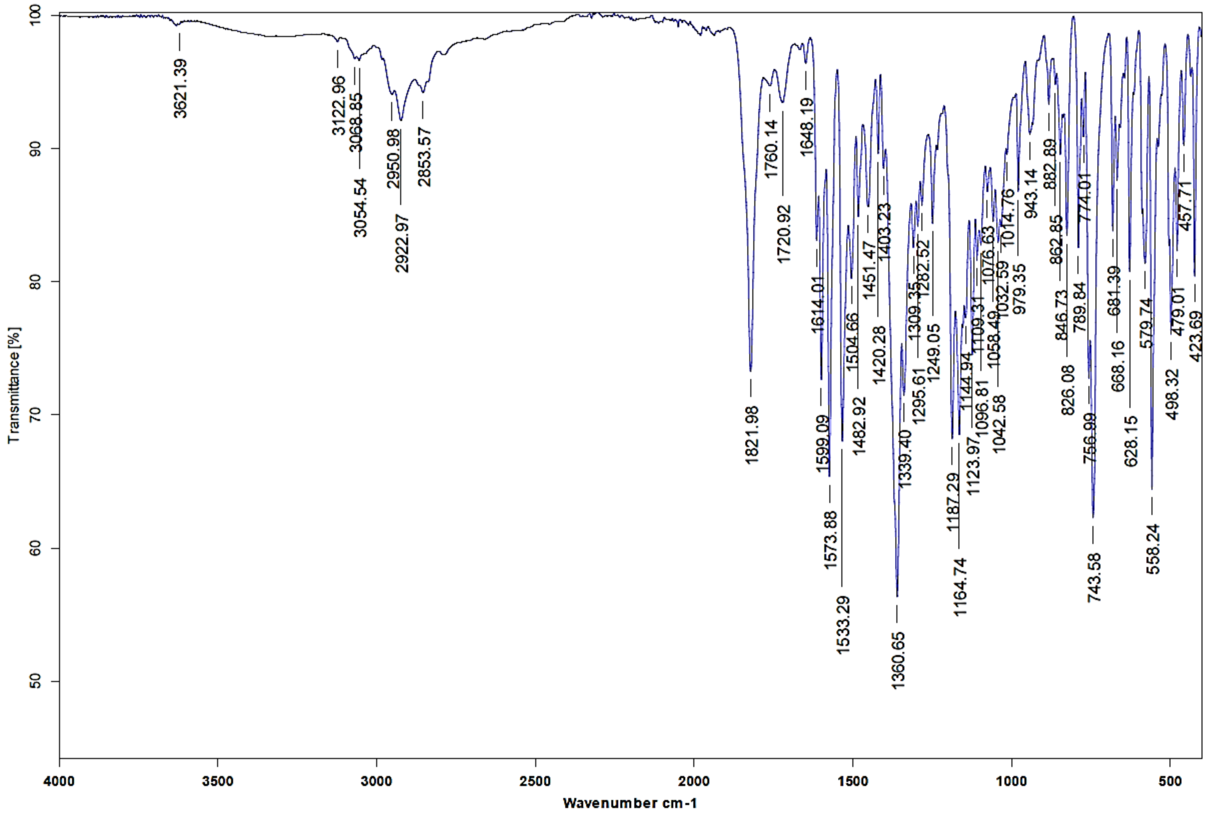

Figure S15 IR (ATR) spectrum of **[RuL(NO)(BD)]Cl**.

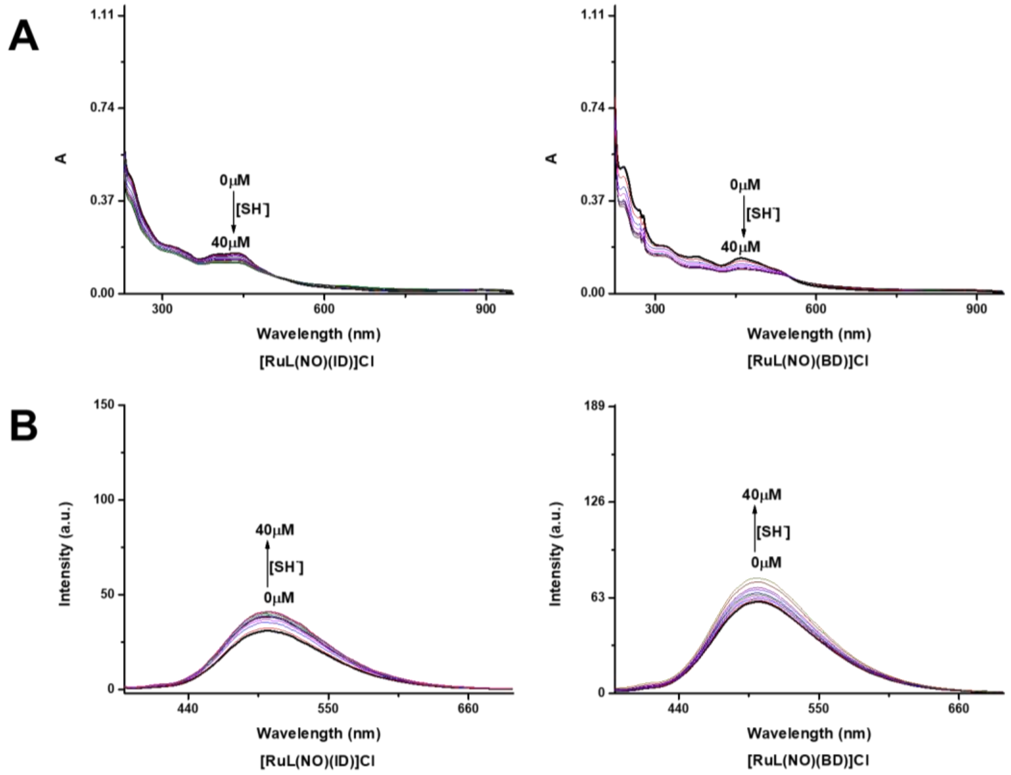

Figure S16 (A) UV/Vis absorption spectra and (B) emission spectra (λ_{ex} = 340 nm) of **[RuL(NO)(ID)]Cl** and **[RuL(NO)(BD)]Cl** solutions (10 μM) upon the addition of increasing amounts of NaSH at pH 7.4 (HEPES 20 mM, 25 °C).

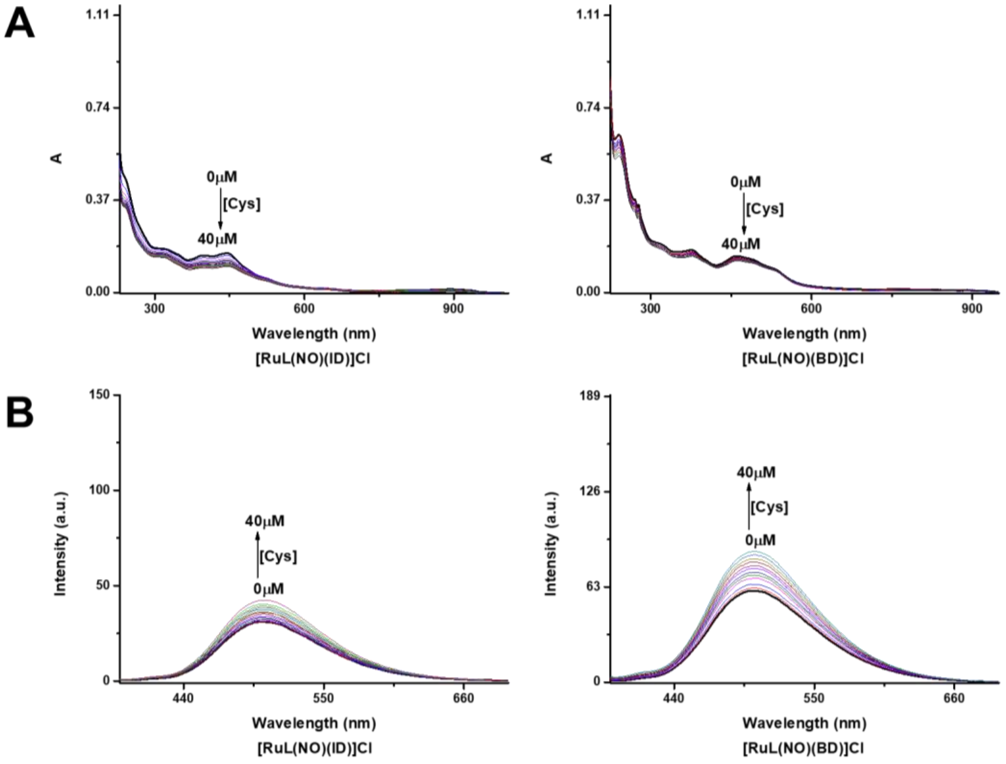

Figure S17 (A) UV/Vis absorption spectra and (B) emission spectra (λ_{ex} = 340 nm) of **[RuL(NO)(ID)]Cl** and **[RuL(NO)(BD)]Cl** solutions (10 μM) upon the addition of increasing amounts of Cys at pH 7.4 (HEPES 20 mM, 25 °C).

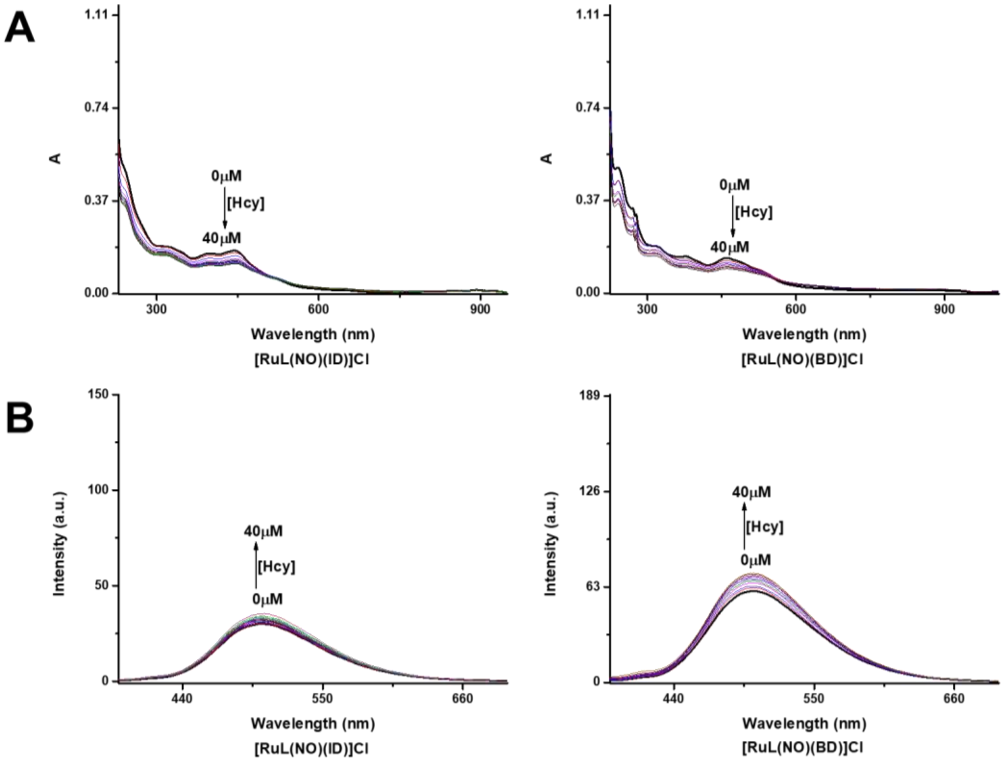

Figure S18 (A) UV/Vis absorption spectra and (B) emission spectra (λ_{ex} = 340 nm) of **[RuL(NO)(ID)]Cl** and **[RuL(NO)(BD)]Cl** solutions (10 μM) upon the addition of increasing amounts of Hcy at pH 7.4 (HEPES 20 mM, 25 °C).

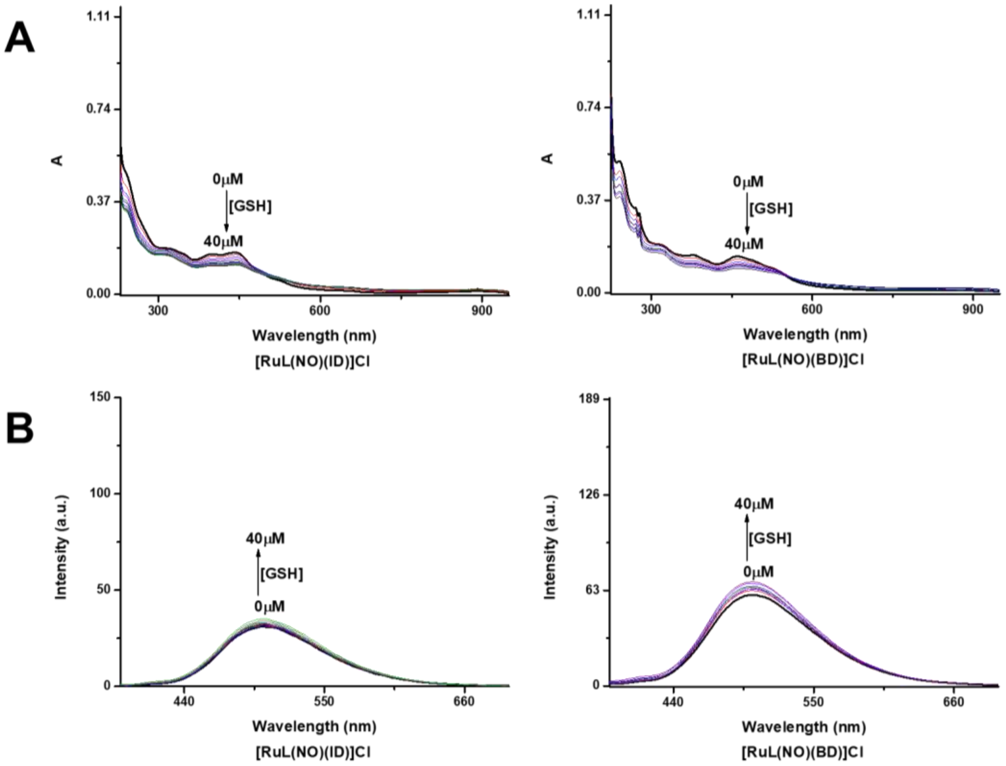

Figure S19 (A) UV/Vis absorption spectra and (B) emission spectra (λ_{ex} = 340 nm) of **[RuL(NO)(ID)]Cl** and **[RuL(NO)(BD)]Cl** solutions (10 μM) upon the addition of increasing amounts of GSH at pH 7.4 (HEPES 20 mM, 25 °C).

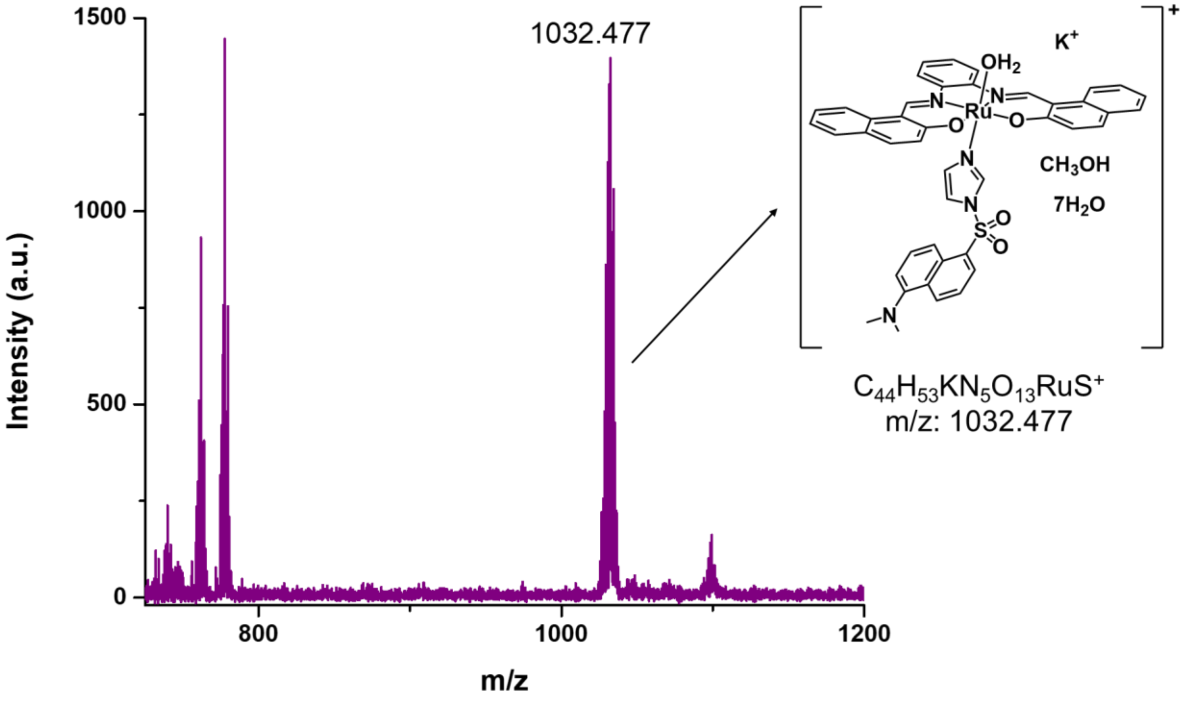

Figure S20 Positive scan MS MALDI-TOF spectrum of **[RuL(NO)(ID)][Cl]** after treatment with 4.0 equiv. of Sec in MeOH-H_2O (2:1 v/v).

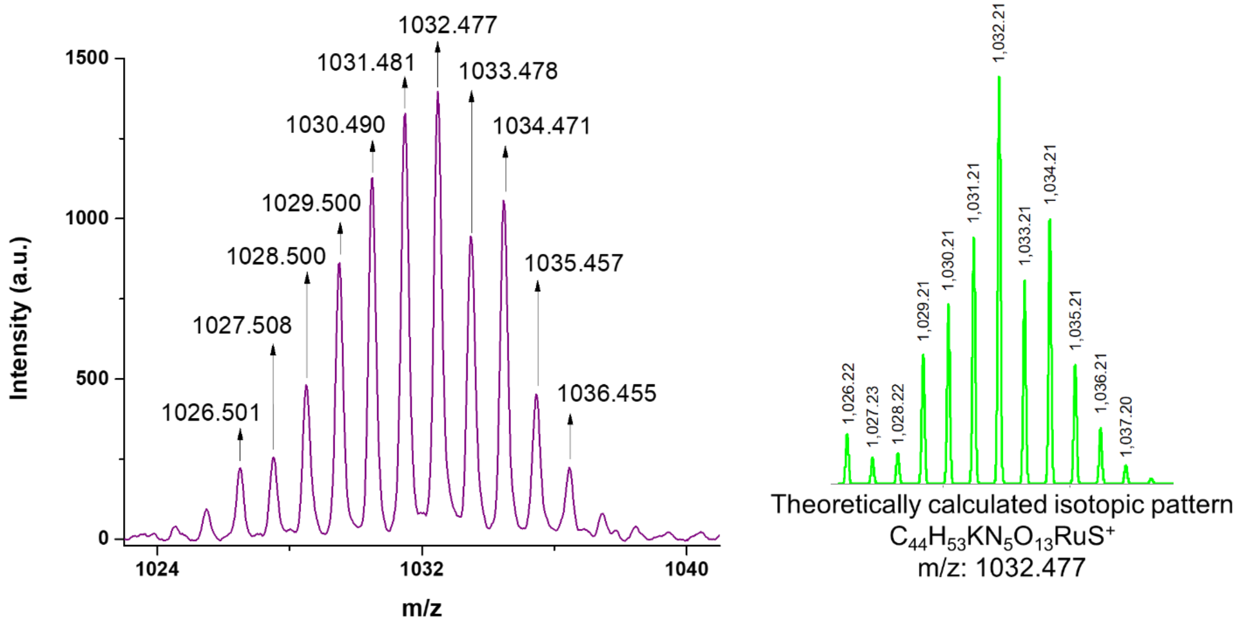

Figure S21 Partial positive scan MS MALDI-TOF spectrum of **[RuL(NO)(ID)][Cl]** after treatment with 4.0 equiv. of Sec in MeOH-H_2O (2:1 v/v).

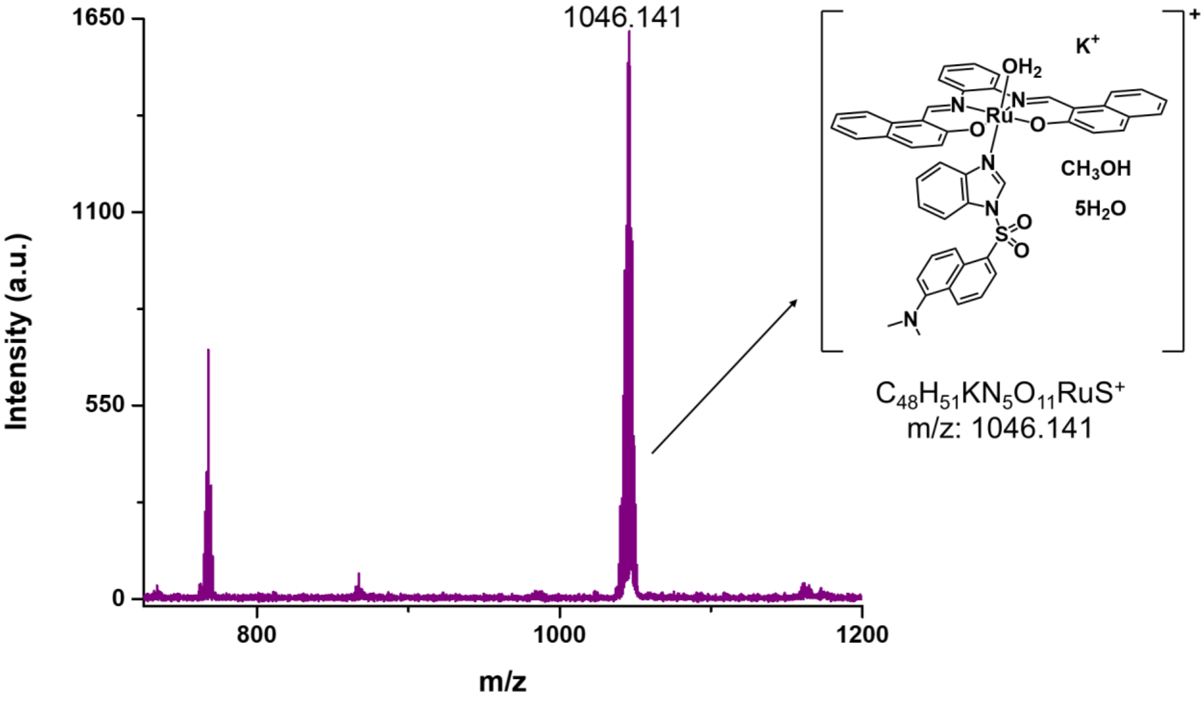

Figure S22 Positive scan MS MALDI-TOF spectrum of **[RuL(NO)(BD)][Cl]** after treatment with 4.0 equiv. of Sec in MeOH-H_2O (2:1 v/v).

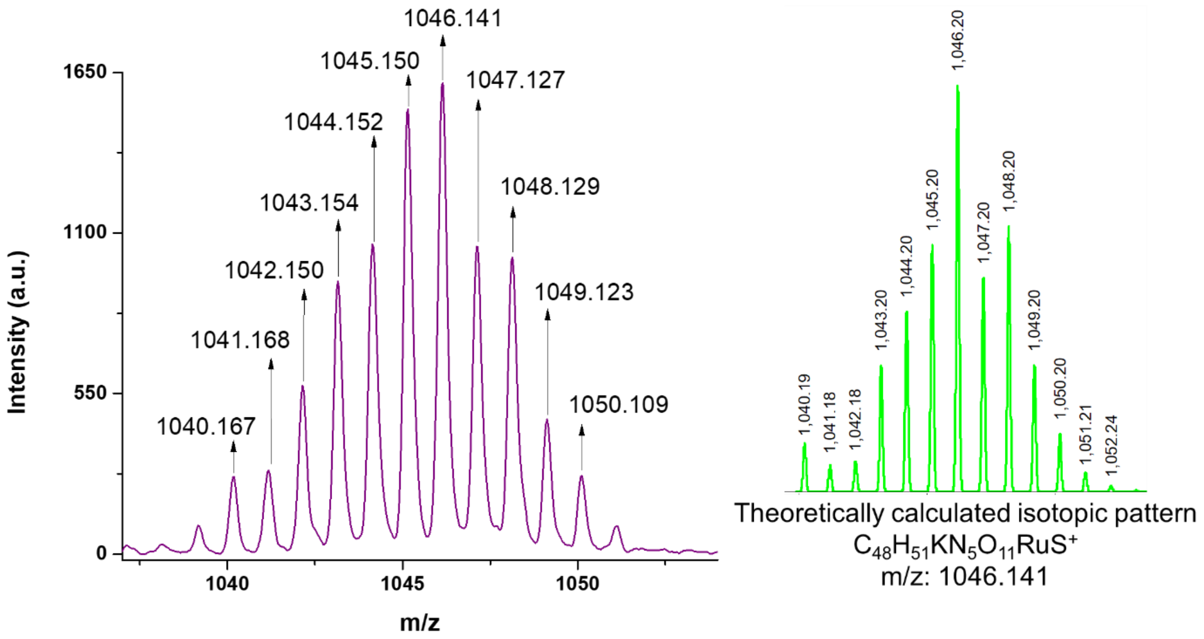

Figure S23 Partial positive scan MS MALDI-TOF spectrum of **[RuL(NO)(BD)][Cl]** after treatment with 4.0 equiv. of Sec in MeOH-H_2O (2:1 v/v).

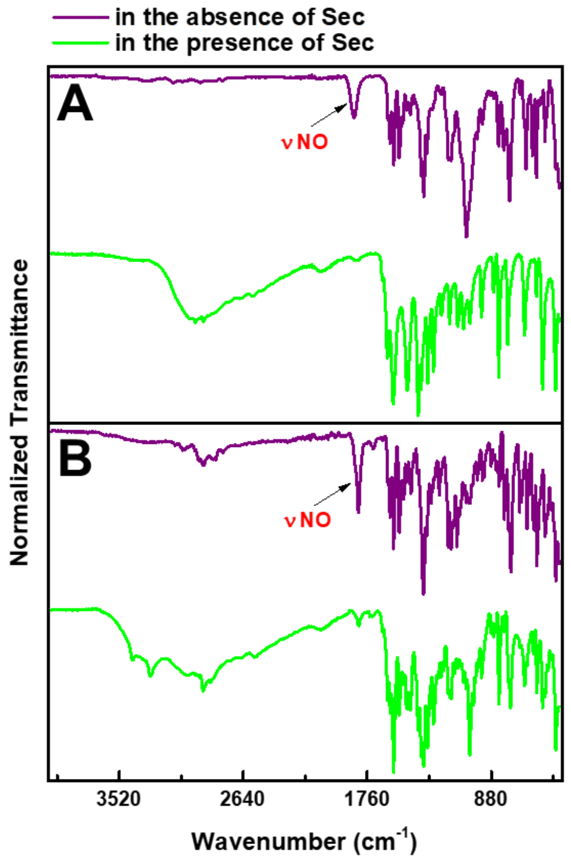

Figure S24 IR spectra of (A) **[RuL(NO)(ID)][Cl]** and (B) **[RuL(NO)(BD)][Cl]** before and after treatment with 4.0 equiv. of Sec

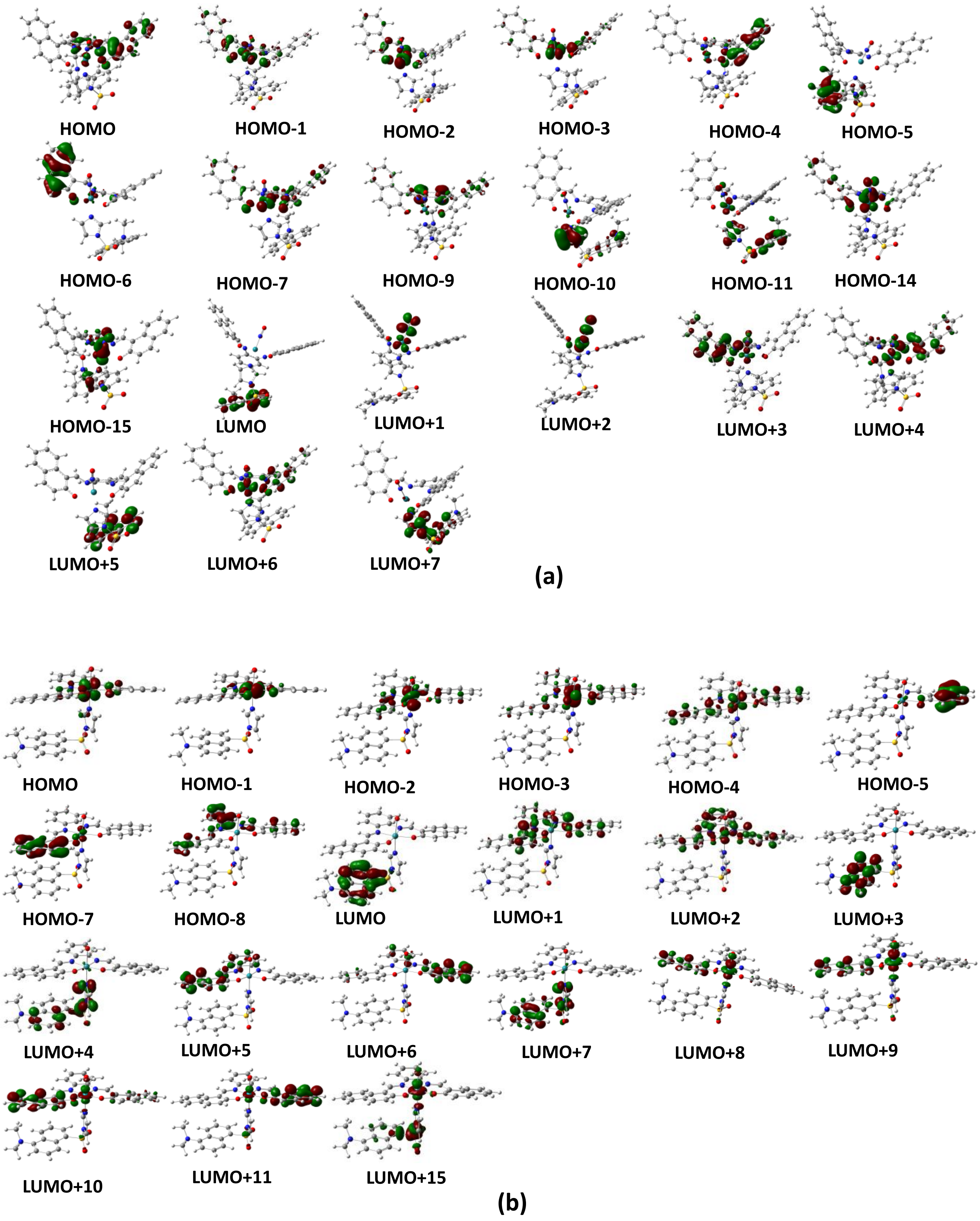

Figure S25 Molecular orbital: a) **[RuL(NO)(ID)]$^+$**, b) **[RuL(OH$_2$)(ID)]** at gaseous state.

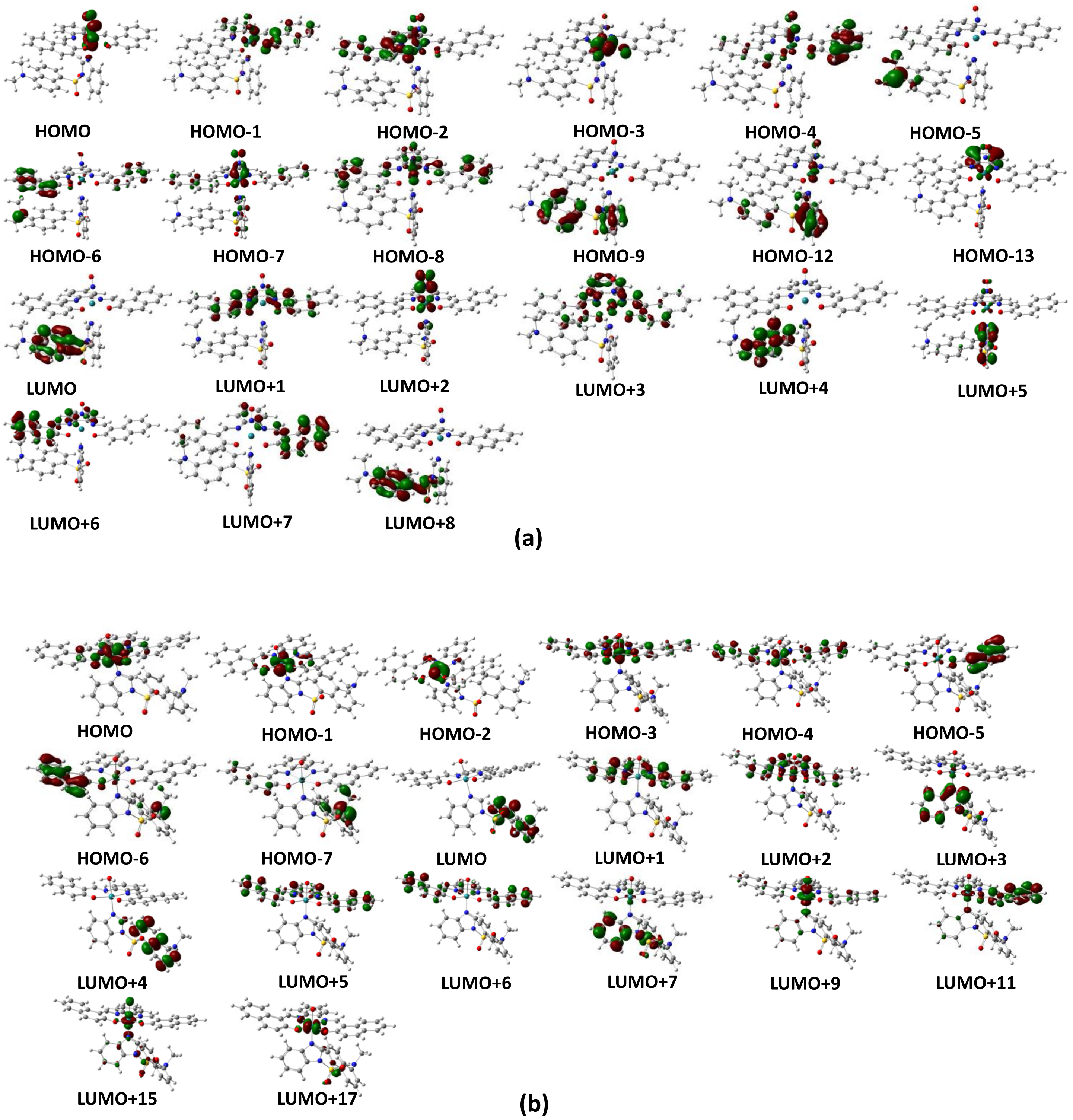

Figure S26 Molecular orbital: a) **[RuL(NO)(BD)]$^+$**, b) **[RuL(OH_2)(BD)]** at gaseous state.

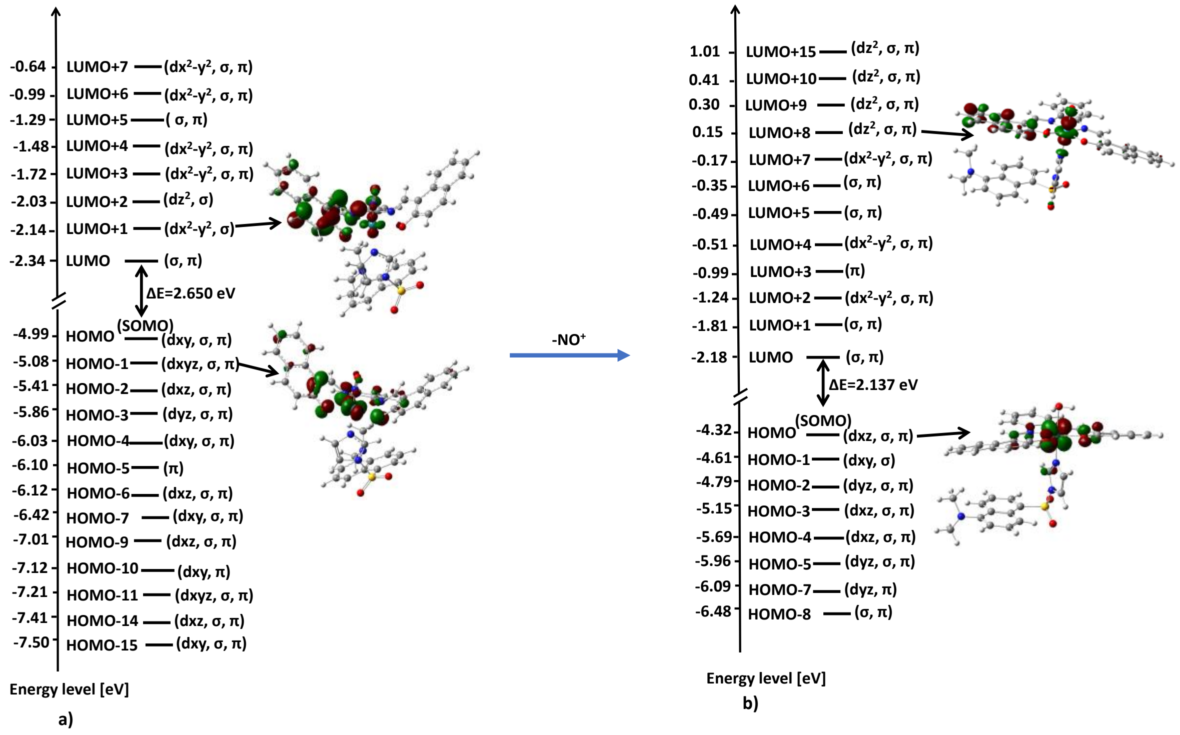

Figure S27 Frontier molecular orbital energy level diagram: a) **[RuL(NO)(ID)]$^+$** and b) its adduct **[RuL(OH_2)(ID)]** at gaseous state.

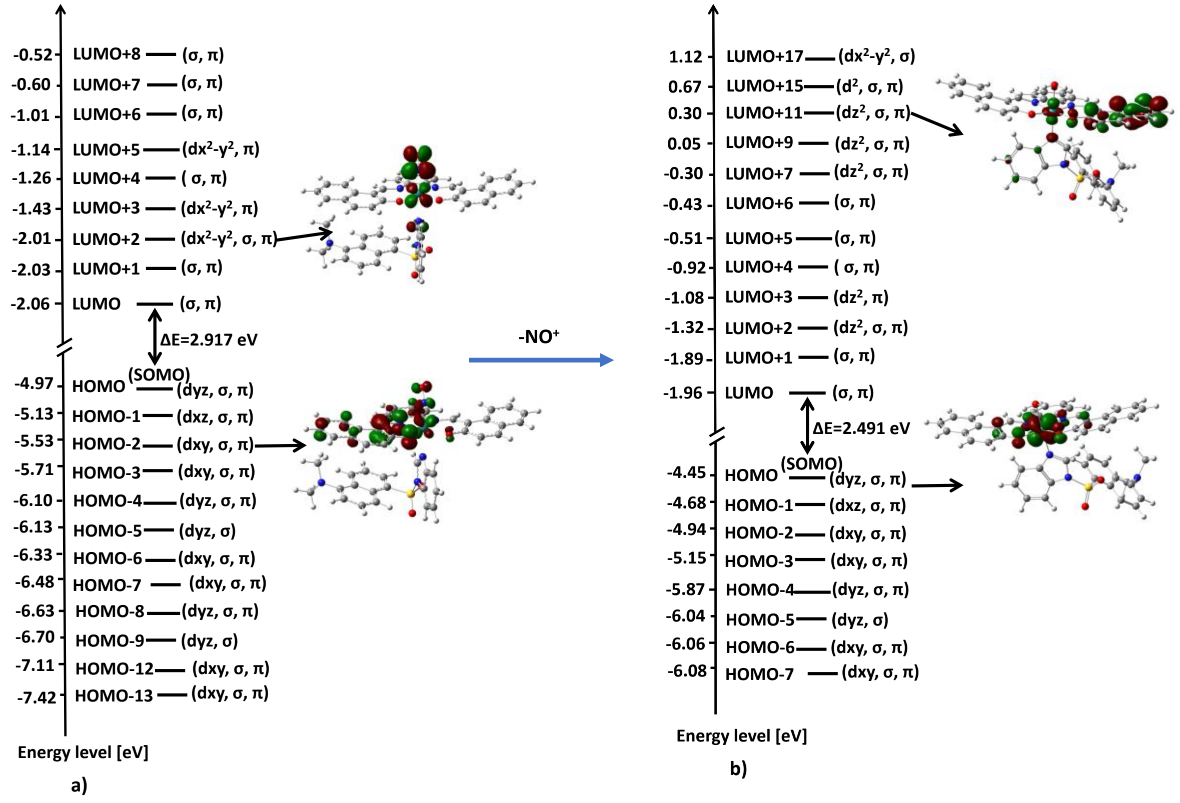

Figure S28 Frontier molecular orbital energy level diagram: a) **[RuL(NO)(BD)]$^+$** and b) its adduct **[RuL(OH_2)(BD)]** at gaseous state.

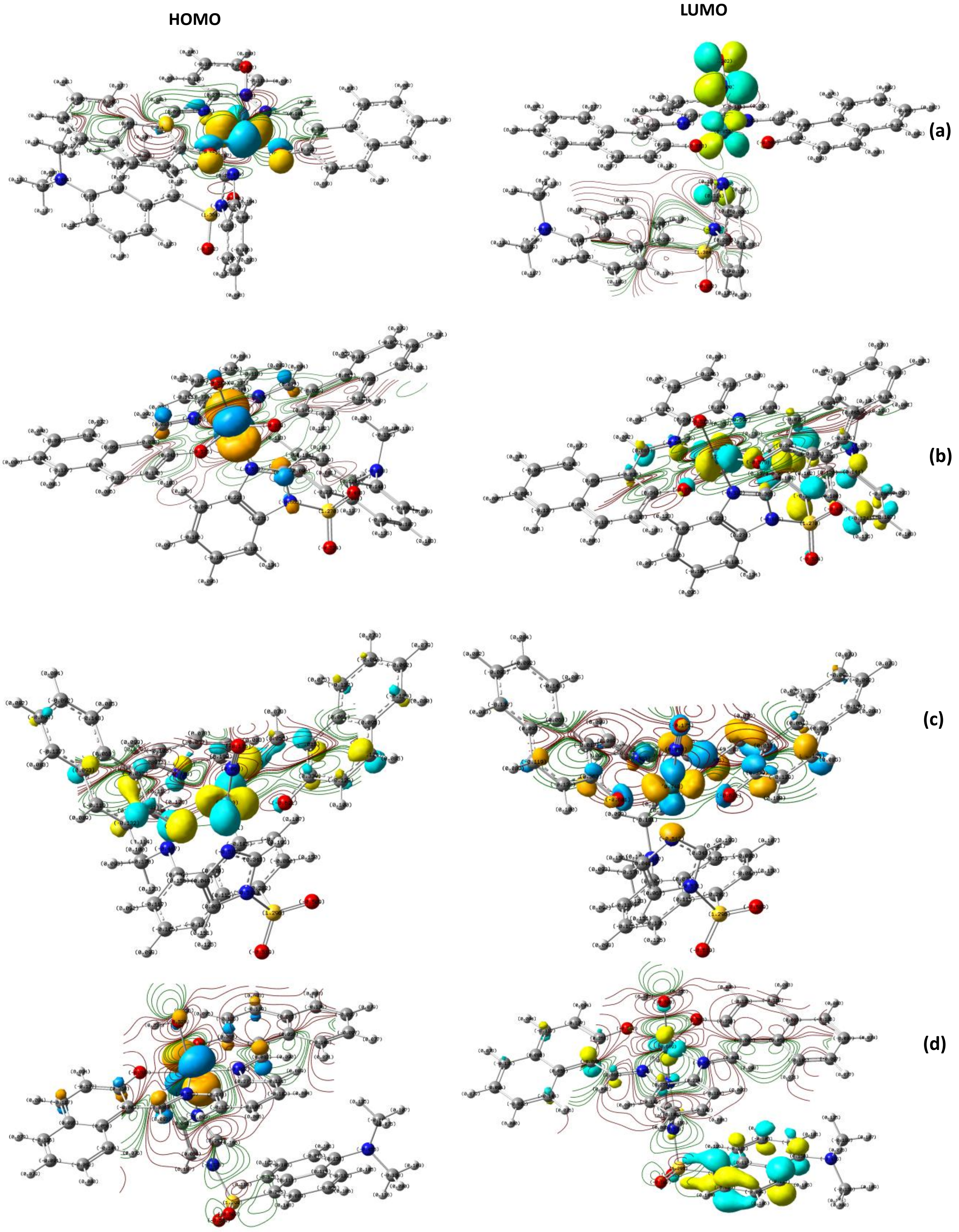

Figure S29. Visualization of the electron density isosurface determined by B3LYP/DGDZVP, HOMO and LUMO contour plots (isosurface value = 0.05 au) of the complexes: a) **[RuL(NO)(BD)]$^{+}$**, b) **[RuL(OH$_2$)(BD)]**, c) **[RuL(NO)(ID)]$^{+}$** and **[RuL(OH$_2$)(NO)]** at gaseous state.

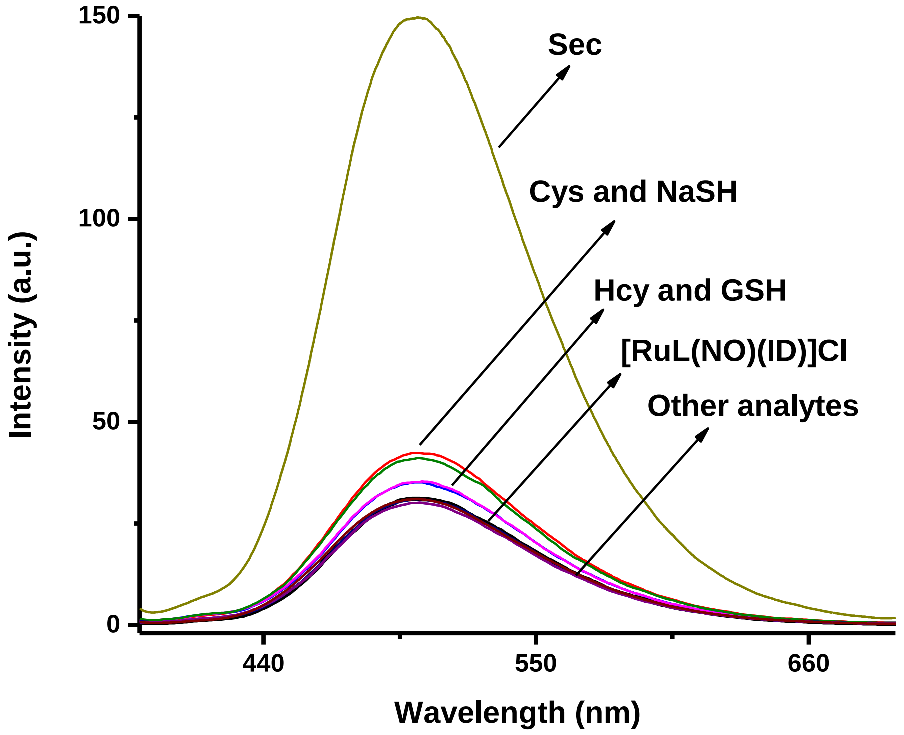

[RuL(NO)(ID)]Cl

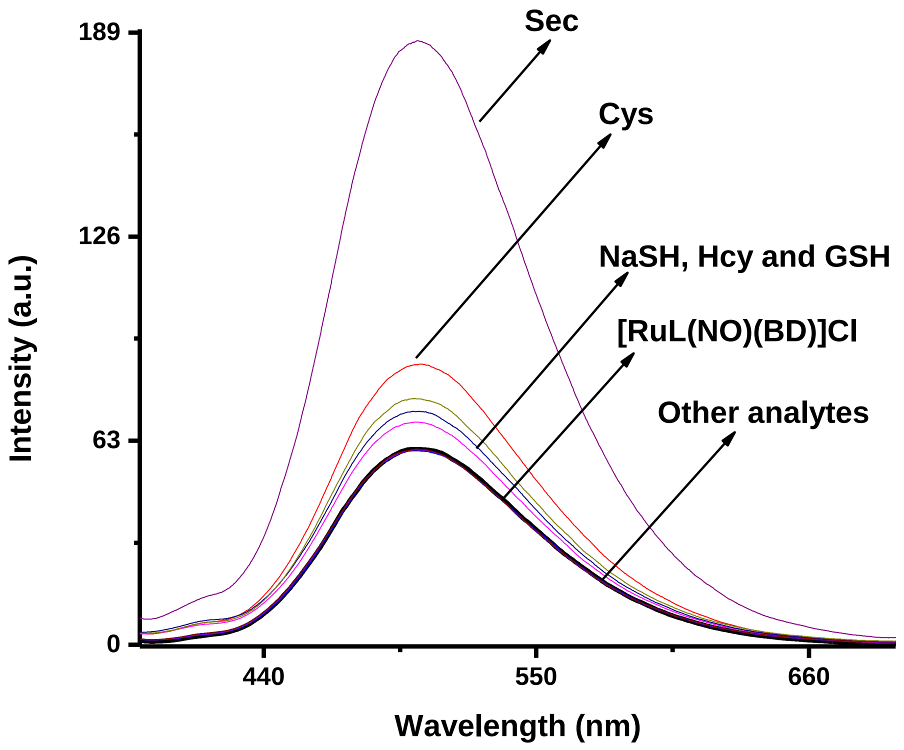

[RuL(NO)(BD)]Cl

Figure S30. The fluorescence enhancement of aqueous solution (10 μM) of **[RuL(NO)(ID)]Cl** and **[RuL(NO)(BD)]Cl** upon additions of different bioanalytes (40 μM) at pH 7.4 (HEPES 20 mM).

Table S1 Crystal data and structure refinement for **ID**.

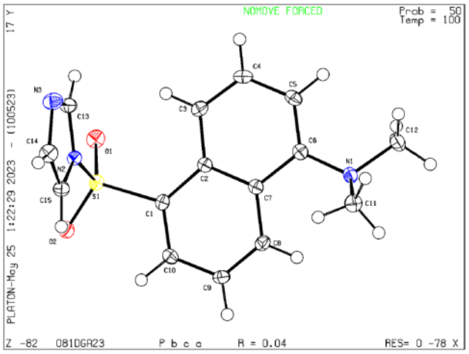

**Identification code**	081DGA23
**Empirical formula**	$C_{15} H_{15} N_3 O_2 S$
**Formula weight**	301.36
**Temperature**	100(2) K
**Wavelength**	0.71073 Å
**Crystal system**	Orthorhombic
**Space group**	Pbca
**Unit cell dimensions**	a = 16.3141(9) Å α= 90°.<br>b = 7.7734(4) Å β= 90°.<br>c = 22.0703(13) Å γ = 90°.
**Volume**	2798.9(3) $Å^3$
**Z**	8
**Density (calculated)**	1.430 Mg/m^3
**Absorption coefficient**	0.239 mm^{-1}
**F(000)**	1264
**Crystal size**	0.585 x 0.443 x 0.252 mm^3
**Theta range for data collection**	1.845 to 27.442°.
**Index ranges**	-21<=h<=21, -9<=k<=10, -28<=l<=28
**Reflections collected**	19099
**Independent reflections**	3185 [R(int) = 0.0472]
**Completeness to theta = 25.242°**	100.0 %
**Absorption correction**	None
**Refinement method**	Full-matrix least-squares on F^2
**Data / restraints / parameters**	3185 / 0 / 192
**Goodness-of-fit on F^2**	1.030
**Final R indices [I>2sigma(I)]**	R1 = 0.0355, wR2 = 0.0904
**R indices (all data)**	R1 = 0.0415, wR2 = 0.0951
**Extinction coefficient**	n/a
**Largest diff. peak and hole**	0.468 and -0.401 e.$Å^{-3}$

Table S2 Crystal data and structure refinement for **BD**.

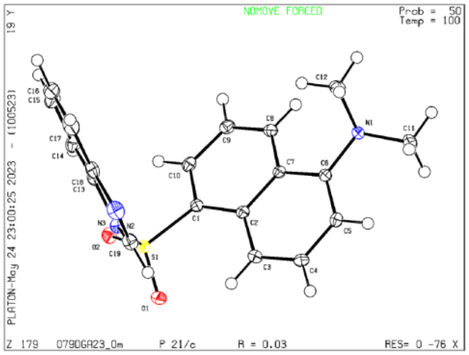

**Identification code**	079DGA23_0m
**Empirical formula**	C_{19} H_{17} N_3 O_2 S
**Formula weight**	351.41
**Temperature**	100(2) K
**Wavelength**	0.71073 Å
**Crystal system**	Monoclinic
**Space group**	$P2_1/c$
**Unit cell dimensions**	a = 15.6246(7) Å α= 90°.<br>b = 6.7563(3) Å β= 114.3299(8)°.<br>c = 16.4775(7) Å γ = 90°.
**Volume**	1584.96(12) Å^3
**Z**	4
**Density (calculated)**	1.473 Mg/m^3
**Absorption coefficient**	0.223 mm^{-1}
**F(000)**	736
**Crystal size**	0.555 x 0.393 x 0.331 mm^3
**Theta range for data collection**	2.492 to 27.445°.
**Index ranges**	-20<=h<=20, -8<=k<=8, -21<=l<=21
**Reflections collected**	16707
**Independent reflections**	3610 [R(int) = 0.0328]
**Completeness to theta = 25.242°**	99.7 %
**Absorption correction**	None
**Refinement method**	Full-matrix least-squares on F^2
**Data / restraints / parameters**	3610 / 0 / 228
**Goodness-of-fit on F^2**	1.024
**Final R indices [I>2sigma(I)]**	R1 = 0.0324, wR2 = 0.0852
**R indices (all data)**	R1 = 0.0340, wR2 = 0.0865
**Extinction coefficient**	n/a
**Largest diff. peak and hole**	0.372 and -0.452 e.Å^{-3}

Table S3 Bond lengths (Å) and bond angles (°) resulted for nitrosyl complexes and aquo-complexes, using functional B3LYP/6-31G**, LANL2DZ basis set at level of theory (gaseous state).

**Functional**	**B3LYP/DGDZVP**				
**Bond length complexes (Å)**	Complexes		**Bond length complexes (Å)**	Complexes	
	**$[RuL(NO)(BD)]^+$**	**$[RuL(OH_2)(BD)]$**		**$[RuL(NO)(ID)]^+$**	**$[RuL(OH_2)(ID)]$**
Ru-N1	2.031	2.021	Ru-N1	2.415	2.021
Ru-N2	2.030	2.024	Ru-N2	2.878	2.020
Ru-N3	2.240	2.090	Ru-N3	2.158	2.055
Ru-N4	1.879	-	Ru-N4	1.759	-
Ru-O1	2.093	2.100	Ru-O1	2.096	2.101
Ru-O2	2.093	2.109	Ru-O2	2.092	2.100
Ru-O3	-	2.242	Ru-O3	-	2.250
**Bond Angle (°)**			O1-H2	-	2.257
N1-Ru-N2	82.8	83.1	O2-H3	-	2.282
N1-Ru-N3	89.2	94.7	O4-H1N7	-	-
N1-Ru-N4	90.8	-	**Bond Angle (°)**		-
N1-Ru-O1	172.7	170.4	N1-Ru-N2	61.8	83.2
N1-Ru-O2	91.8	90.7	N1-Ru-N3	91.5	92.1
N1-Ru-O3	-	92.5	N1-Ru-N4	100.6	-
N2-Ru-N3	89.6	-	N1-Ru-O1	124.6	172.8
N2-Ru-N4	90.4	-	N1-Ru-O2	76.8	91.7
N2-Ru-O1	91.9	90.8	N1-Ru-O3	-	95.1
N2-Ru-O2	172.6	171.3	Ru-N4-O3	-	-
N2-Ru-O3	-	94.6	Ru-N4-Se1	-	-
N3-Ru-N4	180.0	-	Ru-N4-S2	-	-
N3-Ru-O1	85.8	92.7	N2-Ru-N3	85.8	91.6
N3-Ru-O2	85.2	97.7	N2-Ru-N4	102.2	-
N3-Ru-O3	-	172.3	N2-Ru-O1	62.9	91.6
N4-Ru-O1	94.2	-	N2-Ru-O2	136.8	173.3
N4-Ru-O2	94.8	-	N2-Ru-O3	-	95.2
O1-Ru-O2	93.0	94.3	N3-Ru-N4	167.5	-
O1-Ru-O3	-	80.4	N3-Ru-O1	82.2	93.0
O2-Ru-O3	-	79.5	N3-Ru-O2	83.7	92.9
Ru-N4-Se	-	-	N3-Ru-O3	-	170.6
Ru-N4-O3	-	-	N4-Ru-O1	93.0	-
Ru-N4-S2	-	-	N4-Ru-O2	96.4	-
			O1-Ru-O2	154.6	93.2
			O1-Ru-O3	-	80.4
			O2-Ru-O3	-	80.9
